# Ultra-processed foods, lifestyle management, and cardiovascular diseases: A clinical consensus statement of the European Society of Cardiology Council for Cardiology Practice and the European Association of Preventive Cardiology of the European Society of Cardiology

**DOI:** 10.1093/eurheartj/ehag226

**Published:** 2026-05-06

**Authors:** Luigina Guasti, Marialaura Bonaccio, Ana Abreu, Riccardo Asteggiano, Maira Bes-Rastrollo, Ruxandra Christodorescu, Giovanni de Gaetano, Marc Ferrini, Pedro Marques-Vidal, Atul Pathak, Dimitri Richter, Sukshma Sharma, Catarina Sousa Guerreiro, Bernard Srour, Saverio Stranges, Mathilde Touvier, Branislav Vohnout, Massimo Piepoli, Licia Iacoviello

**Affiliations:** Internal Medicine, Department of Medicine and Surgery, University of Insubria, Via Guicciardini 5, Varese 21100, Italy; Division of Geriatrics and Clinical Gerontology, ASST-Settelaghi, Varese, Italy; Research Unit of Epidemiology and Prevention, IRCCS NEUROMED, Pozzilli, IS 86077, Italy; Centre of Cardiovascular Rehabilitation Cardiology Department, Centro Universitário Hospitalar Lisboa Norte & Faculdade de Medicina da Universidade Lisboa/Instituto Saúde Ambiental & Instituto Medicina Preventiva, Faculdade Medicina da Universidade Lisboa/CCUL/CAML, Lisbon, Portugal; Internal Medicine, Department of Medicine and Surgery, University of Insubria, Via Guicciardini 5, Varese 21100, Italy; Poliambulatori Gruppo LARC-Laboratorio Analisi e Ricerca Clinica, Cardiology, Turin, Italy; Department of Preventive Medicine and Public Health, University of Navarra, Pamplona, Spain; IdiSNA, Navarra Institute for Health Research, Irunlarrea 3, Pamplona, Spain; CIBER Fisiopatología de La Obesidad y Nutrición, Madrid, Spain; Department V Internal Medicine, University of Medicine and Pharmacy V. Babes Timisoara, Timisoara, Romania; Institute of Cardiovascular Diseases Research Center, Timișoara, Romania; Research Unit of Epidemiology and Prevention, IRCCS NEUROMED, Pozzilli, IS 86077, Italy; Department of Cardiology and Vascular Pathology, CH Saint Joseph and Saint Luc, Lyon, France; Service de médecine interne, Département de médecine, Centre Hospitalier Universitaire Vaudois, Lausanne, Switzerland; Faculté de biologie et de médecine, Université de Lausanne, Lausanne, Switzerland; Institut national de Cardiologie, Chirurgie cardiaque et de Cardiologie interventionnelle (INCCI), National Institute of Cardiology, Cardiac Surgery and Interventional Cardiology 2A, Luxembourg, Brussels; Euroclinic Hospital, Athens, Greece; Department of Animal Science, Food and Nutrition (DIANA), Università Cattolica del Sacro Cuore, Piacenza, Italy; Laboratório de Nutrição, Faculdade de Medicina, Centro Académico de Medicina de Lisboa, Universidade de Lisboa, Lisboa, Portugal; Instituto de Saúde Ambiental, Faculdade de Medicina, Universidade de Lisboa, Lisboa, Portugal; Université Sorbonne Paris Nord and Université Paris Cité, Institut National de la Santé et de la Recherche Médicale (INSERM), Institut National de la Recherche pour l'Agriculture, l'Alimentation et l'Environnement, Conservatoire National des Arts et Métiers, Nutritional Epidemiology Research Team, Center of Research in Epidemiology and Statistics, Bobigny, France; Department of Epidemiology and Biostatistics, Schulich School of Medicine and Dentistry, Western University, London, ON, Canada; Department of Clinical Medicine and Surgery, University of Naples Federico II, Naples, Italy; Université Sorbonne Paris Nord and Université Paris Cité, Institut National de la Santé et de la Recherche Médicale (INSERM), Institut National de la Recherche pour l'Agriculture, l'Alimentation et l'Environnement, Conservatoire National des Arts et Métiers, Nutritional Epidemiology Research Team, Center of Research in Epidemiology and Statistics, Bobigny, France; Institute of Nutrition, FOaZOS, and Department of Diabetology, Faculty of Medicine, Slovak Medical University, Bratislava, Slovakia; Clinical Cardiology, IRCCS Policlinico San Donato, San Donato Milanese, Milan, Italy; Department of Biomedical Sciences for Health, University of Milan, Milan, Italy; Department of Medicine and Surgery, LUM University ‘Giuseppe Degennaro’, Casamassima, BA, Italy

**Keywords:** Ultra-processed foods, Nova classification, Diet quality, Cardiovascular disease, Cardiometabolic disease, Systematic review, Preventive cardiology

## Abstract

Ultra-processed foods (UPFs) have increasingly displaced traditional diets globally and have become a significant public health concern, particularly in relation to cardiovascular (CV) diseases. UPFs are defined as food products primarily composed of cheap industrial ingredients, additives, and neo-formed compounds, often with little to no nutritional value. These foods are highly processed and contain additives that can have harmful effects on health. While traditional dietary guidelines have long emphasized the importance of limiting animal-derived fats and promoting the intake of fruits, vegetables, and unsaturated fats, recent evidence suggests that the extent and nature of food processing are also key factors in the relationship between diet and health.

Studies over the past decade have highlighted that the consumption of UPFs is associated with increased CV risk, often independent of the overall diet quality.

Despite growing evidence linking UPF consumption to major CV risk factors (e.g. hypertension, dyslipidaemia, obesity) and adverse CV outcomes, the role of food processing in CV health remains underrecognized in cardiology. Current dietary counselling in clinical practice tends to overlook the potential adverse impact of UPFs, with patients not receiving comprehensive nutritional guidance.

This European Society of Cardiology (ESC) clinical consensus statement, developed by a multidisciplinary group of European experts, is conceived to increase awareness among clinicians about the CV risks associated with UPFs. Starting from a comprehensive review of current evidence, it provides practical, actionable advice to help the general cardiologists incorporate UPF-related assessment and counselling into their routine care. The statement also proposes a stepwise framework focused on CV prevention, including tools designed to enhance patient communication and engagement. Moreover, it discusses these clinical advices within wider strategic and policy frameworks, therefore supporting a more integrated, food-centred approach to improve CV health.

## Graphical Abstract

**Graphical Abstract:**
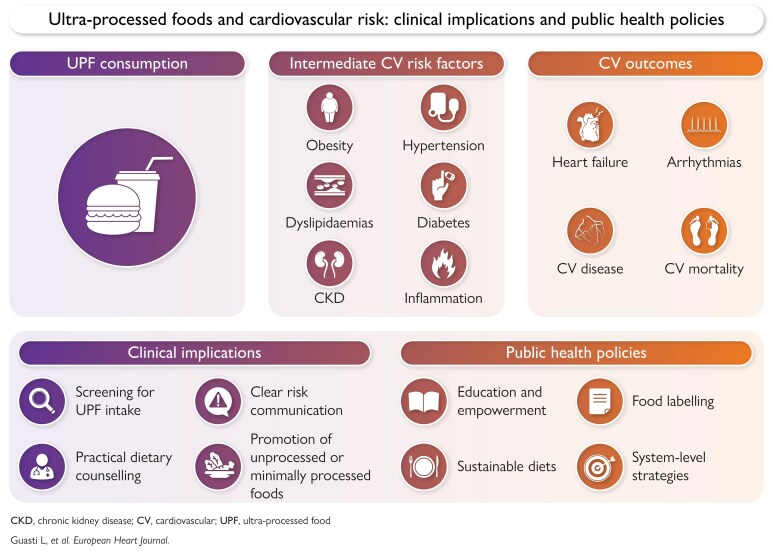
In a Cardiology setting, the clinical evaluation of nutritional habits and councelling should include ultra-processed food consumption, associated with worse cardiovascular outcomes. Public health policies need to take ultra-processed foods into account in order to reduce the risk accociated with these products.

## Introduction

Ultra-processed foods (UPFs) constitute an increasing part of the world’s food consumption and are becoming a worldwide challenge for population health.^1,2^

It is well established that both the quality and quantity of food are closely linked to cardiovascular (CV) diseases. Low-density lipoprotein cholesterol (LDL-C) and apolipoprotein B-containing lipoproteins are primary determinants of immune-mediated inflammation and atherosclerosis; consequently, traditional dietary recommendations emphasize low intake of animal-derived lipids and adequate amounts of fruits and vegetables and unsaturated fat.^3–6^

Over the past decade, however, a growing body of evidence from high-quality longitudinal cohort studies worldwide has prompted a re-evaluation of the diet–health relationship, shifting focus from nutritional content alone to the extent and purpose of food processing.^7–9^

Ultra-processed foods are characterized as formulations primarily derived from inexpensive industrial sources of dietary energy and nutrients combined with additives, through extensive industrial processes, resulting in poor nutritional content and the presence of cosmetic additives and neo-formed compounds, that may adversely affect health.^10,11^

The rapid transition from traditional diets to increased UPF consumption poses a challenge for the general population and patients with CV diseases or comorbidities leading to an emerging risk burden.^2,12–14^

While epidemiological evidence increasingly supports a link between UPF consumption and poor CV health, this issue remains largely underrecognized in the wider public health discussion, as well as in general cardiology.

Most national dietary guidelines continue to prioritize nutrient-/food-based recommendations disregarding the issue of food processing. As a result, UPFs are largely neglected in clinical settings, although diet remains a cornerstone of CV prevention and management.

This clinical consensus statement was developed by the ESC Council for Cardiology Practice and the European Association of Preventive Cardiology of the ESC, together with a multidisciplinary group of European experts who shared and approved the statements and the clinical advices. The aims of this document are to introduce the concept of food processing and UPFs into the knowledge base of general cardiologists, raising awareness of UPFs as a potential additional risk factor to consider as part of the patient counselling routine. Starting from a comprehensive review of the epidemiological evidence linking UPFs to CV health and its underlying mechanisms, this document provides practical approaches for incorporating UPF-related considerations into routine assessment and dietary counselling. Also, it outlines a research framework to guide future integration into clinical care while identifying and discussing key research gaps and methodological challenges that limit the translation of evidence into clinical practice.

### Chapter 1. Definition of ultra-processed foods: the Nova classification

Several systems exist to classify foods by processing level,^15^ but the Nova classification, developed by Monteiro *et al*. in 2009 and subsequently updated,^10^ has become the most widely used in epidemiological studies and policy discussions. This system helps understand that not all processed foods are the same—some are minimally altered, while others are heavily industrialized products designed to be ready-to-eat and highly palatable. Understanding these categories is important because the level of processing affects the nutritional quality of foods and their impact on health, particularly CV health.

The Nova system classifies foods and beverages into four categories, according to the extent and purpose of the industrial process they undergo, regardless of nutritional composition.^10,16^

It is noteworthy that, although providing a useful framework for categorizing foods by processing level, application of the Nova classification in long-term epidemiological studies may be affected by exposure misclassification, as food items are often classified uniformly over extended follow-up periods despite substantial changes in industrial processing practices over time. A simplified guide to Nova food classification for clinical use is provided in *Table 1*, with a comprehensive description of all Nova groups and examples available in Supplementary Material  *S1*—Supplementary data online, *Table S1*.^16^

**Table 1:** Simplified guide to Nova food classification for clinical use

Nova groups	What it means (clinician-friendly)	How to explain to patients	Examples
**Group 1: Unprocessed or minimally processed foods**	Natural or slightly altered foods, no added ingredients.	Whole foods—the way nature made them.	Fresh fruit, vegetables, eggs, plain yogurt, milk, fresh meat
**Group 2: Processed culinary ingredients**	Substances extracted from foods or nature used in cooking.	Basic cooking ingredients.	Oil, butter, salt, sugar
**Group 3: Processed foods**	Foods with added salt, sugar, or oil to make them last longer or taste better.	Still recognizable foods, but with some added ingredients.	Canned vegetables, cheese, bread, smoked meats
**Group 4: Ultra-processed foods**	Industrially made, typically with additives, little to no whole food content.	Packaged, long-shelf-life products—often high in sugar, salt, or fat.	Chips, soft drinks, sweets, processed meats, many ready meals, fruit yogurts, many breakfast cereals

### Chapter 2. Ultra-processed foods and diet quality

The poor nutrient composition of UPFs is one of the hypothesized mechanisms linking them to adverse health outcomes. On average, UPFs are higher in energy, saturated or trans fats, sugar, dietary cholesterol, and salt, while lacking essential nutrients such as vitamins, minerals, and fibre,^17,18^ while showing a negative relationship with protein, fibre, and certain micronutrients including potassium, magnesium, vitamin D, and vitamin B_12_.^19^ Furthermore, UPFs may lack bioactive compounds, which are relevant to CV health.^20–22^ Data from two US observational studies have associated increased UPF consumption with lower intake of total flavonoids^23^ and with lower urinary enterolignan concentrations.^24^

Several cohort studies worldwide have explored the relationship between UPFs and dietary scores, which assess the quality of a person’s diet based on their food choices and nutrient intake, typically promoting healthy foods (e.g. fruits, vegetables) and discouraging unhealthy ones (e.g. processed foods, excessive sugar). Among these, the Mediterranean diet has been extensively studied and is widely recognized for its positive health and cardioprotective benefits.^25,26^ It is inversely associated with UPF consumption^27–29^ but positively linked to the intake of minimally or unprocessed foods.^28^ This relationship has also been confirmed in populations of children and adolescents,^28,30^ suggesting that promoting the adoption or maintenance of a traditional Mediterranean diet could serve as an effective public health strategy to limit UPF intake and reduce the burden of nutrition-related illness, particularly cardiometabolic diseases.^31^

Importantly, the average poor nutritional composition of UPFs alone fails to fully account for their adverse health impact. Over 75 prospective studies reported that UPF consumption was associated with an increased risk of chronic diseases even after adjusting for several markers of nutritional quality such as energy, sugar, salt, saturated fats, and other key nutrients.^18,32^ This conclusion has been further corroborated by recent analyses from large European cohorts,^33^ emphasizing that non-nutritional factors (e.g. some food additives, contaminants created during processes or coming from packaging, etc.) also play a significant role in these associations.

**Figure 1:**
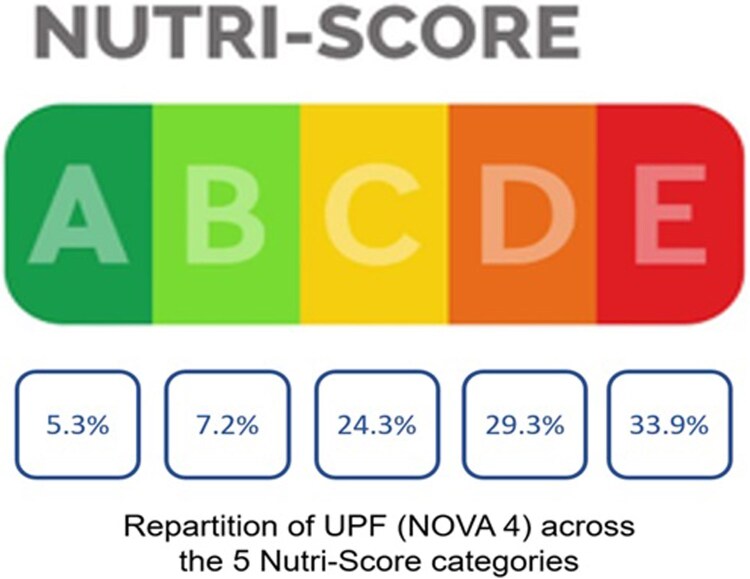
Cross-frequency between Nutri-Score and Nova classifications, OpenFoodFacts database—129 950 food products of the French market, 2024—adapted from Sarda *et al*.^36^

The distinct yet complementary nature of the ‘nutritional’ and ‘processing’ dimensions of foods is essential to understanding their health implications.^34^ For instance, a food product may possess a favourable nutrient profile (low sugar, salt content, etc.) but still be classified as ultra-processed (e.g. artificially sweetened dairy desserts, vegetable patties with emulsifiers and flavours, ‘slimming’ products such as meal-replacement shakes and powders, etc.). Conversely, minimally processed foods may have a less favourable nutrient profile (e.g. home-made cake with high sugar and butter content). The interplay between the Nutri-Score front-of-pack labelling system, which assesses the nutritional quality of food products using a colour-coded scale ranging from A (the most balanced nutritional composition) to E (the least balanced), and the Nova classification underscores the necessity of considering both dimensions for a comprehensive evaluation of food quality. Estimates suggest that over 80% of UPFs available in the Spanish market are classified as having poor nutritional value according to the Nutri-Score, although the opposite has also been found, with some UPFs receiving favourable Nutri-Score ratings.^35^ Similarly, *Figure 1* illustrates the distribution of UPFs across the different Nutri-Score categories for >120 000 products of the French market, highlighting the need for both nutritional and processing perspectives to fully assess food quality.^36^

### Chapter 3. Ultra-processed food consumption across European countries

In the last decades, population dietary patterns have shifted towards increased consumption of UPFs.^37,38^ For instance, in Spain, UPF purchase nearly tripled between 1990 and 2010.^39^

According to data from Euromonitor on global trends in UPF product sales, *per capita* annual volume in 2016 was highest in the Netherlands (144 kg), Germany (142 kg), and the UK (141 kg), while Eastern Europe reported the lowest volume sales.^40^ The main contributors to solid and liquid UPF purchase in Europe were bakery products and carbonated drinks, respectively.^40^

A systematic review showed the Netherlands and UK had the highest energy intake from UPF (61% and 54%, respectively), and Southern Europe the lowest, such as Spain (25%), Portugal (22%), and Italy (18%), presented the lowest percentages.^41^ Higher UPF consumption was associated with younger age, urban residence, and unmarried status. Associations with education, income, and socioeconomic status varied by country.^41^ This is confirmed by the data from household availability sources, which revealed highest UPF availability in the UK (51%), Germany (46%), and Ireland (46%) and lowest in Portugal (10%), Italy (13%), or Greece (14%).^42^ Also, Mertens *et al*.^43^ reported that UPFs accounted for an average of 27.6% of total energy intake in 22 countries, highest in Germany (39%) and Sweden (44%), lowest in Romania (16%) and Hungary (17%). Again, bakery products and soft drinks accounted for the majority of UPF intake.

### Chapter 4. Ultra-processed foods and cardiovascular health: conceptual framework and evidence approach

The relationship between UPF consumption and CV health is complex and multifaceted. Within the framework adopted in this clinical consensus statement, UPFs are not considered as acting in isolation, but as part of a causal chain in which dietary exposure contributes to the development of intermediate cardiometabolic risk factors, such as obesity, hypertension, dyslipidaemia, and insulin resistance, which in turn mediate CV outcomes. Accordingly, associations between UPF intake and clinical CV endpoints should be interpreted primarily as indirect effects mediated through established risk factors, rather than as evidence of a direct causal relationship (*Graphical Abstract*).

In this clinical consensus statement, we therefore adopted a structured approach to examine the evidence, first focusing on the associations between UPFs and key cardiometabolic risk factors, followed by the impact of UPFs on CV disease incidence and progression. While available evidence mostly relies on observational data, therefore limiting definitive causal inference, the consistency of associations across multiple intermediate risk factors and outcomes, together with supportive mechanistic evidence, largely derived from studies on typical components of UPFs (e.g. cosmetic food additives), provides biological plausibility for these pathways.

The evidence discussed in Chapters 4.1 and 4.2 is derived from a systematic review conducted in accordance with the Preferred Reporting Items for Systematic Reviews and Meta-Analyses (PRISMA) guidelines. The review examined the influence of UPF consumption on intermediate CV risk factors and then on CV clinical endpoints, based on studies identified through comprehensive searches across four databases (PubMed, MEDLINE, Embase, and Scopus) up to 30 July 2025. Eligibility was restricted to longitudinal studies and randomized controlled trials (RCTs) involving adults, with UPF exposure classified according to the Nova system. Quality assessment was performed using the National Heart, Lung and Blood Institute [National Institutes of Health (NIH)] Quality Assessment Tools. The clinical consensus statements regarding the association between UPF and both CV risk factors and outcomes were shared and approved by the task force of consensus experts.

Full details of the methodology, including the search strategy, inclusion/exclusion criteria, and grading process, are available in the Supplementary material  *S2*—Systematic review, including Supplementary data online, *Tables S2*–5 and Supplementary data online, *Figure S1*.

### Chapter 4.1. Ultra-processed foods and cardiovascular risk factors

#### Obesity

Large observational studies conducted in different populations^44–49^ consistently reported direct associations between UPF consumption and risk of overweight and obesity.

Higher UPF consumption was consistently associated with increased risk of overweight and obesity, with hazard and odds ratios ranging from 1.09 to 1.61. Comparing high vs. low intake, risks increased by 15%–16%, while each 10% UPF increase was linked to 9%–18% higher odds. Consuming >50 g/day of UPFs raised the odds of overweight and obesity by 34%–45% and abdominal obesity risk by up to 61%.

The mechanisms linking UPFs to weight gain include increased energy density, reduced satiety, altered eating behaviour (e.g. faster eating, reduced mastication), and possible effects on gut–brain signalling.^50^

Summary of clinical evidence: A consistent direct association was found between UPF consumption and the risk of developing obesity or overweight. All six studies^44–49^ reported a positive link, indicating that higher UPF intake is associated with a greater risk of becoming obese/overweight. All studies were rated as ‘Good’ quality, strengthening the reliability of this evidence.

Additionally, three RCTs^51–53^ reported a direct association between UPF intake and increased adiposity. Despite small sample sizes (9–55 participants), two were rated ‘Good’ and one ‘Fair’ quality, reinforcing observational evidence with experimental data. For further details, see Supplementary material.

#### Type 2 diabetes

Among 10 prospective cohort studies,^54–63^ nine examined the association between UPF consumption and the risk of type 2 diabetes (T2D), while one assessed the risk of prediabetes.^63^ Type 2 diabetes was consistently defined across studies using standard criteria: fasting plasma glucose ≥7.0 mmol/L, 2 h post-load glucose ≥11.1 mmol/L, or glycated haemoglobin (HbA1c) ≥ 48 mmol/mol. Prediabetes was defined by impaired fasting glucose or glucose tolerance.^62^

Across these studies, higher UPF intake was consistently associated with increased risk of T2D and prediabetes. The hazard ratios (HRs) for T2D ranged from 1.13 (95% confidence interval [CI] 1.03–1.23) to 1.80 (95% CI 1.47–2.20) comparing the highest to lowest consumption categories. Similarly, prediabetes risk was raised by 24% (HR 1.24; 95% CI 1.04–1.49) among individuals with the highest UPF intake.

Potential mechanisms linking UPFs to T2D include high content of added sugars that may dysregulate the hepatic metabolism of fructose and promote hepatic and whole-body insulin resistance.^64^ In addition, UPFs also tend to be low in fibre, and it is well established that high-fibre diets are protective against elevated HbA1c, fasting plasma glucose levels, and postprandial glucose spikes.^65^

Moreover, UPFs contain numerous chemical additives, some of which may act as endocrine disruptors, potentially contributing to an increased diabetes risk.^66^

Summary of clinical evidence: A consistent direct association was found between UPF consumption and the risk of developing T2D. All nine studies^54–63^ reported a positive association, and all were rated as ‘Good’ quality, supporting the robustness of this evidence.

#### Hypertension

Five prospective cohort studies^67–71^ examined the link between UPF intake and hypertension. Three reported positive associations, with identified risk estimates ranging from 1.20 to 1.35. One study^70^ found higher odds among African American and Caucasian participants in the highest UPF intake group compared with the lowest, though the association was not statistically significant for African Americans.

UPFs are typically high in sodium, added sugars, and unhealthy fats, leading to elevated blood pressure.^72^ Their energy-dense, nutrient-poor nature promotes overeating and weight gain, with obesity increasing hypertension risk through cardiac and hormonal changes.^72^ UPFs may also trigger chronic inflammation and oxidative stress, leading to vascular dysfunction,^73^ and emerging evidence suggests UPFs disrupt gut microbiota, potentially affecting blood pressure regulation.^74^

Summary of clinical evidence: Out of five studies,^67–71^ four^67,68,70,71^ reported a direct association, while one^69^ found no association. Quality ratings ranged from Fair to Good, suggesting moderate evidence supporting a link between UPF intake and elevated blood pressure.

#### Dyslipidaemia

Two prospective cohort studies examined UPF intake and dyslipidaemia risk. One, from the ELSA-Brasil cohort,^75^ assessed various lipid abnormalities, while the other, on older adults, focused on hypertriglyceridaemia, low high-density lipoprotein cholesterol (HDL-C), and LDL-C.^76^ Both reported increased dyslipidaemia risk with higher UPF consumption despite differing populations and outcomes. Isolated hypertriglyceridaemia was elevated by 30% in ELSA-Brasil and over two-fold in the older cohort; low HDL-C risk increased by 18% and more than two-fold, respectively.^75,76^ While these findings support a potential link between UPF intake and dyslipidaemia, the evidence is limited to two studies with relatively modest sample sizes.

Mechanisms linking UPF intake to dyslipidaemia possibly include the high content of trans and saturated fats in these foods, which disrupt lipid metabolism and promote atherogenic profiles.^77^ Trans fats raise LDL-C, lower HDL-C, impair lipid-processing enzymes, and increase the proportion of small, dense LDL particles.^78^ Saturated fats similarly elevate LDL-C by boosting hepatic cholesterol synthesis and reducing LDL clearance.^79^ These changes promote systemic inflammation, endothelial dysfunction, and foam cell formation, thereby accelerating atherosclerosis and linking UPF consumption to dyslipidaemia and CV disease.^80^

Summary of clinical evidence: Both included studies^75,76^ showed a direct association between UPFs and abnormal lipid profiles, with good quality ratings, providing initial yet reliable evidence.

#### Metabolic syndrome

Metabolic syndrome (MetS) is a pathophysiological state characterized by a cluster of at least three CV risk factors, including abdominal obesity, insulin resistance, elevated blood pressure, and dyslipidaemia.^81^ Dietary factors have long been recognized as crucial in the development of MetS with past research focusing on the intake of specific foods or nutrients. However, the relationship between different degrees of food processing and MetS has received less attention.

Two prospective cohort studies examined the association between UPF intake and MetS risk.^82,83^ One study^82^ reported a significant association (HR 1.17; 95% CI 1.01–1.35), whereas the other, a smaller study,^83^ found no association.

The mechanisms linking UPFs to MetS extend beyond their poor nutritional quality and in part overlap with the mechanisms relating UPFs and dyslipidemias. Additives such as artificial sweeteners may disrupt gut microbiota and glucose metabolism.^84^ Additionally, industrial processing can increase glycaemic load and impair gut–brain satiety signalling, leading to increased secretion of incretin hormones and gastric inhibitory polypeptide, which promote insulin secretion, appetite stimulation, and food overconsumption.^85^

Summary of clinical evidence: Although data on dyslipidaemia (see previous subchapter) evidence an involvement of insulin-resistance mechanisms—key factor in MetS—evidence on the association between UPF consumption and MetS is limited and inconclusive, with findings varying across the available studies.

#### Non-alcoholic fatty liver disease

Non-alcoholic fatty liver disease (NAFLD), recently termed metabolic dysfunction-associated steatotic liver disease (MASLD), is the most prevalent chronic liver condition globally, with a prevalence of 32.4% overall and higher rates in individuals with obesity (75.3%) or T2D (55.5%).^86–88^

Four prospective cohort studies investigated the association between UPF consumption and risk of NAFLD,^89–93^ including one nested within the PREDIMED-Plus RCT.^91^ Two^89,90^ examined overall NAFLD risk, one^92^ focused on severe NAFLD, and another^91^ assessed NAFLD-related biomarkers, including hepatic steatosis index (HSI) and fatty liver index (FLI).

Higher UPF consumption was consistently associated with increased NAFLD risk, with HRs ranging from 1.26 to 1.48,^88,89,91^ and was linked to elevated FLI (β & 1.60; 95% CI 1.24–1.96) and HSI (β & 0.43; 95% CI .29–.57) scores.^91^

UPFs may contribute to MASLD through high fructose and saturated fat content, additives, and low fibre, promoting fat accumulation, oxidative stress, inflammation, and gut microbiota disruption.^93–96^

Summary of clinical evidence: A consistent direct association was found between UPF consumption and NAFLD. All five studies^89–93^ reported a positive link. All were rated as ‘Good’ quality, indicating strong and consistent evidence across various populations.

#### Chronic kidney disease

Chronic kidney disease (CKD) is a major risk factor for CV disease and is commonly associated with other conditions such as diabetes and hypertension.^97^

The association between UPF with CKD risk has been examined in five prospective cohort studies.^98–102^ Two studies^98,99^ defined CKD based on a decline in estimated glomerular filtration rate, while three studies^100–102^ used broader outcomes such as CKD stage ≥3, CKD-related death, or kidney failure requiring dialysis or transplant.

Higher UPF intake was associated with increased CKD risk in all studies. One study in older adults reported a 74% higher risk of renal function decline (OR 1.74; 95% CI 1.14–2.66).^101^ Other studies found risk estimates ranging from OR 1.11 (95% CI 1.06–1.17)^99^ to HRs between 1.04 (95% CI 1.03–1.06)^100^ and 1.58 (95% CI 1.07–2.34).^102^

The pathophysiological mechanisms likely involve chronic inflammation, oxidative stress, dysregulated lipid metabolism, insulin resistance, immune dysfunction, and gut microbiota disruption.^103^ Animal studies suggest that advanced glycation end products from UPFs activate the complement pathway and impair intestinal barrier permeability, contributing to kidney damage.^104^

Summary of clinical evidence: A consistent direct association was found between UPF consumption and the risk of developing CKD. All five studies^98–102^ reported a positive association. All studies were rated as ‘Good’ quality, indicating strong and coherent evidence.

#### Ultra-processed foods and cardiovascular risk factors: clinical consensus statement

From the available evidence, higher UPF consumption is associated with CV risk factors. In particular, evidence from cohort studies and RCTs consistently reports associations between higher UPF consumption and increased body weight, T2D, and dyslipidaemia.

Ultra-processed food intake is associated with T2D risk among adults, while there was limited evidence available in relation to the risk of prediabetes.

Evidence on the association between UPF and hypertension derives from relatively small sample-sized studies (hypertensive cases, N = 370–4329), requiring additional higher sample studies.

Although based on small sample-sized studies, available evidence suggests an association between UPF intake and high hypertriglyceridaemia and low HDL among adults. Evidence on MetS is scarce, limited by small samples, and inconclusive.

Based on current evidence, UPF intake is associated with NAFLD, particularly in individuals with elevated FLI, intrahepatic fat, or HSI >36, as well as with renal function decline.

Supplementary data online, *Table S4* (in Supplementary Material  *S2*—Systematic review) presents the direction of associations identified in studies on UPF consumption and CV risk factor, together with NIH Quality Assessment ratings.

These associations are derived predominantly from observational studies and should be interpreted with caution, as residual confounding related to socioeconomic and lifestyle factors, as well as reverse causation, cannot be fully excluded. Moreover, the evidence base includes only a limited contribution from RCTs, which remain scarce, short term, and largely focused on surrogate endpoints.

### Chapter 4.2. Ultra-processed foods and cardiovascular clinical endpoints

#### Arrhythmias

A high consumption of UPFs, with low potassium amount, combined with a decreased intake of vegetables, may possibly lead to a significant reduction in potassium intake, thereby increasing the risk of arrhythmias, particularly ventricular arrhythmias in patients with left ventricular dysfunction.^105–107^

Also, due to their contribution to the development of hypertension, obesity, diabetes, and other risk factors, UPF consumption may indirectly favour the occurrence of arrhythmias. However, studies specifically exploring the relationship between UPF consumption and arrhythmias remain scarce.

To date, only one prospective study has reported on the risk of atrial fibrillation (AF) in relation to UPFs.^107^ Using data from the UK Biobank, the authors examined 121 300 individuals (mean age 59.4 ± 7.8 years, 56.4% female) with 4579 incident AF cases over a median follow-up of 8.8 years and found a 5% increase in AF risk for every 10% increase in the absolute UPF intake. Also, participants in the highest category of UPF consumption reported a 13% increased risk of AF (HR 1.13; 95% CI 1.02–1.24) compared with those in the lowest category, even after adjusting for key confounders.

Summary of clinical evidence: One large prospective study,^107^ rated as ‘Good’ quality, reported a direct association between UPF consumption and the risk of AF, providing initial evidence supporting this association, but more data are needed to confirm the relationship.

#### Heart failure

Similarly to arrhythmias, risk factors for heart failure (HF), such as hypertension, obesity, and diabetes, are linked to the consumption of UPFs. However, there is limited evidence on the direct relationship between UPFs and HF. Two prospective studies have explored the association between UPF consumption and composite CV outcomes, including HF. In the Framingham Offspring Study, which involved 3003 adults free from CV disease, each additional daily serving of UPFs was associated with a 5% (95% CI 1.02–1.08) increased risk of overall CV disease, including congestive HF.^108^ Additionally, a longitudinal study from the Prospective Urban and Rural Epidemiology (PURE) study, which analysed data on over 130 000 participants from five continents, found no link between UPFs and HF (HR 1.04; 95% CI .98–1.10 per one serving increase).^109^ Analyses from the Chronic Renal Insufficiency Cohort study including patients with CKD did not find any significant association between UPFs and the incidence of CV disease, including HF.^98^

Summary of clinical evidence: There is inconsistent evidence regarding the link between UPF consumption and HF. Among three studies,^98,108,109^ only one^108^ found a direct association, while the others^98,109^ reported no association. All studies were rated as ‘Good’ quality: evidence for this outcome remains limited and conflicting.

#### Incidence of cardiovascular disease

The relationship of UPFs with primary incidence of CV disease has been investigated by three large prospective studies,^110–112^ as well as in two smaller cohort based in the USA and Iran, respectively.^107,113^ In the Atherosclerosis Risk in Communities (ARIC) study on 13 548 adults aged 45–65 years,^110^ participants in the highest compared with lowest quartile of UPF intake had a 19% higher risk of coronary artery disease (HR 1.19; 95% CI 1.05–1.35) after adjusting for sociodemographic factors and health behaviours. In the large NutriNet-Santè cohort in France using data on 105 159 adults,^111^ each 10% increase in UPF intake was linked to a 12% higher risk of CV disease (95% CI 1.05–1.20) and specifically 13% higher risk of coronary heart disease (HR 1.13; 95% CI 1.02–1.24) and 11% increase in the risk of developing cerebrovascular disease risk (HR 1.11; 95% CI 1.01–1.21). These associations remained significant after controlling for dietary quality and other potential confounders.

Longitudinal data from three large US prospective cohorts^112^ (i.e. Nurses’ Health Study, *n* & 75 735; Nurses’ Health Study II, *n* & 90 813; and Health Professionals Follow-Up, *n* & 40 409) further corroborated previous observational evidence by showing that highest UPF consumption (as compared with the lowest category) was associated with increased hazards of CV disease (HR 1.11; 95% CI 1.06–1.16) and specifically coronary heart disease (HR 1.16; 95% CI 1.09–1.24), while the association with stroke incident was not significant (HR 1.04; 95% CI .96–1.12).

In the Framingham Offspring cohort (n & 3003 adults),^107^ each additional daily serving of UPFs was associated with a 7% (95% CI 1.03–1.12), 9% (95% CI 1.04–1.15), and 5% (95% CI 1.02–1.08) increase in the risk of hard CV disease, hard coronary heart disease, and overall CV disease, respectively.

Lastly, in a small cohort of Iranian adults (n = 2050),^113^ participants reporting the highest intake of UPF had a 68% greater incidence of CV disease compared with those with the lowest intake (HR 1.68, 95% CI 1.14–2.48).

Several mechanisms unrelated to the poor nutritional composition of these foods have been identified, including factors pertaining to food structure, additives, and processing-derived compounds that may influence metabolic and CV health.^114^

Biological mechanisms implicated include changes in lipid metabolism, dysbiosis, excess adiposity, systemic inflammation, oxidative stress, impaired glucose regulation, and elevated blood pressure.^114^

Based on the multiple studies included in the systematic review, in subjects without known CV diseases, the incidence of CV disease was consistently associated with UPF consumption.

In relation to recurrent CV disease, one prospective cohort from Italy^115^ included participants with a history of CV disease and suggested that high UPF intake was associated with higher risk of CV disease mortality (HR 1.65; 95% CI 1.07–2.55). A linear dose–response relationship of 1% increment in UPF intake with all-cause and CV mortality was also observed. The study also provided an insight into potential mechanisms and reported that altered renal function explained 18.3% and 16.6% of the relation between UPF with all-cause and CV mortality, respectively.

Summary of clinical evidence: A consistent direct association was found between UPF consumption and the risk of incident CV disease. All five studies^107,110–113^ reported a positive association. Studies were rated as ‘Good’ or ‘Fair’ quality, indicating strong and consistent evidence overall. In patients with a history of CV disease, one study showed that high UPF intake was associated with higher risk of recurrent CV disease.

#### Cardiovascular mortality

Thirteen prospective cohort studies have investigated the association between UPF consumption and the risk of CV mortality.^108,109,115–125^ Among these, four studies reported no significant association between higher UPF intake and CV mortality risk^119,121–123^; however, these studies were often limited by modest sample sizes and potential confounding biases.

Conversely, nine studies^108,109,115–118,120,124,125^—of which one cohort^115^ was focused on a subgroup of a previously evaluated cohort^116^—including large cohort analyses and pooled data, consistently found that higher UPF consumption was associated with an increased risk of CV mortality. The relative risk estimates ranged from 9% (HR 1.09; 95% CI 1.02–1.16) up to 65% increased risk (HR 1.65; 95% CI 1.07–2.55) in those with the highest UPF intake.^109,116,120,124,125^ Dietary assessments in these studies were primarily based on semiquantitative food frequency questionnaires (FFQs) or 24 h dietary recalls, which allowed extraction and classification of UPF consumption.

While all studies were prospective in design, the stronger evidence comes from large sample-sized cohorts^116,120,124^ and pooled analyses,^109,125^ reinforcing a likely positive association between UPF intake and risk of CV mortality.

**Figure 2:**
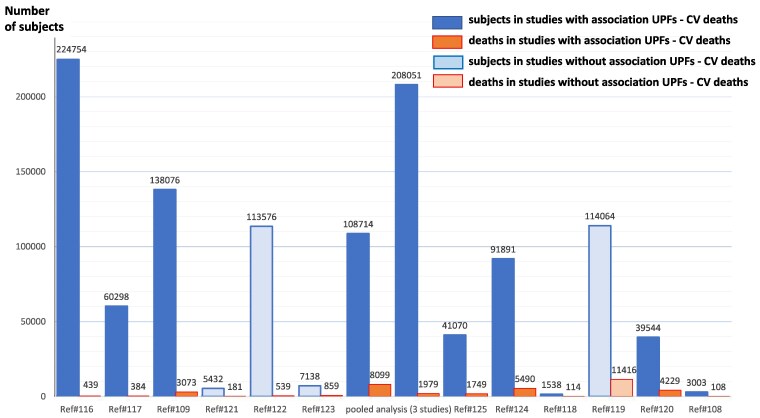
Prospective studies examining the association between ultra-processed foods (UPFs) and cardiovascular (CV) mortality. Bars indicate the number of participants included in each study (precisely shown by the numbers above the bars). Dark blue bars indicate the number of participants in studies reporting a significant association with CV death, with corresponding CV death counts shown in orange. Pale blue bars represent studies reporting no significant association, with CV death counts shown in pale orange. Reference numbers are provided below each two-column indicating the number of participants, blue, and the number of deaths, orange, reported for each study

Summary of clinical evidence: A mostly consistent direct association was found between UPF consumption and CV disease mortality. Nine of 13 studies,^108,109,115–118,120,124,125^ with sample sizes up to 357 000, reported a positive association. Four studies^119,121–123^ reported no association. All were rated as ‘Good’ quality, suggesting predominantly supportive evidence despite some variability (*Figure 2*).

#### Ultra-processed foods and cardiovascular clinical endpoints: clinical consensus statement

Based on the studies included in the systematic review, UPF consumption is associated with CV morbidity and mortality. Regarding the relationship between UPF and risk of AF, evidence remains scarce, and additional studies are needed to explore this outcome.

Available studies suggest that UPF intake is associated with risk of HF; however, more consistent evidence is required.

High UPF intake is linked to higher CV mortality among individuals with preexisting CV disease; however, this finding is based on a single longitudinal study; therefore, the evidence remains limited.

In the systematic review, UPF intake is associated with CV disease mortality in the majority of the studies investigating this relationship (*Figure 2*).

Supplementary data online, *Table S4* (in Supplementary Material  *S2*—Systematic review) presents the direction of associations identified in the studies on UPF consumption and CV clinical endpoints, along with NIH Quality Assessment of the relative studies.

Given that the available evidence is predominantly derived from observational studies, these associations should be interpreted with caution, as residual confounding and reverse causation cannot be fully excluded. To date, RCTs assessing the impact of UPF reduction on hard CV clinical endpoints are lacking, with existing interventional evidence limited to short-term studies focusing on intermediate or surrogate outcomes.

### Chapter 5. How food processing possibly impacts cardiovascular health

Beyond their unfavourable nutritional profile, UPFs may affect CV health through mechanisms specifically related to extensive industrial processing. The pathways outlined below are mainly based on experimental, short-term, or component-specific studies and should be viewed as supporting biological plausibility rather than demonstrating direct causal effects on CV outcomes.

Diets rich in added sugars, trans fats, and saturated fats, largely present in many UPFs, are known to contribute to the development of atherosclerosis, endothelial dysfunction, and T2D.^126,127^ Additionally, UPFs often displace whole, minimally processed foods rich in fibre, polyphenols, and micronutrients, thereby reducing intake of cardioprotective compounds.

As said, UPFs may also exert harmful effects through non-nutrient pathways. Although the exact mechanisms remain to be elucidated, potential contributors include food additives, processing-related contaminants, and structural alterations of food matrices.

Certain industrial processing techniques can lead to the formation of compounds such as advanced glycation end products, acrylamide, and industrial trans fatty acids. In addition, the long shelf life of many UPFs, as a marker of intensive processing and packaging, may be associated with migration of packaging-related contaminants (e.g. bisphenols, phthalates, mineral oils, microplastics) that could have carcinogenic effects and increase the risk of CV disease, obesity, insulin resistance, and T2D.^128–130^

Ultra-processed foods also commonly contain cosmetic additives (e.g. sweeteners, emulsifiers, thickeners, colourants). Experimental studies and limited human evidence indicate that some additives may affect gut microbiota composition and inflammatory pathways and, in specific contexts, markers of DNA damage, with potential downstream metabolic effects.^131–140^

Processing-related changes in food matrix may also influence satiety, eating behaviour, glycaemic responses, and nutrient bioavailability. Highly processed foods often lack the natural cellular structure of whole foods, leading to faster digestion and absorption, which may favour higher energy intake and reduce the delivery of fermentable substrates to the gut microbiota.^141–143^

Finally, UPFs are heavily marketed, with packaging designed to appeal through vibrant imagery, animal and cartoon characters, and health-related claims.

While the impact of packaging and marketing on consumption is not fully understood, it is likely that such strategies encourage overconsumption.^144^ Comparisons between UPFs and addictive substances remain controversial and should be interpreted with caution in clinical contexts.^145^

### Chapter 6. From clinical awareness to political strategies: strategic and policy-level considerations

Strategic and policy-level approaches can help shift population-wide exposure to UPFs to healthier dietary patterns.^18^ These measures include consumer education, food labelling, food system regulation, and public health governance, all serving as valuable tools to create environments that can support healthy food choices and dietary change.^136,146–154^ Understanding these broader strategies can support clinicians to better contextualize patient behaviours, anticipate challenges, and enhance the effectiveness of dietary counselling. These policy-level strategies are presented to support clinicians in contextualizing patient behaviours and reinforcing dietary counselling, rather than as prescriptive regulatory recommendations.

*Table 2* illustrates how these broader actions translate into practical insights and clinical takeaways, bridging strategic context to everyday cardiology practice. For a more detailed discussion of these strategic and policy-level considerations, see Supplementary Material  *S3*.

**Table 2:** Clinical takeaways from strategic context and policy implications for the general cardiologist

Strategic area	Bridge for cardiologists	Clinical takeaway
**Consumer-facing policies: education, empowerment, labelling**	Patients are increasingly exposed to nutrition messages and labelling tools; clinicians need to interpret these accurately.	Stay updated on food labelling initiatives and public education to better counsel patients on identifying and reducing ultra-processed foods.
**System-level and macroeconomic strategies**	Structural barriers (e.g. marketing, availability) influence food choices; clinicians should account for this in counselling.	Understand how food marketing and availability affect patient choices; tailor advice to overcome environmental barriers.
**Aligning dietary health with environmental sustainability**	Some patients are motivated by sustainability concerns, which can reinforce dietary change.	Highlight the cardiovascular and environmental benefits of reducing ultra-processed foods to motivate patient behaviour change.
**Safeguarding policy integrity and incentivizing innovation**	Industry influence and reformulation trends may confuse patients about what is truly ‘healthy’.	Encourage patients to choose minimally processed food alternatives and support ongoing innovations in healthier foods.

### Chapter 7. Clinical counselling framework on ultra-processed foods for general cardiologists *(implementation focus)*

This section provides practical guidance for general cardiologists to integrate UPF-focused dietary counselling into clinical care without adding substantial burden to routine cardiology visits. These clinical consensus statement advices have been conceived to help clinicians assess, communicate, and implement patient-specific advices in real-world settings.

In routine clinical practice, UPF counselling can be implemented using a stepwise framework, beginning with brief screening of UPF intake, followed by patient-tailored communication, practical food substitutions, and reinforcement over time.

General cardiologists should recognize UPFs as an emerging and important dietary risk factor. While lifestyle interventions, including diet, along with the evaluation of traditional haemodynamic, anthropometric, and metabolic parameters, are routinely part of clinical visits—particularly in preventive cardiology—few cardiologists currently include specific recommendations on UPF consumption during dietary counselling. Integrating UPF knowledge into patient care and medical training programmes can foster preventive dietary measures integrated into a broader nutritional approach that overcomes the limitations of traditional reductionist models focusing solely on isolated nutrients.

Particularly in outpatient clinics and whenever the general cardiologists evaluate lifestyle and potential lifestyle interventions, we advise implementing targeted evaluation and counselling on UPFs both in individuals assessed for CV and cardiometabolic risk factors and in patients with established CV and cardiometabolic overt diseases.

Importantly, UPF counselling should not replace or delay the management of established CV risk factors but rather be integrated as a complementary component within routine lifestyle assessment.

Given time and resource constraints during cardiology visits, this approach is intended to be brief and embedded within routine lifestyle assessment, rather than delivered as a standalone intervention.

In addition to standard medical history questions regarding lifestyle and eating habits, clinicians are advised to explicitly assess the frequency and quantity of UPF consumption. Most of patients, and even healthcare professionals, are unaware that foods marketed as ‘healthier’ and ‘slimming’ options often belong to the UPF category. For clearer patient communication, we advise referring to a simplified format (*Table 1*) of the Nova classification (see Supplementary data online, *Table S1*  in Supplementary material  *S1*) to help with the delivery of practical dietary advice. For practical utility, we propose the following clinical consensus statement advices, included in the accompanying Box (do’s and don’ts) for quick reference during patient consultations.

BOX Clinical consensus statements—practical do’s and don’ts for general cardiologists on counselling patients about UPF consumption

**Table:** 

Do’s	Don’ts
**Setting** **Apply UPF counselling in outpatient clinics and during routine lifestyle assessment, particularly in preventive cardiology**	Do not prioritize lifestyle counselling in acute settings or highly specialized consultations
**Implement targeted evaluation and counselling on UPFs particularly in individuals assessed for cardiovascular and cardiometabolic risk factors and in patients with established cardiovascular or cardiometabolic overt diseases**	Do not omit UPF evaluation when providing dietary advice as part of routine care
**During dietary assessment, routinely screen for UPF intake:** **—Frequency** **—Quantity**	Avoid neglecting UPF evaluation in routine patient visits
**Support patient understanding using visual aids to clearly identify UPFs**	Do not rely exclusively on verbal explanations without supportive materials
**Communicate risks clearly using simple, actionable language**	Do not overwhelm patients with complex nutrition jargon
**Provide practical and achievable dietary substitutions (e.g. plain instead of flavoured or sweetened yogurt, water or unsweetened beverages instead of sugary drinks)**	Do not focus solely on calorie counting or isolated nutrients
**Tailor advice to individual clinical status, preferences, and readiness to change**	Don’t give one-size-fits-all advice or disregard the patient’s background and needs
**Reinforce benefits of whole and minimally processed foods**	Do not emphasize UPF avoidance without promoting healthier whole food alternatives
**Promote reading nutrition labels and ingredient lists**	Do not rely only on front-of-pack claims (e.g. ‘low sugar’, ‘low fat’) without considering the number and nature of ingredients
**Advise behaviours known to reduce the use of UPFs:** -**Prioritize of home cooking** -**Focus on specific high-risk UPF groups (e.g. sugary drinks, packaged snacks, processed meats)** -**Promote fibre-rich, textured foods and slower eating** -**Address the timing and context of meals** -**Support behaviour change through personalized counselling**	Do not omit behavioural strategies when providing dietary advice on UPFs
**Integrate quick, focused questions and simple advices into existing lifestyle discussions**	Do not allow UPF counselling to unnecessarily prolong or complicate consultations
**Where appropriate, support policies that improve food environments (e.g. labelling, marketing restrictions, access to healthy foods)**	Do not ignore the role of policy and food environments in shaping patient behaviour

- **a. Routinely screen for UPF intake**

Consider to incorporate assessment of patients’ UPF consumption as a standard part of dietary history-taking. When feasible, use brief, validated tools, as the Nova-UPF screener,^155^ to ensure accurate and consistent evaluation. For example, the NOVA-UPF screener is a brief checklist-based tool that assesses UPF exposure across predefined food subcategories and provides a simple summary score, enabling rapid identification of higher UPF intake without complex dietary assessments.

- **b. Communicate clearly and effectively**

Provide clear, evidence-based information about the CV risks associated with diets high in UPFs. Avoid technical jargon while focusing on delivering concise, actionable advice that can be easily integrated into patient counselling.

- **c. Support patient understanding with visual aids**

Use visual tools. Show UPFs as photos/icons of how they appear for sale in stores to help patients better understand and remember key messages about UPF reduction.

- **d. Encourage practical and feasible dietary changes**

Suggest realistic substitutions, such as recommending patients replace sugary drinks with water or unsweetened beverages and choose whole or minimally processed foods as alternatives to ultra-processed options.

- **e. Tailor counselling to patient needs**

Counselling should be tailored to each patient’s individual needs, clinical status, and readiness to change. The intensity and focus of UPF counselling should differ according to clinical context. In general prevention, focus on raising awareness and supporting gradual, sustainable dietary improvements. For secondary prevention or patients at high CV risk, place greater emphasis on reducing UPF intake as part of a broader risk reduction strategy. In all cases, consider patient preferences, cultural background, and any potential barriers, so that the advice is accurate and personalized.

- **f. Reinforce benefits of whole and minimally processed foods**

Emphasize the CV and overall health benefits of consuming whole and minimally processed foods as preferable alternatives to ultra-processed options.

- **g. Promote reading nutrition labels and ingredient lists**

Advise patients to carefully read nutrition labels and ingredient lists. As a practical rule, if a product contains more than five ingredients, especially with unfamiliar or artificial additives, it is likely an UPF. Encourage choosing similar foods with fewer ingredients and simpler compositions. As an example, advise to replace fruit-flavoured yogurts for plain yogurt.

- **h. Use evidence-based behavioural strategies to reduce UPFs**

Individual-level strategies target both the quality of the diet and the behavioural context of food choices. These strategies include the following actions that have been shown to reduce the UPF consumption and constitute part of the behavioural advices.^156–169^ Further details are provided in **Supplementary Material**  *S3*.

- **1. Prioritizing home cooking and meal planning**^156–160^

*Practical advices:* Cardiologists can encourage patients to cook at home more frequently by asking about current cooking habits and providing simple meal-planning resources or referrals to nutrition services.

- **2. Focusing on specific food groups**^119,161^

*Practical advices:* Begin dietary counselling by targeting reduction of high-risk UPFs such as sugar-sweetened beverages, packaged snacks, and processed meats to provide clear, manageable goals for patients.

- **3. Promoting fibre-rich, textured foods and slower eating**^162,163^

*Practical advices:* Advise patients to prefer high-fibre, minimally processed foods and practice slower, mindful eating to enhance satiety and reduce overeating of UPFs.

- **4. Addressing the timing and context of meals**^164–169^

*Practical advices:* Discuss with patients the potential benefits of avoiding late eating and establishing regular meal patterns to support overall diet quality and reduce UPF intake.

- **5. Supporting behavioural changes through personalized counselling**

*Practical advices:* Refer to behavioural techniques like motivational interviewing and goal-setting tailored to individual cultural and socioeconomic contexts to improve patient adherence to dietary changes, if a specific outpatient clinic on nutrition is available.

- **i. Keep it practical within clinic time**

Cardiology visits are usually short and full of important topics to cover. To add UPF counselling without making appointments longer, use quick screening questions and focus on simple, clear advice that patients can easily follow. More detailed nutrition guidance can be left to dietitians or given through handouts and visuals. Although formal cost-effectiveness analyses are currently lacking, brief UPF counselling is likely to be a low-cost and scalable intervention when integrated into existing lifestyle and prevention strategies.

- **j. Interprofessional collaboration**

If dietary issues are present, if feasible, interprofessional collaboration may include dietitians, nutritionists, physiotherapists, and psychologists; the subjects may be referred to dietary service, registered dietician/nutritionist, for expert nutritional management, communicating the treatment targets and a tentative timeline to achieve them.

### Chapter 8. Research gaps, key challenges, and potential solutions for future studies

Despite growing epidemiological evidence linking UPF consumption to adverse CV outcomes, several critical research gaps and challenges still remain. Addressing these is crucial to strengthen the evidence base, refine clinical guidelines, and inform effective public health policies.

#### Research gaps

Future research should prioritize large longitudinal studies in diverse population settings in order to address the long-term impact of UPF consumption on CV health. Such studies can provide critical insights into causal relationships, help inform public health recommendations, and provide robust evidence for future clinical guidelines.

In parallel, mechanistic studies are required to clarify the biological pathways through which UPFs may contribute to CV disease. Although current human studies have not yet established the exact mechanisms linking UPF intake to health outcomes,^50,170^ proposed mechanisms can be grouped into three broad categories: (1) food choice mechanisms, including hyper-palatability, low cost, long shelf life, and packaging that encourages overconsumption; (2) food composition mechanisms, related to the nutritional profile, such as added sugars, fats, salt, energy density, food texture, and use of additives and low-calorie sweeteners; and (3) digestive and physiological processes, such as reduced oral processing effort, faster eating rate, altered gastric emptying and gastrointestinal transit times, and disruption of the gut microbiome.

Finally, social and environmental determinants that drive UPF consumption and health inequities need more in-depth investigation and should be considered in delivering dietary advices to patients.

The food environment, which includes aspects such as cost, availability, marketing, labelling, packaging, and access to cooking facilities, plays a key role in shaping dietary behaviours, often promoting the selection of UPFs in replacement of minimally processed alternatives, particularly in disadvantaged communities.^170^

#### Key challenges and potential solutions

The UPF category is broad and heterogeneous, and this renders it difficult to isolate causal factors or standardize measurement across studies. Also, exposure misclassification is a relevant concern, as food items are often classified uniformly across long follow-up periods despite changes in industrial processing.

Validated and accurate tools for assessing UPF intake remain under development, limiting comparability between studies also due to different data collection tools across cohorts worldwide (e.g. FFQs, 24 h recall).

Due to this complexity, improving objective categorization of UPFs is an urgent need. Approaches such as stratifying UPFs by the number of processing steps, presence of additives or components of concern (e.g. added sugars, sodium), nutrient ratios, and total ingredient counts have been proposed to improve classification accuracy.^171^ Machine learning methods, like the recent algorithm predicting Nova classification with 73% accuracy,^172^ highlight the potential of predictive modelling, although current tools typically rely more on nutrient composition than ingredient or processing information, which are critical drivers of classification.^16,173^ Technological innovations such as sensor-based dietary monitoring and biomarker identification will possibly help overcome limitations of self-reported intake data.^174,175^

However, limitations remain due to incomplete data in food composition tables and dietary assessment tools, which frequently lack details on additives, brand names, and processing specifics.^170,176^

*Table 3* summarizes key actions that can help research on the impact of UPFs on CV health.

**Table 3:** Objectives and key actions for future research on the complex relationship between ultra-processed food (UPF) consumption and cardiovascular health

Objectives	Key actions
**To improve data quality as well as comparability across studies**	Development and validation of standardized, user-friendly dietary assessment methods focused on UPF consumption
**To boost the precision of UPF classification and measurement**	Use of machine learning, metabolomic profiling, and linkage of individual dietary data with commercial purchasing databases
**To overcome limitations of self-reported intake data, providing more reliable and detailed consumption patterns**	Use of technological innovations such as sensor-based dietary monitoring and biomarker
**To establish causal relationships between UPF intake and cardiovascular outcomes**	Prioritization of large-scale longitudinal cohort studies and randomized controlled trials
**To identify novel biological pathways and intervention targets**	Focus on mechanistic research on non-nutrient components of UPFs (e.g. cosmetic food additives, plasticizers, food matrix)
**To understand the social and environmental determinants of UPF consumption, especially in vulnerable populations**	Partnerships between researchers, clinicians, policymakers, and communities
**To ensure relevance and improve the adoption of dietary recommendations in future dietary guidelines**	Incorporation of patient and community perspectives into research design and educational initiatives
**To translate research findings into actionable public health interventions, including improved labelling, marketing restrictions, and economic incentives aimed at promoting healthier dietary choices**	Sustained stakeholder engagement and policy advocacy

## Conclusions

The continuing rise of UPF consumption is a leading global public health concern and poses significant challenges for the scientific community across multiple disciplines.

Although most of the current evidence linking UPFs to CV risk comes from epidemiological observational studies—with inherent risks of residual confounding, reverse causation, and bias—the growing body of data indicates that UPFs deserve serious attention as a modifiable factor in CV health prevention and clinical management.

General cardiologists should be aware of the potential CV risks associated with UPF intake and be prepared to offer the focused guidance provided in this document to help patients mitigate this emerging risk factor.

This clinical consensus statement that brought together European experts from diverse research fields is primarily intended to inform and support general cardiologists in their clinical practice.

This statement aims to strengthen cardiologists’ understanding of the link between UPF consumption and CV risk and to help the cardiologists to give advices regarding this emerging risk factor in clinical practice. In addition, this statement may guide researchers, public health professionals, and policymakers in developing unified strategies to improve CV outcomes and to encourage advocacy efforts aimed at reducing UPF consumption. Given the complexity of UPFs and their multifaceted and multisectoral impact, coordinated efforts across clinical practice, research, and public health are needed to ensure CV health remains a priority as dietary patterns continue to evolve.

## Supplementary Material

ehag226_Supplementary_Data

## Declarations

### Data Availability

No data were generated or analysed for or in support of this paper.
