# Supplementary material for: Ultra-processed foods, lifestyle management, and cardiovascular diseases: A clinical consensus statement of the European Society of Cardiology Council for Cardiology Practice and the European Association of Preventive Cardiology of the European Society of Cardiology
Source: Eur Heart J. 2026 May 6;47(27):3456–73. doi: 10.1093/eurheartj/ehag226 (PMC13364083; doi:10.1093/eurheartj/ehag226)
Supplement: ehag226_Supplementary_Data [file ehag226_supplementary_data.zip › Supplementary material 1 Supplementary Table 1.docx]

**Supplementary Material 1- table**

**Supplementary Table 1.** Definitions of Nova food groups according to extent and purpose of industrial food processing.

| **Nova Groups** | **Definition** | **Examples** |
| --- | --- | --- |
| **Group 1. Unprocessed**  **or minimally**  **processed foods** | Unprocessed foods: edible parts of plants (fruits, seeds, leaves, stems, roots, tubers) or of animals (muscle, offals, eggs, milk), and also fungi, algae and water, after separation from nature.  Minimally processed foods: unprocessed foods altered by industrial processes such as removal of inedible or unwanted parts, drying,  crushing, grinding, fractioning, roasting, toasting, boiling, pasteurization, refrigeration, freezing, placing in containers, vacuum packaging, non-alcoholic fermentation, and other methods that largely preserve the food matrix and do not add salt, sugar, oils or fats or other food substances to the original food. The main aim of these processes is to extend the life of unprocessed foods, enabling their storage for longer use, and, often, to make their preparation easier or more diverse. Additives are usually not necessary and only exceptionally found in minimally processed foods. | Fresh, squeezed, chilled, frozen or dried fruits and leafy and root vegetables; grains such as brown, parboiled or white rice, corn cob or kernel, wheat berry or grain; legumes such as beans, lentils and chickpeas; starchy roots and tubers such as potatoes, sweet potatoes and cassava; fungi such as fresh or dried mushrooms and yeast; meat, poultry, fish and seafood, whole or in the form of steak fillets and other cuts, fresh or chilled or frozen; eggs; fresh or pasteurized milk; fresh or pasteurized plain yogurt; fresh or pasteurized  fruit or vegetable juices (with no added sugar, sweeteners or flavours); grits, flakes or flour made from corn, wheat, oats or cassava; raw or toasted tree and ground nuts and other oily seeds (with no added salt or sugar); herbs and spices used in culinary preparations, such as thyme, oregano, mint, pepper, cloves and cinnamon, whole or powdered, fresh or dried; tea,  coffee and drinking water. Also, foods made up of two or more items in this group, such as dried mixed fruits, granola made from cereals, nuts and dried fruits with no added sugar, honey  or oil; pasta, couscous and polenta made with flours, flakes or grits and water; and foods with vitamins and minerals added generally to replace nutrients lost during processing, such as wheat or corn flour fortified with iron and folic acid. |
| **Group 2. Processed culinary**  **ingredients** | Substances obtained directly from group 1 foods or from nature by industrial processes such as pressing, centrifuging, extracting, refining,  dewatering and mining. Processes here aid in the creation of products used in the seasoning and cooking of group 1 foods and their use in dishes and meals prepared from scratch. Additives are usually not necessary and only exceptionally found in processed culinary ingredients. | Vegetable oils crushed from seeds, nuts or fruits (notably olives); butter and lard obtained from milk and pork; sugar and molasses obtained from cane or beet; honey extracted from combs and syrup from maple trees; vinegar; starches extracted from corn and other plants, and salt mined or from seawater. Also, products consisting of two group 2 items, such  as salted butter, and group 2 items with added vitamins or minerals, such as iodized salt. |
| **Group 3. Processed foods** | Relatively simple industrially manufactured food products made by adding at least one group 2 ingredient (such as salt, sugar, oil or fat) to group 1 foods, using preservation methods such as canning and bottling, and, in  the case of breads and cheeses, using non-alcoholic fermentation and boiling or baking. Processes and ingredients here aim to increase the durability of group 1 foods and make them more enjoyable by modifying or  enhancing their sensory qualities. Processed foods often contain additives that prolong product duration, protect original properties or prevent proliferation of microorganisms (such as preservatives and antioxidants),  but not additives with cosmetic functions (see next group). | All canned or bottled vegetables and legumes in brine; salted or sugared nuts and seeds; fruits in syrup; and dried or canned fish. Breads, cheese, pastries, cakes, cookies (biscuits); sweet or savoury snacks; cured meats; and ready-to-heat products such as burgers, and pre-prepared pies and pasta and pizza dishes when these products are made exclusively from group 1 foods and salt, oil, sugar or other Nova group 2 ingredients and do not contain classes of additives with cosmetic function. |
| **Group 4. Ultra-processed**  **foods** | Industrially manufactured food products made up of several ingredients (formulations) including sugar, oils, fats and salt (generally in combination and in higher amounts than in processed foods) and food substances of no or rare culinary use (such as high-fructose corn syrup, hydrogenated oils, modified starches and protein isolates). Group 1 foods are absent or represent a small proportion of the ingredients in the formulation.  Processes enabling the manufacture of ultra-processed foods include  industrial techniques such as extrusion, moulding and pre-frying;  application of additives including those whose function is to make the  final product palatable or hyper-palatable such as flavours, colourants,  non-sugar sweeteners and emulsifiers; and sophisticated packaging,  usually with synthetic materials. Processes and ingredients here are  designed to create highly profitable (low-cost ingredients, long shelf-life,  emphatic branding), convenient (ready-to-(h)eat or to drink), tasteful  alternatives to all other Nova food groups and to freshly prepared dishes  and meals. Ultra-processed foods are operationally distinguishable from  processed foods by the presence of food substances of no culinary use  (Varieties of sugars such as fructose, high-fructose corn syrup, ‘fruit juice  concentrates’, invert sugar, maltodextrin, dextrose and lactose; modified starches; modified oils such as hydrogenated or interesterified oils;  and protein sources such as hydrolysed proteins, soya protein isolate, gluten, casein, whey protein and ‘mechanically separated meat’) or of additives with cosmetic functions (flavours, flavour enhancers, colours,  emulsifiers, emulsifying salts, sweeteners, thickeners and anti-foaming, bulking, carbonating, foaming, gelling and glazing agents) in their list of ingredients. | All carbonated soft drinks; reconstituted fruit juices and ‘fruit’  drinks; ‘cocoa’ and other dairy drinks, and energy drinks; flavoured yogurt; candies (confectionery); margarines; poultry and fish ‘nuggets’ and ‘sticks’, sausages, hot dogs, luncheon  meats and other reconstituted meat products; plant-based meat substitutes; extruded breakfast ‘cereals’; powdered ‘instant’ soups, noodles and desserts; infant formulas and ‘follow-on’ milks; and ‘health’ and ‘slimming’ products such as  meal-replacement shakes and powders. Breads, pastries, cakes, cookies (biscuits); sweet or savoury  snacks; cured meats; and ready-to-heat products such as burgers, and pre-prepared pies and pasta and pizza dishes when these products are made up of food substances of no culinary use and or contain classes of additives with cosmetic function. |
