# Supplementary material for: Ultra-processed foods, lifestyle management, and cardiovascular diseases: A clinical consensus statement of the European Society of Cardiology Council for Cardiology Practice and the European Association of Preventive Cardiology of the European Society of Cardiology
Source: Eur Heart J. 2026 May 6;47(27):3456–73. doi: 10.1093/eurheartj/ehag226 (PMC13364083; doi:10.1093/eurheartj/ehag226)
Supplement: ehag226_Supplementary_Data [file ehag226_supplementary_data.zip › Supplementary material 2 Systematic Review.docx]

**Supplementary Material 2. Systematic review**

**Systematic review of prospective and intervention studies examining the association of ultra-processed food consumption with cardiovascular risk factors and outcomes – *including Supplementary Table 2, Table 3, Table 4, Table 5; Supplementary Figure 1 -***

**Methodology**

This systematic review was conducted according to the Preferred Reporting Items for Systematic Review and Meta-analyses (PRISMA) guidelines (*Page MJ et al. The PRISMA 2020 statement: an updated guideline for reporting systematic reviews. BMJ. 2021 Mar 29;372:n71. doi: 10.1136/bmj.n71*).

**Information sources and search strategy**

A search strategy was conducted to explore the evidence available on the influence of UPF consumption on lifestyle management and CV disease in four databases (PubMed, MEDLINE, Embase, and Scopus) from inception to 30^th^ July 2025 to identify relevant articles for inclusion in this review. No publication date restrictions were applied. Searches were conducted using pre-defined keywords relating to UPF consumption and CVD with MeSH terms utilized where appropriate (refer to the search strategy in **Supplementary Table 2**). Also, the reference lists of the included studies were referred to find potential studies for inclusion in the review. The studies and PDF files were managed and stored using Rayyan Software (an automation tool for systematic reviews) and Mendeley Reference Manager version 2.136.0.

**Inclusion/ Eligibility criteria**

- For this systematic review, longitudinal studies and randomized controlled trials (RCTs) conducted among a general adult population (>18 years) that explored the influence of UPF consumption on CV disease were included.
- The following CV related outcomes were: Atrial fibrillation, heart failure, arrythmias, CVD mortality, cardiovascular risk factors, primary incident CVD (fatal and non-fatal cases), recurrent CV disease, lipid profile, comorbidities, including obesity, diabetes, and hypertension, metabolic syndrome, non-alcoholic fatty liver, and chronic kidney disease.
- Finally, studies were only included if the exposure (UPF) was categorized according to the Nova classification.

**Exclusion criteria**

- Studies having case-control and cross-sectional study designs, abstracts, editorials, case reports, letters, systematic reviews, conference papers, book chapters, thesis publications etc., animal model studies, and studies involving pregnant women, children and teens (<18 years) were excluded.
- Studies in non-English languages were excluded.

**Selection process**

Rayyan software—an automated web tool (*Ouzzani M et al. Rayyan-a web and mobile app for systematic reviews. Syst Rev. 2016;5:210. doi: 10.1186/s13643-016-0384-4*) was used to identify duplicate articles from all the databases. The title and abstract of the research articles were screened for potential inclusion in the systematic review. Once considered, the full-text versions of the articles were retrieved and evaluated further for inclusion according to the inclusion criteria specified above. The selection process for CV disease and other health outcomes is given using a PRISMA flowchart, version 2020 (**Supplementary** **Figure 1**).

**Data extraction and data items**

The following data were extracted from the included studies: Surname of the first author, publication year, country, establishment year of the cohort, type of study design, sample size, measurement of UPF consumption, measurements used for CV outcome, dietary assessment methods used, guidelines used to ascertain CV related outcomes, statistical methods, covariates, and results (95% confidence intervals and p values). Two independent reviewers (SS and MB) assessed data upon extraction. The data on the sample, methods, and results from each included study were extracted by 1 author (SS) and cross-checked by another reviewer (MB). In the data extraction phase, studies were only included in the review upon mutual agreement of both reviewers (SS and MB).

**Quality assessment/ risk of bias assessment**

The National Heart, Lung and Blood Institute (NIH) Quality Assessment Tools (*Study Quality Assessment Tools | NHLBI, NIH [Internet]. [cited 2023 Apr 19]. Available from: https://www.nhlbi.nih.gov/health-topics/study-quality-assessment-tools*) for observational cohort studies, and controlled intervention studies separately, were used to assess the quality of the included studies in this systematic review.

The NIH tool had fourteen criteria and rated the risk of bias quality rating as ‘Good’, ‘Fair’, and ‘Poor.’ The quality assessments for individual studies for CVD health outcomes are summarized in **Supplementary Tables 3A-3C**.

**Results of the systematic review**

**Literature Selection**

**UPF consumption and CV disease and other health outcomes**

A total number of 11,297 records were identified for the relationship between UPF consumption and CVD outcomes from EMBASE (including MEDLINE and PubMed) and Scopus. After duplicate records were excluded using the automated tool-Rayyan software, 2943 were screened based on title and abstract. Of which, 2880 studies were excluded due to reasons deemed as irrelevant (refer PRISMA flow chart, **Supplementary** **Figure 1**). Following the screening and full-text retrieval process, 63 studies were finally included in the systematic review, of which 60 were prospective cohort studies and 3 were RCTs **(refer to Supplementary Tables 5 and 6).** Of the 63 studies included, most studies examined outcomes including, CV mortality (n=13) (108, 109, 115-125), T2D (n=10) (54-63), obesity (n=9) (44-52), incident CV disease (n=5) (108, 110-113), as compared to other outcomes such as, hypertension (n=5) (67-71), NAFLD (n=5) (89-93), CKD (n=5) (98-102), heart failure (n=3), (98, 108, 109), two each for metabolic syndrome (82, 83), dyslipidaemia (75, 76), and comorbidities (12, 14) and finally, one study each for CV risk factors (13), atrial fibrillation (107), and recurrent CV disease (115).

**Quality assessment/ risk of bias assessment findings**

The NIH tool was used to assess the risk of bias in the included studies (refer to **Supplementary Material Table 3A, 3B and 3C** for a summary of risk of bias assessment findings for prospective cohort and RCT studies). All studies that explored outcomes including CV mortality, CKD, comorbidities, CV risk factors, obesity, metabolic syndrome, dyslipidaemia, NAFLD, recurrent CV disease and diabetes mellitus scored as ‘good’ deeming them at low risk of bias. However, among prospective cohort studies, one (113) out of five studies exploring CVD health outcomes and two (67, 69) out of five studies exploring hypertension, scored ‘fair’ overall due to reasons including the selection of covariates and unclear definition of outcome in the study.

Among RCTs, one (51) out of three studies exploring obesity outcomes scored ‘fair’ overall due to reasons including inadequate randomization method, improper treatment concealment, and lack of treatment blinding to participants and providers.

**Supplementary Figure 1: The PRISMA 2020 flow chart shows the study selection process. Identification of studies via databases and registers for the association between ultra-processed foods in lifestyle management and cardiovascular diseases.**

**
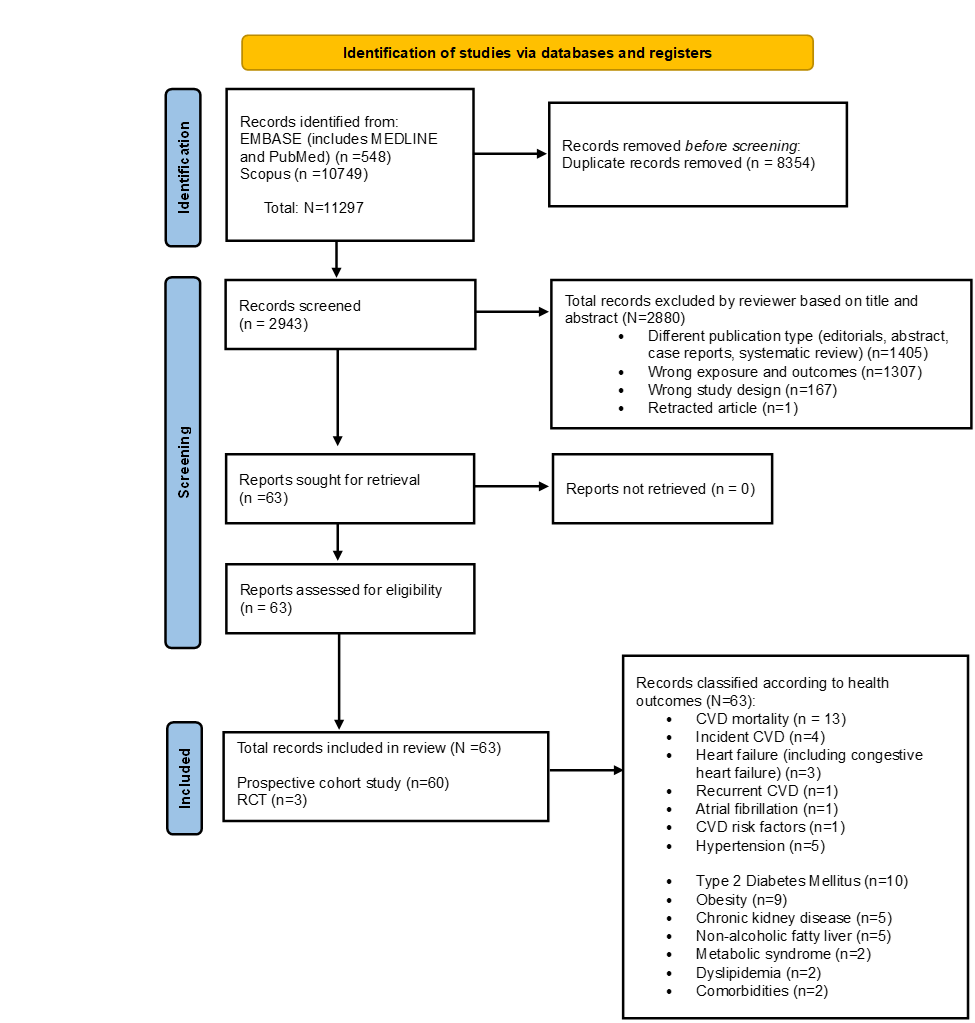
**

**Supplementary Table 2: Search strategy on EMBASE (includes PubMed and Medline) and Scopus to explore the influence of UPF consumption on CVD outcomes**

| **SEARCH STRINGS FOR EMBASE (includes PubMed and Medline within the search EMBASE filters) and Scopus: search conducted on 30th July 2025** |
| --- |
| **CARDIOVASCULAR HEALTH OUTCOMES** |
| 1. ('ultra'/exp OR ultra) AND processed AND ('food'/exp OR food) AND by AND ('nova'/exp OR nova) AND ('classification'/exp OR classification) AND ('cardiovascular'/exp OR cardiovascular) AND ('health'/exp OR health) AND outcomes; AND ([embase]/lim OR [medline]/lim OR [pubmed-not-medline]/lim) AND [<1966-2025]/py |
| 2. foods; AND ('ultra'/exp OR ultra) AND processed AND ('food'/exp OR food) AND by AND ('nova'/exp OR nova) AND classification; AND ('adult'/exp OR adult) AND upf AND consumption; AND ('cardiovascular'/exp OR cardiovascular) AND ('health'/exp OR health) AND outcomes; AND ([embase]/lim OR [medline]/lim OR [pubmed-not-medline]/lim) AND [<1966-2025]/py |
| 3. foods; AND ('ultra'/exp OR ultra) AND processed AND ('food'/exp OR food) AND by AND ('nova'/exp OR nova) AND classification; AND ('adult'/exp OR adult) AND upf AND consumption; AND ('cardiovascular'/exp OR cardiovascular) AND ('health'/exp OR health) AND outcomes; AND ([embase]/lim OR [medline]/lim OR [pubmed-not-medline]/lim) AND [<1966-2025]/py |
| **INCIDENT CARDIOVASCULAR EVENTS** |
| 4. ('ultra'/exp OR ultra) AND processed AND ('food'/exp OR food) AND by AND ('nova'/exp OR nova) AND classification; AND ('adult'/exp OR adult) AND upf AND consumption; AND atrial AND fibrillation; AND ('heart'/exp OR heart) AND failure; AND hf; AND arrythmias; AND ([embase]/lim OR [medline]/lim OR [pubmed-not-medline]/lim) AND [<1966-2025]/py |
| 5. ('ultra'/exp OR ultra) AND processed AND ('food'/exp OR food) AND by AND ('nova'/exp OR nova) AND classification; AND ('adult'/exp OR adult) AND upf AND consumption; AND ; AND incident AND cvd; AND cvd AND mortality; AND ([embase]/lim OR [medline]/lim OR [pubmed-not-medline]/lim) AND [<1966-2025]/py |
| 6. ('ultra'/exp OR ultra) AND processed AND ('food'/exp OR food) AND ('arrythmia'/exp OR arrythmia) AND ('atrial fibrillation'/exp OR 'atrial fibrillation') AND ('heart failure'/exp OR 'heart failure') AND ([embase]/lim OR [medline]/lim OR [pubmed-not-medline]/lim) AND [<1966-2025]/py |
| 7. ultra AND processed AND food AND by AND nova AND classification; AND adult AND upf AND consumption; AND cardiovascular AND risk AND factors; AND lipid AND profile; |
| **CARDIOVASCULAR MORTALITY** |
| 8. ('ultra'/exp OR ultra) AND processed AND ('food'/exp OR food) AND by AND ('nova'/exp OR nova) AND classification; AND cvd AND mortality; AND ([embase]/lim OR [medline]/lim OR [pubmed-not-medline]/lim) AND [<1966-2025]/py |
| **COMORBIDITIES** |
| 9. ('ultra'/exp OR ultra) AND processed AND ('food'/exp OR food) AND by AND ('nova'/exp OR nova) AND classification; AND ('lipid'/exp OR lipid) AND ('profile'/exp OR profile) AND ([embase]/lim OR [medline]/lim OR [pubmed-not-medline]/lim) AND [<1966-2025]/py |
| 10. ('ultra'/exp OR ultra) AND processed AND ('food'/exp OR food) AND by AND ('nova'/exp OR nova) AND ('classification'/exp OR classification) AND comorbidities; AND obesity; AND ('diabetes,'/exp OR diabetes,) AND hypertension; AND metabolic AND syndrome; AND 'non alcoholic' AND fatty AND liver; AND chronic AND ('kidney'/exp OR kidney) AND disease; AND ([embase]/lim OR [medline]/lim OR [pubmed-not-medline]/lim) AND [<1966-2025]/py |
| 11. ('ultra'/exp OR ultra) AND processed AND ('food'/exp OR food) AND by AND ('nova'/exp OR nova) AND ('classification'/exp OR classification) AND comorbidities; AND ([embase]/lim OR [medline]/lim OR [pubmed-not-medline]/lim) AND [<1966-2025]/py |
| 12. ('ultra' OR 'ultra'/exp OR ultra) AND processed AND ('food' OR 'food'/exp OR food) AND by AND ('nova' OR 'nova'/exp OR nova) AND ('classification' OR 'classification'/exp OR classification) AND ('obesity'/exp OR 'obesity') AND ([embase]/lim OR [medline]/lim OR [pubmed-not-medline]/lim) AND [<1966-2025]/py |
| 13. ('ultra' OR 'ultra'/exp OR ultra) AND processed AND ('food' OR 'food'/exp OR food) AND by AND ('nova' OR 'nova'/exp OR nova) AND ('classification' OR 'classification'/exp OR classification) AND ('diabetes mellitus'/exp OR 'diabetes mellitus') AND ([embase]/lim OR [medline]/lim OR [pubmed-not-medline]/lim) AND [<1966-2025]/py |
| 14. ('ultra'/exp OR ultra) AND processed AND ('food'/exp OR food) AND by AND ('nova'/exp OR nova) AND classification; AND hypertension AND ([embase]/lim OR [medline]/lim OR [pubmed-not-medline]/lim) AND [<1966-2025]/py |
| 15. ('ultra'/exp OR ultra) AND processed AND ('food'/exp OR food) AND by AND ('nova'/exp OR nova) AND classification; AND metabolic AND syndrome AND ([embase]/lim OR [medline]/lim OR [pubmed-not-medline]/lim) AND [<1966-2025]/py |
| 16. ('ultra'/exp OR ultra) AND processed AND ('food'/exp OR food) AND by AND ('nova'/exp OR nova) AND classification; AND 'non alcoholic' AND fatty AND liver AND ([embase]/lim OR [medline]/lim OR [pubmed-not-medline]/lim) AND [<1966-2025]/py |
| 17. ('ultra'/exp OR ultra) AND processed AND ('food'/exp OR food) AND by AND ('nova'/exp OR nova) AND classification; AND chronic AND kidney AND disease AND ([embase]/lim OR [medline]/lim OR [pubmed-not-medline]/lim) AND [<1966-2025]/py |
| **CARDIOVASCULAR PRIMARY PREVENTION** |
| 18. ('ultra'/exp OR ultra) AND processed AND ('food'/exp OR food) AND by AND ('nova'/exp OR nova) AND classification; AND primary AND prevention AND ([embase]/lim OR [medline]/lim OR [pubmed-not-medline]/lim) AND [<1966-2025]/py |
| **CARDIOVASCULAR RISK FACTORS** |
| 19. ('ultra'/exp OR ultra) AND processed AND ('food'/exp OR food) AND by AND ('nova'/exp OR nova) AND ('classification'/exp OR classification) AND ('cardiovascular'/exp OR cardiovascular) AND ('risk'/exp OR risk) AND factors AND ([embase]/lim OR [medline]/lim OR [pubmed-not-medline]/lim) AND [<1966-2025]/py |

**Supplementary Tables 3**

**Supplementary Table 3A: Analysis of the NIH quality criteria for prospective studies (14-criteria)**

| **Criteria^^[[1]](#footnote-1)^^** | Bonaccio et al., 2021 | Bonaccio et al., 2022 | Chen et al., 2022 | Torres-Collado et al., 2024 | Dehghan et al., 2023 | Fang et al., 2024 | Gauci et al., 2025 | Juul et al., 2021 | Kermani-Alghoraishi et al., 2024 |
| --- | --- | --- | --- | --- | --- | --- | --- | --- | --- |
| 1. Was the research question or objective in this paper clearly stated? | Y | Y | Y | Y | Y | Y | Y | Y | Y |
| 2. Was the study population clearly specified and defined? | Y | Y | Y | Y | Y | Y | Y | Y | Y |
| 3. Was the participation rate of eligible persons at least 50%? | NR | NR | NR | NR | NR | NR | NR | NR | NR |
| 4. Were all the subjects selected or recruited from the same or similar populations (including the same time period)? Were inclusion and exclusion criteria for being in the study prespecified and applied uniformly to all participants? | Y | Y | Y | Y | N | N | N | Y | Y |
| 5. Was a sample size justification. power description. or variance and effect estimates provided? | N | N | N | N | N | N | N | N | N |
| 6. For the analyses in this paper were the exposure(s) of interest measured prior to the outcome(s) being measured? | Y | Y | Y | Y | Y | Y | Y | Y | Y |
| 7. Was the timeframe sufficient so that one could reasonably expect to see an association between exposure and outcome if it existed? | Y | Y | Y | Y | Y | Y | Y | Y | Y |
| 8. For exposures that can vary in amount or level. did the study examine different levels of the exposure as related to the outcome (e.g., categories of exposure or exposure measured as continuous variable)? | Y | Y | Y | Y | Y | Y | Y | Y | Y |
| 9. Were the exposure measures (independent variables) clearly defined. valid. reliable. and implemented consistently across all study participants? | Y | Y | Y | Y | Y | Y | Y | Y | Y |
| 10. Was the exposure(s) assessed more than once over time? | Y | Y | Y | Y | Y | Y | Y | Y | Y |
| 11. Were the outcome measures (dependent variables) clearly defined. valid. reliable. and implemented consistently across all study participants? | Y | Y | Y | Y | Y | Y | Y | Y | Y |
| 12. Were the outcome assessors blinded to the exposure status of participants? | NR | NR | NR | NR | NR | NR | NR | NR | NR |
| 13. Was loss to follow-up after baseline 20% or less? | NR | NR | NR | NR | NR | NR | NR | NR | NR |
| 14. Were key potential confounding variables measured and adjusted statistically for their impact on the relationship between exposure(s) and outcome(s)? | Y | Y | Y | Y | Y | Y | Y | Y | Y |

| **Criteria^^[[2]](#footnote-2)^^** | Kityo et al., 2023 | Wang et al., 2025 | Zhao et al., 2024 | Zhong et al., 2024 | Du et al., 2021 | Jalali et al., 2024 | Srour et al., 2019 | Mendoza et al., 2024 | Sullivan et al., 2023 |
| --- | --- | --- | --- | --- | --- | --- | --- | --- | --- |
| 1. Was the research question or objective in this paper clearly stated? | Y | Y | Y | Y | Y | Y | Y | Y | Y |
| 2. Was the study population clearly specified and defined? | Y | Y | N | Y | Y | Y | Y | Y | Y |
| 3. Was the participation rate of eligible persons at least 50%? | NR | NR | NR | NR | NR | NR | NR | NR | NR |
| 4. Were all the subjects selected or recruited from the same or similar populations (including the same time period)? Were inclusion and exclusion criteria for being in the study prespecified and applied uniformly to all participants? | Y | Y | Y | Y | Y | Y | Y | Y | Y |
| 5. Was a sample size justification. power description. or variance and effect estimates provided? | N | N | N | N | N | N | N | N | N |
| 6. For the analyses in this paper were the exposure(s) of interest measured prior to the outcome(s) being measured? | Y | Y | Y | Y | Y | Y | Y | Y | Y |
| 7. Was the timeframe sufficient so that one could reasonably expect to see an association between exposure and outcome if it existed? | Y | Y | Y | Y | Y | Y | Y | Y | Y |
| 8. For exposures that can vary in amount or level. did the study examine different levels of the exposure as related to the outcome (e.g., categories of exposure or exposure measured as continuous variable)? | Y | Y | Y | Y | Y | Y | Y | Y | Y |
| 9. Were the exposure measures (independent variables) clearly defined. valid. reliable. and implemented consistently across all study participants? | Y | Y | Y | Y | Y | Y | Y | Y | Y |
| 10. Was the exposure(s) assessed more than once over time? | Y | Y | Y | Y | Y | N | Y | Y | Y |
| 11. Were the outcome measures (dependent variables) clearly defined. valid. reliable. and implemented consistently across all study participants? | Y | Y | N | Y | Y | Y | Y | Y | Y |
| 12. Were the outcome assessors blinded to the exposure status of participants? | NR | NR | NR | NR | NR | NR | NR | NR | NR |
| 13. Was loss to follow-up after baseline 20% or less? | NR | NR | NR | NR | NR | NR | NR | NR | NR |
| 14. Were key potential confounding variables measured and adjusted statistically for their impact on the relationship between exposure(s) and outcome(s)? | Y | Y | Y | Y | Y | N | Y | Y | Y |

| **Criteria^^[[3]](#footnote-3)^^** | Tu et al., 2023 | Palacios et al., 2023 | Rezende-Alves et al., 2021 | Li et al., 2022 | Monge et al., 2021 | Oladele et al., 2024 | Shim et al., 2022 | Canhada et al., 2023 |
| --- | --- | --- | --- | --- | --- | --- | --- | --- |
| 1. Was the research question or objective in this paper clearly stated? | Y | Y | Y | Y | Y | Y | Y | Y |
| 2. Was the study population clearly specified and defined? | Y | Y | N | Y | Y | Y | Y | Y |
| 3. Was the participation rate of eligible persons at least 50%? | NR | NR | NR | NR | NR | NR | NR | NR |
| 4. Were all the subjects selected or recruited from the same or similar populations (including the same time period)? Were inclusion and exclusion criteria for being in the study prespecified and applied uniformly to all participants? | Y | Y | Y | Y | N | Y | N | N |
| 5. Was a sample size justification. power description. or variance and effect estimates provided? | N | N | N | N | N | N | N | N |
| 6. For the analyses in this paper were the exposure(s) of interest measured prior to the outcome(s) being measured? | Y | Y | Y | Y | Y | Y | Y | Y |
| 7. Was the timeframe sufficient so that one could reasonably expect to see an association between exposure and outcome if it existed? | Y | Y | Y | Y | Y | Y | Y | Y |
| 8. For exposures that can vary in amount or level. did the study examine different levels of the exposure as related to the outcome (e.g., categories of exposure or exposure measured as continuous variable)? | Y | Y | Y | Y | Y | Y | Y | Y |
| 9. Were the exposure measures (independent variables) clearly defined. valid. reliable. and implemented consistently across all study participants? | Y | Y | Y | Y | N | Y | Y | Y |
| 10. Was the exposure(s) assessed more than once over time? | Y | Y | N | Y | Y | Y | Y | Y |
| 11. Were the outcome measures (dependent variables) clearly defined. valid. reliable. and implemented consistently across all study participants? | Y | Y | N | N | Y | Y | Y | Y |
| 12. Were the outcome assessors blinded to the exposure status of participants? | NR | NR | NR | NR | NR | NR | NR | NR |
| 13. Was loss to follow-up after baseline 20% or less? | NR | NR | NR | NR | NR | NR | NR | NR |
| 14. Were key potential confounding variables measured and adjusted statistically for their impact on the relationship between exposure(s) and outcome(s)? | Y | Y | N | Y | N | Y | Y | N |

| **Criteria^^[[4]](#footnote-4)^^** | Chen et al., 2023 | Cho et al., 2024 | Du et al., 2024 | Duan et al., 2022 | Levy et al., 2021 | Li et al., 2022 | Moslehi et al., 2024 | Srour et al., 2020 | Llavero-Valero et al., 2021 | Cordova et al., 2021 |
| --- | --- | --- | --- | --- | --- | --- | --- | --- | --- | --- |
| 1. Was the research question or objective in this paper clearly stated? | Y | Y | Y | Y | Y | Y | Y | Y | Y | Y |
| 2. Was the study population clearly specified and defined? | Y | Y | Y | Y | N | Y | Y | Y | Y | Y |
| 3. Was the participation rate of eligible persons at least 50%? | NR | NR | NR | NR | NR | NR | NR | NR | NR | NR |
| 4. Were all the subjects selected or recruited from the same or similar populations (including the same time period)? Were inclusion and exclusion criteria for being in the study prespecified and applied uniformly to all participants? | Y | Y | Y | Y | Y | Y | Y | Y | Y | Y |
| 5. Was a sample size justification. power description. or variance and effect estimates provided? | N | N | N | N | N | N | N | N | N | N |
| 6. For the analyses in this paper were the exposure(s) of interest measured prior to the outcome(s) being measured? | Y | Y | Y | Y | Y | Y | Y | Y | Y | Y |
| 7. Was the timeframe sufficient so that one could reasonably expect to see an association between exposure and outcome if it existed? | Y | Y | Y | Y | Y | Y | Y | Y | Y | Y |
| 8. For exposures that can vary in amount or level. did the study examine different levels of the exposure as related to the outcome (e.g., categories of exposure or exposure measured as continuous variable)? | Y | Y | Y | Y | Y | Y | Y | Y | Y | Y |
| 9. Were the exposure measures (independent variables) clearly defined. valid. reliable. and implemented consistently across all study participants? | Y | Y | Y | Y | Y | Y | Y | Y | Y | Y |
| 10. Was the exposure(s) assessed more than once over time? | Y | N | Y | Y | Y | Y | Y | Y | Y | Y |
| 11. Were the outcome measures (dependent variables) clearly defined. valid. reliable. and implemented consistently across all study participants? | Y | Y | Y | Y | Y | Y | Y | Y | Y | N |
| 12. Were the outcome assessors blinded to the exposure status of participants? | NR | NR | NR | NR | NR | NR | NR | NR | NR | NR |
| 13. Was loss to follow-up after baseline 20% or less? | NR | NR | NR | NR | NR | NR | NR | NR | NR | NR |
| 14. Were key potential confounding variables measured and adjusted statistically for their impact on the relationship between exposure(s) and outcome(s)? | Y | Y | Y | Y | Y | Y | Y | Y | Y | N |

| **Criteria^^[[5]](#footnote-5)^^** | Beslay et al., 2020 | Mendonça et al., 2016 | Sandoval-Insausti et al., 2020 | Li et al., 2021 | Rauber et al., 2020 | Cai et al., 2022 | Du et al., 2022 | Garcia et al., 2021 | Gu et al., 2023 | Fu et al., 2025 | Konieczna et al., 2022 | Zhang et al., 2024 | Zhao et al., 2024 | Garcia et al., 2025 |
| --- | --- | --- | --- | --- | --- | --- | --- | --- | --- | --- | --- | --- | --- | --- |
| 1. Was the research question or objective in this paper clearly stated? | Y | Y | Y | Y | Y | Y | Y | Y | Y | Y | Y | Y | Y | Y |
| 2. Was the study population clearly specified and defined? | Y | Y | Y | Y | Y | Y | Y | Y | Y | N | Y | Y | Y | Y |
| 3. Was the participation rate of eligible persons at least 50%? | NR | NR | NR | NR | NR | NR | NR | NR | NR | NR | NR | NR | NR | NR |
| 4. Were all the subjects selected or recruited from the same or similar populations (including the same time period)? Were inclusion and exclusion criteria for being in the study prespecified and applied uniformly to all participants? | Y | Y | Y | Y | Y | N | Y | Y | Y | Y | Y | Y | Y | Y |
| 5. Was a sample size justification. power description. or variance and effect estimates provided? | N | N | N | N | N | N | N | N | N | N | N | N | N | N |
| 6. For the analyses in this paper were the exposure(s) of interest measured prior to the outcome(s) being measured? | Y | Y | Y | Y | Y | Y | Y | Y | Y | Y | Y | Y | Y | Y |
| 7. Was the timeframe sufficient so that one could reasonably expect to see an association between exposure and outcome if it existed? | Y | Y | Y | Y | Y | Y | Y | Y | Y | Y | Y | Y | Y | Y |
| 8. For exposures that can vary in amount or level. did the study examine different levels of the exposure as related to the outcome (e.g., categories of exposure or exposure measured as continuous variable)? | Y | Y | Y | Y | Y | Y | Y | Y | Y | Y | Y | Y | Y | Y |
| 9. Were the exposure measures (independent variables) clearly defined. valid. reliable. and implemented consistently across all study participants? | Y | Y | Y | Y | Y | Y | Y | Y | Y | Y | Y | Y | Y | Y |
| 10. Was the exposure(s) assessed more than once over time? | Y | Y | Y | Y | Y | Y | Y | Y | Y | Y | Y | Y | Y | Y |
| 11. Were the outcome measures (dependent variables) clearly defined. valid. reliable. and implemented consistently across all study participants? | Y | Y | Y | Y | Y | Y | Y | Y | Y | N | Y | Y | Y | Y |
| 12. Were the outcome assessors blinded to the exposure status of participants? | NR | NR | NR | NR | NR | NR | NR | NR | NR | NR | NR | NR | NR | NR |
| 13. Was loss to follow-up after baseline 20% or less? | NR | NR | NR | NR | NR | NR | NR | NR | NR | NR | NR | NR | NR | NR |
| 14. Were key potential confounding variables measured and adjusted statistically for their impact on the relationship between exposure(s) and outcome(s)? | Y | Y | Y | Y | Y | Y | Y | Y | Y | Y | Y | Y | Y | Y |

| **Criteria^^[[6]](#footnote-6)^^** | Pan et al., 2023 | DaSilva Magalhães et al., 2022 | Scaranni et al., 2022 | Vargas et al., 2021 | Bonaccio et al., 2023 | Liu et al., 2023 |
| --- | --- | --- | --- | --- | --- | --- |
| 1. Was the research question or objective in this paper clearly stated? | Y | Y | Y | Y | Y | Y |
| 2. Was the study population clearly specified and defined? | Y | Y | Y | Y | Y | Y |
| 3. Was the participation rate of eligible persons at least 50%? | NR | NR | NR | NR | NR | NR |
| 4. Were all the subjects selected or recruited from the same or similar populations (including the same time period)? Were inclusion and exclusion criteria for being in the study prespecified and applied uniformly to all participants? | Y | Y | Y | Y | Y | N |
| 5. Was a sample size justification. power description. or variance and effect estimates provided? | N | N | N | N | N | N |
| 6. For the analyses in this paper were the exposure(s) of interest measured prior to the outcome(s) being measured? | Y | Y | Y | Y | Y | Y |
| 7. Was the timeframe sufficient so that one could reasonably expect to see an association between exposure and outcome if it existed? | Y | Y | Y | Y | Y | Y |
| 8. For exposures that can vary in amount or level. did the study examine different levels of the exposure as related to the outcome (e.g., categories of exposure or exposure measured as continuous variable)? | Y | Y | Y | Y | Y | Y |
| 9. Were the exposure measures (independent variables) clearly defined. valid. reliable. and implemented consistently across all study participants? | Y | Y | Y | N | Y | Y |
| 10. Was the exposure(s) assessed more than once over time? | N | Y | Y | Y | Y | Y |
| 11. Were the outcome measures (dependent variables) clearly defined. valid. reliable. and implemented consistently across all study participants? | Y | Y | Y | Y | Y | Y |
| 12. Were the outcome assessors blinded to the exposure status of participants? | NR | NR | NR | NR | NR | NR |
| 13. Was loss to follow-up after baseline 20% or less? | NR | NR | NR | NR | NR | NR |
| 14. Were key potential confounding variables measured and adjusted statistically for their impact on the relationship between exposure(s) and outcome(s)? | N | N | N | Y | Y | Y |

**Supplementary Table 3B: Analysis of the NIH quality criteria for controlled intervention studies (14-criteria)**

| **Criteria^^[[7]](#footnote-7)^^** | Dicken et al., 2025 | Hamano et al., 2024 | Hall et al., 2019 |
| --- | --- | --- | --- |
| 1. Was the study described as randomized, a randomized trial, a randomized clinical trial, or an RCT? | Y | Y | Y |
| 2. Was the method of randomization adequate (i.e., use of randomly generated assignment)? | Y | N | Y |
| 3. Was the treatment allocation concealed (so that assignments could not be predicted)? | Y | N | N |
| 4. Were study participants and providers blinded to treatment group assignment? | Y | N | Y |
| 5. Were the people assessing the outcomes blinded to the participants' group assignments? | Y | N | N |
| 6. Were the groups similar at baseline on important characteristics that could affect outcomes (e.g., demographics, risk factors, co-morbid conditions)? | Y | Y | Y |
| 7. Was the overall drop-out rate from the study at endpoint 20% or lower of the number allocated to treatment? | N | N | N |
| 8. Was the differential drop-out rate (between treatment groups) at endpoint 15 percentage points or lower? | N | N | N |
| 9. Was there high adherence to the intervention protocols for each treatment group? | Y | Y | Y |
| 10. Were other interventions avoided or similar in the groups (e.g., similar background treatments)? | Y | Y | Y |
| 11. Were outcomes assessed using valid and reliable measures, implemented consistently across all study participants? | Y | Y | Y |
| 12. Did the authors report that the sample size was sufficiently large to be able to detect a difference in the main outcome between groups with at least 80% power? | N | N | N |
| 13. Were outcomes reported or subgroups analyzed prespecified (i.e., identified before analyses were conducted)? | N | N | N |
| 14. Were all randomized participants analyzed in the group to which they were originally assigned, i.e., did they use an intention-to-treat analysis? | Y | Y | Y |

**Supplementary Table 3C: NIH Quality Assessment of the included observational studies and controlled intervention studies for the association between ultra-processed foods in lifestyle management and cardiovascular diseases.**

| **Prospective studies** | Evaluator 1 | Evaluator 2 |  | Final Evaluation |  |
| --- | --- | --- | --- | --- | --- |
| **CVD mortality** |  |  |  |  |  |
| 1. Bonaccio et al., 2021 | Good | Good |  | Good |  |
| 1. Bonaccio et al., 2022 | Good | Good |  | Good |  |
| 1. Chen et al., 2022 | Good | Good |  | Good |  |
| 1. Torres-Collado et al., 2024 | Good | Good |  | Good |  |
| 1. Dehghan et al., 2023 | Good | Good |  | Good |  |
| 1. Fang et al., 2024 | Good | Fair |  | Good |  |
| 1. Gauci et al., 2025 | Good | Good |  | Good |  |
| 1. Juul et al., 2021 | Good | Good |  | Good |  |
| 1. Kermani-Alghoraishi et al., 2024 | Good | Fair |  | Good |  |
| 1. Kityo et al., 2023 | Good | Good |  | Good |  |
| 1. Wang et al., 2025 | Good | Good |  | Good |  |
| 1. Zhao et al., 2024 | Good | Fair |  | Good |  |
| 1. Zhong et al., 2024 | Good | Good |  | Good |  |
| **Incident CVD** |  |  |  |  |  |
| 1. Du et al., 2021 | Good | Good |  | Good |  |
| 1. Jalali et al., 2024 | Fair | Fair |  | Fair |  |
| 1. Srour et al., 2019 | Good | Good |  | Good |  |
| 1. Mendoza et al., 2024 | Good | Good |  | Good |  |
| 1. Juul et al., 2021 | Good | Good |  | Good |  |
| **Heart failure** |  |  |  |  |  |
| 1. Sullivan et al., 2023 | Good | Good |  | Good |  |
| 1. Dehghan et al., 2023 | Good | Good |  | Good |  |
| 1. Juul et al., 2021 | Good | Good |  | Good |  |
| **Recurrent CVD** |  |  |  |  |  |
| 1. Bonaccio et al., 2022 | Good | Good |  | Good |  |
| **Arrythmias** |  |  |  |  |  |
| 1. Tu et al., 2023 | Good | Good |  | Good |  |
| **CVD risk factors** | |  |  |  |  |
| 1. Palacios et al., 2023 | | Good | Good |  | Good |
| **Hypertension** |  |  |  |  |  |
| 1. Rezende-Alves et al., 2021 | Fair | Fair |  | Fair |  |
| 1. Li et al., 2022 | Good | Fair |  | Good |  |
| 1. Monge et al., 2021 | Fair | Fair |  | Fair |  |
| 1. Oladele et al., 2024 | Good | Good |  | Good |  |
| 1. Shim et al., 2022 | Good | Good |  | Good |  |
| **Diabetes Mellitus** |  |  |  |  |  |
| 1. Canhada et al., 2023 | Good | Fair |  | Good |  |
| 1. Chen et al., 2023 | Good | Good |  | Good |  |
| 1. Cho et al., 2024 | Good | Good |  | Good |  |
| 1. Du et al., 2024 | Good | Good |  | Good |  |
| 1. Duan et al., 2022 | Good | Good |  | Good |  |
| 1. Levy et al., 2021 | Good | Good |  | Good |  |
| 1. Li et al., 2022 | Good | Good |  | Good |  |
| 1. Moslehi et al., 2024 | Good | Good |  | Good |  |
| 1. Srour et al., 2020 | Good | Good |  | Good |  |
| 1. Llavero-Valero et al., 2021 | Good | Good |  | Good |  |
| **Obesity** |  |  |  |  |  |
| 1. Cordova 2021 et al., 2021 | Good | Fair |  | Good |  |
| 1. Beslay et al., 2020 | Good | Good |  | Good |  |
| 1. Mendonça et al., 2016 | Good | Good |  | Good |  |
| 1. Sandoval-Insausti et al., 2020 | Good | Good |  | Good |  |
| 1. Li et al., 2021 | Good | Good |  | Good |  |
| 1. Rauber et al., 2020 | Good | Good |  | Good |  |
| **Chronic Kidney Disease** |  |  |  |  |  |
| 1. Cai et al., 2022 | Good | Good |  | Good |  |
| 1. Du et al., 2022 | Good | Fair |  | Good |  |
| 1. Rey-Garcia et al., 2021 | Good | Good |  | Good |  |
| 1. Gu et al., 2023 | Good | Good |  | Good |  |
| 1. Sullivan et al., 2023 | Good | Good |  | Good |  |
| **NAFLD** |  |  |  |  |  |
| 1. Fu et al., 2025 | Good | Good |  | Good |  |
| 1. Konieczna et al., 2022 | Good | Good |  | Good |  |
| 1. Zhang et al., 2024 | Good | Good |  | Good |  |
| 1. Zhao et al., 2024 | Good | Good |  | Good |  |
| 1. Garcia et al., 2025 | Good | Good |  | Good |  |
| **Metabolic syndrome** |  |  |  |  |  |
| 1. Pan et al., 2023 | Good | Good |  | Good |  |
| 1. DaSilva Magalhães et al., 2022 | Good | Fair |  | Good |  |
| **Dyslipidaemia** |  |  |  |  |  |
| 1. Scaranni et al., 2022 | Good | Good |  | Good |  |
| 1. Donat-Vargas et al., 2021 | Good | Fair |  | Good |  |
| **Comorbidities** |  |  |  |  |  |
| 1. Bonaccio et al., 2023 | Good | Good |  | Good |  |
| 1. Liu et al., 2023 | Good | Good |  | Good |  |
| **Controlled intervention studies** | Evaluator 1 | Evaluator 2 |  | Final Evaluation |  |
| **Obesity** |  |  |  |  |  |
| 1. Dicken et al., 2025 | Good | Good |  | Good |  |
| 1. Hamano et al., 2024 | Fair | Fair |  | Fair |  |
| 1. Hall et al., 2019 | Good | Good |  | Good |  |

**Supplementary Table 4. Summary of evidence on the association between ultra-processed food consumption and cardiovascular (CV) risk factors and outcomes.**

|  | **Sample size** | **N of events** | **Association found** | **NIH quality**  **rating** |
| --- | --- | --- | --- | --- |
| ***CV risk factors*** |  |  |  |  |
| **Obesity** |  |  |  |  |
| Cordova 2021 et al., 2021^44^ | 348,748 | 191,255 (overweight); 103,259 (obese) | ✔️ Direct association | Good |
| Beslay et al., 2020^46^ | 110,390 | 3,066 (obese);  and 7,603 (overweight) | ✔️ Direct association | Good |
| Mendonça et al., 2016^45^ | 8,451 | 1,939 | ✔️ Direct association | Good |
| Sandoval-Insausti et al., 2020^47^ | 652 | 177 | ✔️ Direct association | Good |
| Li et al., 2021^47^ | 12,451 | 5,027 (overweight);  and 2,816 (obese) | ✔️ Direct association | Good |
| Rauber et al., 2020^48^ | 22,659 | 6,143 | ✔️ Direct association | Good |
| **Type 2 Diabetes** |  |  |  |  |
| Canhada et al., 2023^54^ | 10,202 | 1,799 | ✔️ Direct association | Good |
| Chen et al., 2023^55^ | 71,871 (NHS);  87,918 (NHS II);  and 38,847 (HPFS) | 8,591 (NHS);  7,177 (NHSII); ad 3735 (HPFS) | ✔️ Direct association | Good |
| Cho et al., 2024^56^ | 7,438 | 1,187 | ✔️ Direct association | Good |
| Du et al., 2024^57^ | 13,172 | 4,539 | ✔️ Direct association | Good |
| Duan et al., 2022^58^ | 70,421 | 1,128 | ✔️ Direct association | Good |
| Levy et al., 2021^59^ | 21,475 | 305 | ✔️ Direct association | Good |
| Li et al., 2022^60^ | 8,382 | 940 | ✔️ Direct association | Good |
| Moslehi et al., 2024^63^ | 2,457 | 766 | ✔️ Direct association | Good |
| Srour et al., 2020^61^ | 10,4707 | 821 | ✔️ Direct association | Good |
| Llavero-Valero et al., 2021^62^ | 20,060 | 175 | ✔️ Direct association | Good |
| **Hypertension** |  |  |  |  |
| Rezende-Alves et al., 2021^67^ | 1,221 | 370 | ✔️ Direct association | Fair |
| Li et al., 2022^68^ | 15,054 | 4,329 | ✔️ Direct association | Good |
| Monge et al., 2021^69^ | 64,934 | 3,752 | ❌ No association | Fair |
| Oladele et al., 2024^70^ | 5,957 | 2,134 | ✔️ Direct association | Good |
| Shim et al., 2022^71^ | 9,188 | 4,199 | ✔️ Direct association | Good |
| **Dyslipidaemia** |  |  |  |  |
| Scaranni et al., 2022^75^ | 5,275 | 629 (isolated hypercholesterolaemia); 857 (isolated hypertriacylglycerolaemia); 458 (mixed hyperlipidaemia);  and 842 low-HDL | ✔️ Direct association | Good |
| Donat-Vargas et al., 2021^76^ | 1,082 | 60 (hypertriglyceridemia); 112 (low HDL cholesterol); and 54 (high LDL cholesterol) | ✔️ Direct association | Good |
| **Metabolic syndrome** |  |  |  |  |
| Pan et al., 2023^82^ | 5,147 | 1,172 | ✔️ Direct association | Good |
| DaSilva Magalhães et al., 2022^83^ | 896 | 317 | ❌ No association | Good |
| **Non-alcoholic fatty liver disease** |  |  |  |  |
| Fu et al., 2025^90^ | 44,642 | 1,562 | ✔️ Direct association | Good |
| Konieczna et al., 2022^91^ | 5,867 | 5,585 (HIS >36) | ✔️ Direct association | Good |
| Zhang et al., 2024^92^ | 143,073 | 1,445 | ✔️ Direct association | Good |
| Zhao et al., 2024^89^ | 137,173 | 1,108 | ✔️ Direct association | Good |
| Garcia et al., 2025^93^ | 70 | ‒ | ✔️ Direct association | Good |
| **Chronic kidney disease** |  |  |  |  |
| Cai et al., 2022^99^ | 78,346 | 2,470 | ✔️ Direct association | Good |
| Du et al., 2022^100^ | 14,679 | 4,859 | ✔️ Direct association | Good |
| Rey-Garcia et al., 2021^101^ | 1,312 | 183 | ✔️ Direct association | Good |
| Gu et al., 2023^102^ | 102,332 (UK Biobank);  and 23,775 (TCLSIH) | 1,740 (UK Biobank); 261 (TCLSIH) | ✔️ Direct association | Good |
| Sullivan et al., 2023^98^ | 3,939 | 1,047 | ✔️ Direct association | Good |
|  |  |  |  |  |
| ***CV outcomes*** |  |  |  |  |
| **Arrythmias** |  |  |  |  |
| Tu et al., 2023^107^ | 121,300 | 4,579 | ✔️ Direct association | Good |
| **Heart failure** |  |  |  |  |
| Sullivan et al., 2023^98^ | 3,939 | 406 | ❌ No association | Good |
| Dehghan et al., 2023^109^ | 138,076 | 1,000 | ❌ No association | Good |
| Juul et al., 2021^108^ | 3,003 | 648 | ✔️ Direct association | Good |
| **Incident CVD** |  |  |  |  |
| Du et al., 2021^110^ | 13,548 | 2,006 | ✔️ Direct association | Good |
| Jalali et al., 2024^113^ | 2,050 | 208 | ✔️ Direct association | Fair |
| Srour et al., 2019^111^ | 105,159 | 1,409 | ✔️ Direct association | Good |
| Mendoza et al., 2024^112^ | 121,701 (NHS);  116, 340 (NHSII);  and 51,529 (HPFS) | 8446 (NHS); 1,668 (NHSII); and 6,686 (HPFS) | ✔️ Direct association | Good |
| Juul et al., 2021^108^ | 3,003 | 648 | ✔️ Direct association | Good |
| **CVD mortality** |  |  |  |  |
| Bonaccio et al., 2021 ^116^ | 22,475 | 439 | ✔️ Direct association | Good |
| Bonaccio et al., 2022^115^ | 1,171 | 178 | ✔️ Direct association | Good |
| Chen et al., 2022^117^ | 60,298 | 384 | ✔️ Direct association | Good |
| Torres-Collado et al., 2024^118^ | 1,538 | 114 | ✔️ Direct association | Good |
| Dehghan et al., 2023^109^ | 138,076 | 3073 | ✔️ Direct association | Good |
| Fang et al., 2024^119^ | 74,563 (NHS);  and 39,501 (HPFS) | 11,416 | ❌ No association | Good |
| Gauci et al., 2025^120^ | 39,544 | 4,229 | ✔️ Direct association | Good |
| Juul et al., 2021^108^ | 3,003 | 108 | ✔️ Direct association | Good |
| Kermani-Alghoraishi et al., 2024^121^ | 5,432 | 181 | ❌ No association | Good |
| Kityo et al., 2023^122^ | 113,576 | 539 | ❌ No association | Good |
| Wang et al., 2025^123^ | 7,138 | 859 | ❌ No association | Good |
| Zhao et al., 2024^125^ | 41, 070 (NHANES);  208, 051 (UK Biobank);  and 108, 714 (PLCO) | 1749 (NHANES); 1979 (UK Biobank); and 8,009 (PLCO) | ✔️ Direct association | Good |
| Zhong et al., 2024^124^ | 91,891 | 5490 | ✔️ Direct association | Good |
|  |  |  |  |  |
| **Randomized clinical trials** |  |  |  |  |
| Dicken et al., 2025 | 55 | ‒ | ✔️ Direct association | Good |
| Hamano et al., 2024 | 9 | ‒ | ✔️ Direct association | Fair |
| Hall et al., 2019 | 20 | ‒ | ✔️ Direct association | Good |

NHI = The National Heart, Lung and Blood Institute (NIH) Quality Assessment Tools; NHS = Nurses’ Health Study; NHSII = Nurses’ Health Study II; HPFS = Health Professionals Follow-up Study; TCLSIH = Tianjin Chronic Low-Grade Systemic Inflammation and Health.; HIS = hepatic steatosis index.

**Supplementary Table 5: Characteristics of included studies exploring the association between ultra-processed foods (UPFs) in lifestyle management and cardiovascular diseases.**

| **CARDIOVASCULAR MORTALITY** | | | | | | | | |
| --- | --- | --- | --- | --- | --- | --- | --- | --- |
| **PROSPECTIVE COHORT STUDIES** | | | | | | | | |
| **Serial number** | **Author, Year; Country** | **Period when study was conducted, follow-up period** | **Sample size (N)/ cases (%)** | **Study design, study population** | **Dietary assessment** | **Exposure:**  **UPF consumption** | **Outcome of interest** | **Outcome ascertainment** |
| 1. | Bonaccio et al., 2021^116^; Italy | 2005-2010, mortality follow-up till 2015 | N=22,475/CVD mortality cases, N= 439 (1.9%) | Prospective cohort study, general adult population | 188-item FFQ  NOVA classification was used to classify UPF.  To estimate UPF, the amount consumed (g/d) of each food group included in the fourth category of the NOVA classification (a total of 15 food groups and 3 beverages) were summed, and then calculated the proportion (%) of UPF in the total weight of food and beverages consumed (g/d) by creating a weight ratio. | UPF consumption | Risk of CVD mortality | CVD mortality included deaths from diseases of the circulatory system, when the underlying cause of death included ICD-9 codes 390–459, and were assessed by the Italian mortality registry (ReNCaMregistry),and validated by Italian death certificates (ISTAT form). |
| 2. | Bonaccio et al., 2022^115^; Italy | 2005-2010, mortality follow-up till 2018 | n=1,171 individuals with history of CVD at baseline; / CVD mortality cases, n=178 (15.2%) | Prospective cohort study, general adult population with CVD at baseline | 188-item FFQ  NOVA classification was used to classify UPF.  To estimate UPF, the amount consumed (g/d) of each food group included in the fourth category of the NOVA classification (a total of 15 food groups and 3 beverages) were summed, and then calculated the proportion (%) of UPF in the total weight of food and beverages consumed (g/d) by creating a weight ratio. | UPF consumption | Risk of CVD mortality | CVD mortality included deaths from diseases of the circulatory system, when the underlying cause of death included ICD-9 codes 390–459. |
| 3. | Chen et al., 2022^117^; UK | 2008-2020; followed up until 2020 | N=60298/ CVD mortality cases, n=384 (0.6%) | Prospective cohort study, general adult population | The 24-h  dietary recall questionnaire  (included 204 common food and drink items).  UPF consumption was estimated based on the  24-h dietary recall with typical serving sizes and appropriate total energy supply per 100 g or 100 ml to each food and beverage item to derive  an estimated energy intake values based on published UK data | UPF consumption | Risk of CVD mortality | Death, cause of death and incident CVD cases were obtained through linkage to the national health registries |
| 4. | Torres-Collado et al., 2024^118^; Spain | 1994-1995 | N=1538/CVD mortality cases, n=114 (7.4%) | Population-based study | 93-item semi-quantitative  FFQ | UPF consumption | Risk of CVD mortality | CVD mortality was ascertained using the code: CVD (ICD-10: I00-I99), and CVD-related deaths were verified via the national health registries. |
| 5. | Dehghan et al., 2023 ^109^; India and other countries | 2005-2016 | N=138,076/CVD mortality cases=3073 (2.2%) | Multinational prospective cohort study | 24 hour recalls and country-specific FFQ  UPFs intake was considered as a serving of intake per day rather than a proportion of energy. To examine the association between the consumption  of UPFs and outcomes, participants were grouped based on their intake  into 0 serving/d, <1 serving/d, 1 to <2 servings/d, and >2 servings/d of intake. | UPF consumption | Risk of CVD mortality | CVD death events were collected using standardized case-report forms and reported based on common definitions by medical physicians |
| 6. | Fang et al., 2024^119^; USA | Nurses Health study (NHS):1984-2018  Health professional’s follow-up study (HPFS): 1986-2018 | NHS (N=74563), HPFS (N=39501)/ Total mortality cases= 48373, CVD mortality cases= 11416 (23.5%) | Population based cohort study | 131-item semiquantitative FFQ. UPF intake was measured as servings per day and divided into 4 categories based on NOVA classification | UPF consumption | Risk of CVD mortality | CVD death events ascertained by checking the national health records and were defined with following CVD codes (ICD-8 codes 390-459) |
| 7. | Gauci et al., 2025^120^; Australia | 1990-1994  Follow-up: 2019 | N=39544/CVD mortality cases, n=4229 (10.6%) | Population based cohort study | 121-item semi-quantitative FFQ  UPF was categorised into 4 categorises according to the NOVA classification. The mean daily intake of ultra-processed foods was determined  in grams. | UPF consumption | Risk of CVD mortality | Linkage through national and regional health databases, and CVD-related deaths were coded by the Australian Bureau of Statistics and defined based on underlying cause of death codes from the 9th and 10th revisions of the International Classification of Diseases (390–459 and I00–I99) |
| 8. | Juul et al., 2021^108^; USA | 1991-2008  Follow up:2017 | N=3003/ CVD mortality cases, n=108 (3.5%) | Population-based cohort study | 131-item semi-quantitative FFQ. NOVA was classified into 4 categories. UPF intake for participants was calculated using energy- adjusted daily intakes (servings per day) of foods within each NOVA processing level, as well as energy-adjusted weekly intakes (servings per week) of specific ultra-processed food groups (e.g., sugars-sweetened beverages), using the residual method | UPF consumption | Risk of CVD mortality | CVD mortality cases were retrieved through the hospital and clinic records. |
| 9. | Kermani-Alghoraishi et al., 2024 ^121^; Iran | 2001, follow-up: 2017 | N=5432/CVD mortality cases, n=181 (3.3%) | Prospective cohort study | 48-item FFQ.. UPF intake was estimated on weekly basis, the amounts reported on a daily or monthly basis were multiplied by 7 or divided by 4, respectively. | UPF consumption | Risk of CVD mortality | CVD mortality cases were followed up through health registries and by contacting the participants of the study through telephonic interviews. |
| 10. | Kityo et al., 2023 ^122^; South Korea | 2004-2013 | N=113,576/CVD mortality cases, n=539 (0.47%) | Prospective cohort study | 106-item semi-quantitative FFQ. UPF were defined using the NOVA classification and evaluated as quartiles of the proportion of UPF in the diet (% total food weight). | UPF consumption | Risk of CVD mortality | CVD mortality cases were reterived from the national health registries and were classified as ICD-10 code I00-I99. |
| 11. | Wang et al., 2025 ^123^; UK | 1991-2004  Follow-up: 2019-2021 | N=7138/CVD mortality cases, n=859 (12%) | Prospective cohort study | 127-item semi-quantitative FFQ. NOVA classification was used to identify the FFQ items that fell into the UPF categories. The proportion of UPF was calculated in each participant’s diet by dividing the total amount of UPF consumed per day (in grams) by the total amount of all foods consumed per day (in grams) and then multiplying the resulting ratio by 100. | UPF consumption | Risk of CVD mortality | CVD mortality was sourced from the national health registries through data linkages and were coded according to the International Classification of Diseases, 9th and 10th Revisions (ICD-9 and ICD-10). |
| 12. | Zhao et al., 2024 ^125^; UK and USA | 1. NHANES (1999-2018) 2. UK Biobank (2006-2010)   PLCO screening trial (1993-2001) | 1. NHANES, N=41,070/CVD mortality cases, n=1749 (4.2%) 2. UK Biobank, N=208,051/CVD mortality cases, n=1979 (0.9%) 3. PLCO screening trial, N=108,714/CVD mortality cases, n=8099 (7.4%)   Pooled analyses were conducted. | Prospective cohort study | 24-hr dietary recalls were used. UPF was categorised according to NOVA classification. UPF consumption was calculated as the weight proportion of UPFs in total foods. | UPF consumption | Risk of CVD mortality | CVD mortality was defined as per the ICD-9 code and was retrieved from health registries. |
| 13. | Zhong et al., 2021^124^; USA | PLCO trial (1993-2001) | N=91,891/ CVD mortality cases, n=5490 (5.9%) | Prospective cohort study | 65-item FFQ, were categorised as per the NOVA classification and was summed together to calculate an individual’s overall consumption of ultra-processed foods.  The energy provided by each food was estimated by dividing the amount consumed in grams by 100 and then multiplying the corresponding energy value (kcal) per 100 g of food, which was from the USDA Food and Nutrient Database for Dietary Studies 2015–2016. | UPF consumption | Risk of CVD mortality | The International Classification of Diseases, ninth Revision (ICD-9) was used to define the underlying causes of mortality obtained from death certificates: CVD (codes: 390–459), heart disease (codes 390–398, 402, 404, and 410–429). |
| **INCIDENT CVD** | | | | | | | | |
| 1. | Du et al., 2021^110^; USA | Atherosclerosis Risk in Communities study  1987-2019 | N= 13,548/  ICAD cases, n=2006 (14.8%) | Community-based prospective cohort study | 66-item semi quantitative  FFQ. Foods were categorised into 4 classes according to NOVA classification to extract the UPF. Participants were divided into quartiles based on their  intake of ultra-processed food consumption at baseline | UPF intake | Risk of ICAD | CAD incidence was defined as the first occurrence of a definite or probable hospitalization due to myocardial infarction (MI) or definite CAD death. The ARIC Morbidity and  Mortality Classification Committee reviewed and validated all potential  clinical CAD diagnoses using published criteria and decided upon the final classification |
| 2. | Jalali et al., 2024^113^; Iran | Tehran lipid  and glucose study  1999-2019 | N=2050/ ICVD cases, n=208 (10.1%) | Community-based prospective cohort study | 168-item semi-quantitaive FFQ. Foods were categorised into 4 classes according to NOVA classification to extract the UPF. Participants were divided into tertiles based on their  intake of ultra-processed food consumption at baseline | UPF intake | Risk of ICVD | Diagnoses were made according  to the 10th edition of the International Classification of  Diseases codes (ICD-10) and American Heart Association  (AHA) classification for cardiovascular events. |
| 3. | Srour et al., 2019 ^111^; France | NutriNet-Santé cohort, France 2009-18. | N= 105,159/overall CVD cases, n=1409 (1.3%) | Population based cohort study | 3 24-hr recalls and foods were categorised into 4 classes according to NOVA classification to extract the UPF. | UPF intake | Risk of CVD | Cases were ascertained using ICD-CM codes and via data health linkages |
| 4. | Mendoza et al., 2024 ^112^; USA | The Nurses’  Health Study (NHS), 1976  The Nurses’  Health Study II (NHSII), 1989  The Health Professionals Follow–  up Study (HPFS), 1986 | The Nurses’  Health Study (NHS), N=121,701/CVD cases, n=8,1446 (6.9%)/CHD cases, n=4622 (3.7%)  The Nurses’  Health Study II (NHSII), N=116,340/ CVD cases, n=1,668(1.4%)/CHD cases, n=889 (0.76%)  The Health Professionals Follow–  up Study (HPFS), N=51529/ CVD cases, n=6686(12.9%)/ CHD cases, n=4890 (9.4%) | Population based cohort study | 131-item semiquantitative FFQ. UPF intake was measured as servings per day and divided into 4 categories based on NOVA classification and total energy consumption from UPF was divided into quintiles | UPF intake | Risk of CVD and CHD | Definite and probable (medical records or  death certificate unavailable) cases were included to ascertain CVD and CHD cases. Also, physicians were blinded  to the participants’ dietary information confirmed or  refuted cases by reviewing medical records. |
| 5. | Juul et al., 2021^108^; USA | 1991-2008  Follow up:2017 | N=3003/ 648 cases of incident CVD; 251 cases of hard CVD; and 163 cases of hard CHD. | Population-based cohort study | 131-item semi-quantitative FFQ. NOVA was classified into 4 categories. UPF intake for participants was calculated using energy- adjusted daily intakes (servings per day) of foods within each NOVA processing level, as well as energy-adjusted weekly intakes (servings per week) of specific ultra-processed food groups (e.g., sugars-sweetened beverages), using the residual method | UPF consumption | Risk of CVD mortality | CVD cases were retrieved through the hospital and clinic records. |
| **HEART FAILURE** | | | | | | | | |
| 1. | Sullivan et al., 2023 ^98^; USA | Chronic Renal Insufficiency Cohort (CRIC) Study (2001) | N=3939/ICVD cases, n=406(10.3%) | Population based cohort study | 124-item semi-quantitative FFQ and diet history. Foods were categorised into 4 classes according to NOVA classification to extract the UPF. For  ultra-processed food consumption at baseline, participants were divided into tertiles based on their energy-adjusted intake of UPF foods. | UPF intake | Risk of ICVD progression | Incident CVD was defined as a composite of myocardial infarction, congestive heart failure, or stroke and were ascertained by self-report of cardiovascular-related hospitalizations, outpatient tests, and interventions every 6 months. |
| 2. | Juul et al., 2021^108^; USA | 1991-2008  Follow up:2017 | N=3003/ overall CVD mortality cases, n=648 (21.5%) | Population-based cohort study | 131-item semi-quantitative FFQ. NOVA was classified into 4 categories. UPF intake for participants was calculated using energy- adjusted  daily intakes (servings per day) of foods  within each NOVA processing level, as well as energy-adjusted  weekly intakes (servings per week) of  specific ultra-processed food groups (e.g., sugars-sweetened beverages), using the residual method | UPF consumption | Risk of overall CVD | Overall CVD cases were retrieved through the hospital and clinic records. Overall CVD (CHD, fatal, ischemic and hemorrhagic stroke, transient ischemic accident, cerebral embolism, other cerebral cardiovascular disease, peripheral artery disease [defined as intermittent claudication] and congestive heart failure [hospitalized or nonhospitalized, diagnosed on basis of examination or physician notes]). |
| 3. | Dehghan et al., 2023 ^109^; India and other countries | 2005-2016 | N=138076/Heart failure cases=1000 (0.72%) | Multinational prospective cohort study | 24 hour recalls and country-specific FFQ.  UPFs intake was considered as a serving of intake per day  rather than a proportion of energy. To examine the association between the consumption  of UPFs and outcomes, participants were grouped based on their intake  into 0 serving/d, <1 serving/d, 1 to <2 servings/d, and >2 servings/d of intake. | UPF consumption | Risk of heart failure | Major CVD events  were collected using standardised case-report forms  and reported based on common definitions by medical physicians |
| **RECURRENT CVD** | | | | | | | | |
| 1. | Bonaccio et al., 2022^115^; Italy | 2005-2010, mortality follow-up till 2018 | n=1171 individuals with history of CVD | Prospective cohort study, general adult population | 188-item FFQ  NOVA classification was used to classify UPF.  To estimate UPF, the amount consumed (g/d) of each food group included in the fourth category of the NOVA classification (a total of 15 food groups and 3 beverages) were summed, and then calculated the proportion (%) of UPF in the total weight of food and beverages consumed (g/d) by creating a weight ratio. | UPF consumption | Risk of CVD mortality | CVD mortality included deaths from diseases of the circulatory system, when the underlying cause of death included ICD-9 codes 390–459. |
| **ARRYTHMIAS** | | | | | | | | |
| 1. | Tu et al., 2023 ^107^; UK | UK biobank  2006-2010 | N=121 300/Atrial fibrillation cases, n=4579 (3.77%) | Population based cohort study | 24-hr dietary recalls, Food were classified as per the NOVA classification. Typical serving sizes and appropriate total energy supply per 100 g or 100ml to each food and beverage item were assigned. The energy contribution of UPF(% of total energy)was calculated by dividing the energy intake of UPF group by the total energy intake of UPF and non-UPF group. | UPF intake | Risk of atrial fibrillation | Atrial fibrillation cases were ascertained by self-report data and data linkage with primary care, and hospital admissions. |
| **CVD RISK FACTORS** | | | | | | | | |
| 1. | Palacios et al., 2023^13^; Spain | PREDIMED-Plus trial  2013-2016 | N=5373 participants  Follow-up: 12 months | Prospective analysis of data collected from a multicentre, ongoing, 6-year, randomized clinical  trial | 143-item semi-quantitaive FFQ. Foods were categorised into 4 classes according to NOVA classification to extract the UPF. The UPF consumption per person  was estimated using the sum of the 36 items classified in the UPF group, and UPF consumption were analysed in quartiles  and per 100 g increments | UPF intake | Cardiometabolic risk among adults with metabolic syndrome | The cardiometabolic factors considered were weight, BMI, waist circumference,  blood pressure, fasting blood glucose, HbA1c, triglycerides,  and cholesterol (total, HDL and LDL). Trained staff obtained anthropometric  measures following standardized protocols at baseline, 6 and 12  months of follow-up visits. Weight was measured without shoes and with light clothing using calibrated scales (precision ±100 g). Height  was measured with stadiometers without shoes or objects on the head, in  compliance with the Frankfort plan (precision ±1 mm).  BMI was calculated as weight in kilograms divided by height in meters squared (kg/  m2). Waist circumference was measured at the midpoint between the  last rib and the upper part of the iliac crest with inextensible tapes situated horizontally to the ground (precision ±1 mm). Blood pressure was measured when seated after 5 min of resting, using a validated semiautomatic  oscillometer (Omron HEM-705CP). Weight, height and waist circumference were measured in duplicate and blood pressure in triplicate.  In addition, blood samples after 8 h of fasting to obtain levels of fasting blood glucose, HbA1c, triglycerides and cholesterol  (total, HDL and LDL) were collected by trained nurses at baseline, 6 and 12 months of follow-up, using standardized laboratory procedures. |
| **HYPERTENSION** | | | | | | | | |
| 1. | Rezende-Alves et al., 2021^67^; Brazil | 2016-2018 | N=1221/Hypertension cases, n=370 (30%) | Prospective cohort study | 144-item semi-quantitative FFQ. After categorising the food according to NOVA classification, the percentage contribution of daily energetic intake (%/d) of each group according to the degree of food processing was obtained by adding the energies of each food group and dividing the result by total energy intake.  The values were divided into quintiles, and the lowest quintile was used as a reference. | UPF intake | Risk of hypertension | The definition of hypertension  was according to the cut-off points proposed by ACC/  AHA (BP ≥ 130/80 mmHg) |
| 2. | Li et al., 2022^68^; China | 1997-2015 | N=15054/Hypertension cases, n=4329 (28.7%) | Prospective cohort study | 24-hr dietary recalls for three consecutive days. Food items were classified according to the NOVA classification and UPF intake for each participant at  each survey was categorized into four levels: non-consumers, 1–49 g/day, 50–99 g/day,  >100 g/day. | UPF intake | Risk of hypertension | Hypertension was defined as  having SBP >140 mmHg and/or DBP > 90 mmHg or having known hypertension. |
| 3. | Monge et al., 2021^69^; Mexico | 2006-2008 | N=64934/Hypertension cases, n=3752 (5.7%) | Prospective cohort study | 140-item semi-quantitative FFQ. Food items were categorised as per the NOVA classification.  To estimate the average daily percentage energy contribution  from each NOVA group, relative to total energy intake, energy intake was summed from each FFQ item by NOVA  group and divided it by the total energy intake. | UPF intake | Risk of hypertension | Hypertension was defined when participants reported having hypertension as a medical diagnosis and were under treatment. |
| 4. | Oladele et al., 2024 ^70^; USA | 2003-2007 | N=5957/hypertension cases, n=2134 (35.8%) | Community-based prospective cohort study | 98-item semiquantitative FFQ. NOVA classification was used to categorise the items consumed accordingly to extract UPF foods. Quartiles of consumption were then created based on the distribution of UPF consumption. | UPF intake | Risk of hypertension | Hypertension was  defined according to the Seventh Report of the Joint National Committee on Prevention, Detection, Evaluation, and Treatment  of High Blood Pressure (≥140/90 mm Hg). |
| 5. | Shim et al., 2022^71^; South Korea | 2016-2018 | N=9188/hypertension cases, n=4199 (45.7%) | Prospective cohort study | Food items reported in a one-day 24-hour recall were categorized on the basis of the NOVA (not an acronym) food classification criteria. UPF consumption was estimated as the contribution to total energy intake. | UPF intake | Risk of hypertension | Elevated BP was defined as systolic BP ≥ 120 mmHg or diastolic BP ≥ 80 mmHg. |
| **TYPE 2 DIABETES** | | | | | | | | |
| 1. | Canhada et al., 2023 ^54^; Brazil | ELSA-Brasil cohort | N=10,202/T2D cases, n=1799 (17.6%) | Prospective cohort study | 114-item semiquantitative FFQ. UPF consumption was expressed in two ways:  first, using mean consumption in grams per day (g/day),  expressed as a mean difference of 150 g/day, which represents  approximately a 10% difference in consumption in  our sample; and second, creating quartiles of consumption  (g/day). We | UPF intake | Risk of type 2 diabetes (T2D) | Diabetes cases was defined as follows: participants who (i) reported a medical diagnosis of diabetes or current use of medication for diabetes, or (ii) had a laboratory measurement reaching the thresholds for fasting plasma glucose (FPG) (≥ 7.0 mmol/L; 126 mg/ dL), 2 h post-load glucose (PG) (≥ 11.1 mmol/L; 200 mg/ dL), or HbA1c (≥ 48 mmol/mol; 6.5%) (WHO 2006; ADA 2014). |
| 2. | Chen et al., 2023^55^; USA | Nurses’ Health Study (NHS) (1998); Nurses’ Health Study II (NHSII) (1999); Health Professional Follow-Up Study (HPFS) (1998) | NHS, N=71,871/T2D cases, n=8,591 (12%)  NHSII, N=87,918/T2D cases, n=7,177 (8.1%  HPFS, N=38,847/T2D cases, n=3,735 (9.6%) | Prospective cohort studies | UPF consumption levels  were determined using the NOVA classification and quantified from 131-item semiquantitative FFQ and energy adjusted-UPF consumption was expressed in quintiles | UPF intake | Risk of type 2 diabetes (T2D) | T2D cases were self-reported and cases were confirmed in accordance with National Diabetes Data Group criteria (19) and the American Diabetes Association criteria  (20). |
| 3. | Cho et al., 2024^56^; South Korea | Korean Genome and Epidemiology Study Ansan–Ansung cohort (2001-2002  Follow-up: 5 years | N=7438/T2D cases, n=1187 (16%) | Prospective cohort study | 103-item semiquantitative FFQ. UPF consumption levels were determined using the NOVA classification and the energy adjusted-UPF consumption was expressed in quartiles. | UPF intake | Risk of type 2 diabetes (T2D) | Incident cases of type 2 diabetes were defined as those who reported a diagnosis of type 2 diabetes, taking diabetic medication, or having measured fasting blood glucose concentrations of >126 mg/dL or HbA1c of >6.5% during the follow-up. |
| 4. | Du et al., 2024^57^; USA | Atherosclerosis Risk in  Communities (ARIC) study, 1987-1989  Follow-up: 21 years | N=13,172/T2D cases, n=4539 (34.4%) | Prospective cohort study | 66-item semiquantitative FFQ. UPF consumption levels were determined using the NOVA classification and the energy adjusted-UPF consumption was expressed in quartiles. | UPF intake | Risk of type 2 diabetes (T2D) | Incident diabetes was defined as either having a fasting glucose ≥7 mmol/l, having a non-fasting glucose ≥11.1 mmol/l, or self-reported physician diagnosis of diabetes or use of glucose-lowering medication. |
| 5. | Duan et al., 2022^58^; The Netherlands | The Lifelines cohort study, 2006-2013  Follow-up: 3.5 years | N=70421/T2D cases, n=1128 (1.6%) | Prospective cohort study | 110-item semiquantitative FFQ. UPF consumption levels were determined using the NOVA classification and proportion (weight ratio, %) of intake of UPF in the total weight of food and beverages  consumed per day was calculated and were expressed in quartiles. | UPF intake | Risk of type 2 diabetes (T2D) | Participants were considered an incident diabetes case if they met one of the following criteria: (1) self-reported newly developed type 2 diabetes since last time they filled out a  questionnaire, (2) fasting glucose ≥ 7.0 mmol/L, or (3)  HbA1c ≥ 48 mmol/mol (6.5%) [27]. |
| 6. | Levy et al., 2021^59^; UK | UK Biobank  2007-2010  Follow-up: 2012-2019 | N=21,475/T2D cases, n=305 (1.4%) | Prospective cohort study | UPF consumption levels  were determined using the NOVA classification and quantified from 24-h dietary recall data and consumption was expressed in quartiles | UPF intake | Risk of type 2 diabetes (T2D) | The identification of diabetes cases was based on self-reported and nurse-interview data |
| 7. | Li et al., 2022^60^; China | China Nutrition and Health Survey 1997–2011  Follow-up: 2015 | N=8,382/T2D cases, n=940 (11.2%) | Prospective cohort study | Food items reported in three 24-hour recall were categorized on the basis of the NOVA food classification criteria. UPF consumption was estimated as the contribution to total energy intake. | UPF intake | Risk of type 2 diabetes (T2D) | The identification of diabetes cases was based on self-reported or if self-reported not been told having diabetes  but blood tests results met the diagnostic criteria (fasting plasma glucose >7.0 mmol/L, HbA1c >48 mmol/mol (equivalent to 6.5%) |
| 8. | Moslehi et al., 2024^63^; Iran | Tehran Lipid and Glucose Study (TLGS)  1999-2008  Follow-up: 2016-2018 | N=2457/prediabetes cases, n=766 (31.1%) | Prospective cohort study | 168-item semi-quantitative FFQ. UPF consumption levels were determined using the NOVA classification and consumption was expressed in tertiles | UPF intake | Risk of prediabetes | Pre-diabetes diagnosis was performed based on the criteria of the American Diabetes Association [25]. Pre-diabetes was defined as impaired fasting glucose (fasting serum glucose 100–125 mg/dl) or /and impaired glucose tolerance (2-hour serum glucose 140–199 mg/ dL). |
| 9. | Srour et al., 2020^61^; France | French NutriNet-Santé cohort, 2009-2019 | N=104707/T2D cases, n=821 (0.78%) | Prospective cohort study | UPF consumption levels  were determined using the NOVA classification and quantified from 3 non-consecutive, 24-h dietary recall data and consumption was expressed as 10% increments | UPF intake | Risk of type 2 diabetes (T2D) | T2D case ascertainment was according to (International Statistical Classification of Diseases and Related Health Problems, Tenth Revision) [ICD-10] code E11 |
| 10. | Llavero-Valero et al., 2021^62^; Spain | The SUN project (Seguimiento Universidad de Navarra), 1999-2018  Follow-up: 12 years | N=20060/T2D cases, n=175 (0.87%) | Prospective cohort study | 136-item semi-quantitative FFQ. UPF consumption levels were determined using the NOVA classification and consumption was expressed in tertiles | UPF intake | Risk of type 2 diabetes (T2D) | Incident T2D diabetes diagnosis was via retrieving the medical records and according to American Diabetes Association (ADA) criteria |
| **OBESITY** | | | | | | | | |
| 1. | Cordova et al., 2021 ^44^; France | EPIC study  (multinational cohort study, including France, Italy, Spain, UK, Denmark, Greece, The Netherlands, Norway, Sweden, and Germany)  1992-2000 | N=348,748  Two body weights recorded at baseline and follow-up after 5 years  Overweight or obese, n=191,255 (54.8%)  Obese, n=103259 (29.6%) | Multinational prospective cohort study | FFQs, 7-day and 14-day food diaries were implemented across different countries.  NOVA classification was used to categorise the food items to define them as UPF.  Habitual consumption of energy-adjusted UPFs was modelled  both on a continuous scale per 1 standard deviation (SD)/day  increment (corresponding to ~ 250 g/day) and by categories, where energy-adjusted UPF consumption was divided into quintiles and the lowest consumption quintile was used as reference category. | UPF intake | Risk of becoming overweight or obese | All centers used standardized procedures to measure weight and height at baseline, except, in France, Norway, and Oxford, where subjects self-reported their weight.  The accuracy of self-reported  anthropometric measures at baseline and at follow-up  was improved with prediction equations derived from subjects  with both measured and self-reported weight at baseline. Weight change in kg per 5  years, calculated as weight at follow-up minus weight at baseline divided by the follow-up time in years and multiplied by 5 years. |
| 2. | Beslay et al.,2020 ^46^; France | French  NutriNet-  Santè cohort, 2009-2019 | N=110,390  Obese, n=3,066 (2.7%)  Overweight, n=7,603 (6.3%) | Prospective cohort study | 3 non-consecutive web-based 24-hr dietary recalls. NOVA classification was used to categorise the food items to define them as UPF. | UPF intake | Risk of becoming overweight or obese | Self-reported weight and height were collected using a web-based questionnaire at baseline, and every 6 months thereafter between May 2009 and June 2019, and were used to compute repeated data of BMI (BMI = (weight [kilograms] / height^2^ [meters]). Obesity was identified using international standards as a BMI > 30 kg/m^2^ and overweight  including obesity was identified as a BMI � 25 kg/m2 |
| 3. | Mendonça et al., 2016 ^45^; Spain | SUN cohort | N=8,451, overweight and obese cases, n=1939 (22.9%) | Prospective cohort study | 136-item semiquantutative FFQ. Foods were categorised into 4 classes according to NOVA classification to extract the UPF. Participants were divided into quartiles based on their  intake of ultra-processed food consumption at baseline | UPF intake | Risk of becoming overweight or obese | Self-reported weight and height were validated with a previous  study. The outcome used the incidence of overweight and obesity (BMI >25) during follow-up and was defined as the first-time participants reached a BMI of 25 during follow-up. |
| 4. | Sandoval-Insausti et al., 2020 ^47^; Spain | Seniors-ENRICA-1 cohort: 2008-2010 | N=652, abdominal obesity cases, n=177 (27.1%) | Prospective cohort study | Dietary history. Foods were categorised into 4 classes according to NOVA classification to extract the UPF. | UPF intake | Risk of abdominal obesity | Incident AO was calculated  according to WHO criteria as waist circumference >102 cm in men and >88 cm in women in 2015, among those without AO at baseline |
| 5. | Li et al., 2021 ^47^; China | China Nutrition and Health  Survey (CNHS); 1997-2011  Follow-up: 10 years | N=12,451, overweight, n=5027 (40%); obese, n=2816 (22.6%) | Prospective cohort study | Food items reported in three 24-hour recall were categorized on the basis of the NOVA food classification criteria. UPF consumption was estimated as the contribution to total energy intake. | UPF intake | Risk of becoming overweight or obese | Based on WHO definitions, overweight/obesity was  defined as BMI >25 kg/m^2^ |
| 6. | Rauber et al., 2020 ^48^ ; UK | UK National Diet and Nutrition Survey (NDNS); 2008–16  5 year follow-up | N=22659/ obese, n=6143 (27.1%) | Prospective cohort study | 24 h dietary recall and foods were categorized based on the NOVA food classification criteria. UPF consumption was estimated as the % contribution to total energy intake. | UPF intake | Risk of becoming obese | Based on WHO definitions, obesity was  defined as BMI >30 kg/m^2^ |
| **CHRONIC KIDNEY DISEASE** | | | | | | | | |
| 1. | Cai et al., 2022^99^; The Netherlands | The Lifelines Cohort: 2006-2019 | N=78346/CKD cases, n=2470 (3.1%) | Prospective cohort study | 110-item FFQ, food categorized as per NOVA classification, and the total UPF consumption  (in grams/day) was evaluated by the sum of the food items  considered as UPFs. The weight ratio was used to estimate the  proportion (in grams/day) of UPF consumption from the total  weight of the diet. | UPF intake | Risk of CKD | Composite kidney outcome  including a ≥ 30% eGFR decline relative to baseline or  incident CKD, defined as a de novo occurrence of an eGFR <60 mL/min/1.73 m2 at the second study visit compared with the baseline visit. |
| 2. | Du et al., 2022^100^; USA | Atherosclerosis Risk in Communities (ARIC) study: 1987-1989 | N=14679/CKD cases, n=4859 (33%) | Prospective cohort study | 66-item semi quantitative FFQ. Foods were categorised into 4 classes according to NOVA classification to extract the UPF. Within each  NOVA category, the total daily consumption  for each participant (servings per day), adjusted for energy was calculated. | UPF intake | Risk of CKD | Incident CKD was defined by meeting at least 1 of the  following 4 criteria over a total follow-up period of 32  years from the baseline visit (1987-1989) to the end of  follow-up (December 31, 2018): (1) reduced kidney  function (estimated glomerular filtration rate [eGFR]  < 60 mL/min/1.73 m2) accompanied by ≥25% eGFR  decline at any follow-up study visit relative to baseline;  (2) hospitalization involving CKD stage 3+ diagnosis  defined by International Classification of Diseases (ICD) 9/10 code, identified through active surveillance of the ARIC cohort; (3) death involving CKD stage 3+ diagnosis defined by ICD 9/10 code, identified through linkage to the National Death Index; or (4) kidney failure with  replacement therapy (dialysis or transplantation), identified by linkage to the US Renal Data System (USRDS) registry |
| 3. | Rey-Garcia et al., 2021 ^101^; Spain | Seniors-ENRICA-1 cohort: 2008-2010 | N=1,312/CKD cases, n=183 (13.9%) | Prospective cohort study | Dietary history. Foods were categorised into 4 classes according to NOVA classification to extract the UPF. | UPF intake | Risk of renal function decline | Renal function decline was defined as a SCr  increased or an eGFR decreased beyond that expected for age. Change in eGFR beyond that expected for age was calculated in 3 steps: (i) eGFR based on baseline creatinine and age in 2015; (ii) eGFR in 2015 based on both SCr and eGFR in 2015; and (iii) subtracting ii  from i. |
| 4. | Gu et al., 2023 ^102^; China and UK | UK biobank, 2006-2010 ; Tianjin Chronic Low-Grade Systemic Inflammation and  Health (TCLSIH) cohort study, 2013-2019 | UK biobank, N=102,332/CKD cases, n= 1,740 (1.7%)  TCLSIH, N=23,775/CKD cases, n= 261(1.09%) | Prospective cohort study | 24-hr dietary recalls and FFQ. For each participant, total UPF consumption was calculated using the sum of the UPF items and presented as total gram weight (grams per day) in the TCLSIH and UK Biobank cohorts | UPF intake | Risk of CKD | UK biobank: Incident CKD was ascertained based  on information from medical and death records.  TCLSIH group: CKD was defined as eGFR < 60  mL/min/1.73 m2, albumin-to-creatinine ratio >30 mg/g, or as having a clinical diagnosis of CKD |
| 5. | Sullivan et al., 2023 ^98^; USA | Chronic Renal Insufficiency Cohort (CRIC) Study (2001) | N=3,939/CKD cases, n= 1047 (26.5%) | Population based cohort study | 124-item semi-quantitative FFQ and diet history. Foods were categorised into 4 classes according to NOVA classification to extract the UPF. For  ultra-processed food consumption at baseline, participants were divided into tertiles based on their energy-adjusted intake of UPF foods. | UPF intake | Risk of CKD progression | CKD progression, defined as 50% reduction in eGFR from baseline or initiation of kidney replacement therapy (KRT; dialysis or transplant). |
| **NON-ALCOHOLIC FATTY LIVER (NAFLD)** | | | | | | | | |
| 1. | Fu et al., 2025^90^; South Korean | 2004-2013  Follow-up: 2012, 2016 | N=44,642/NAFLD cases, n=1,562 (3.5%) | Prospective cohort study | 106-item semiquantitative FFQ and food items were then classified according to the NOVA classification.  The proportion (weight) of UPF in the total diet was calculated for each participant and categorised in quartiles. | UPF intake | Risk of NAFLD | NAFLD was diagnosed using the fatty liver index (cut off=60) [14], a well-established index based on four variables, which are triglyceride, BMI, gamma-glutamyl transferase, and waist circumference. |
| 2. | Konieczna et al., 2022^91^; Spain | PREDIMED-Plus trial  2013-2016 | N=5,867/NAFLD cases (HIS >36), n=5,585 (95.2 %) | Longitudinal analyses nested within a randomized controlled trial (RCT) | 143-item semi quantitative FFQ and food items were then classified according to the NOVA classification.  The consumption of UPF was expressed as a percentage of total food and beverage intake in g/day. | UPF intake | Risk of elevated NAFLD-related biomarkers | The fatty liver index (FLI) (values < 30 ruling out and values > 60) and/or hepatic steatosis index (HSI) (> 36) were used as surrogate measures of NAFLD [37,38]. |
| 3. | Zhang et al., 2024^92^; UK | UK Biobank  2006-2010  Follow up: 10 years | N=143,073/NAFLD cases, n=1445 (1%) | Prospective cohort study | UPF consumption levelswere determined using the NOVA classification and quantified from 24-h dietary recall data and consumption was expressed in quartiles | UPF intake | Risk of severe NAFLD | Severe NAFLD was defined based on clinical outcomes, specifically hospitalization or death attributed to NAFLD. The criteria for defining severe NAFLD adhered to the International Classification of Diseases, 10th revision (ICD-10) (K76.0 (fatty liver, not elsewhere classified), and K75.8 (NASH, other specified inflammatory liver diseases)). |
| 4. | Zhao et al., 2024^89^; UK | UK Biobank  2006-2010  Follow-up: 9 years | N=137,173/ NAFLD cases, n=1108 (0.8%) | Prospective cohort study | UPF consumption levels were determined using the NOVA classification and quantified from 24-h dietary recall data and energy adjusted-UPF consumption was expressed in quartiles | UPF intake | Risk of NAFLD | NAFLD diagnosis was extracted from health registries and defined with an ICD-10 code: K75.8 |
| 5. | Garcia et al., 2025^93^, Spain | FLIPAN study; 2018-2020 | N=70 MASLD cases | Longitudinal study analysis, a part of the RCT | 143-item semi-quantitative FFQ was used to extract UPF food items. UPF consumption levels were determined using the NOVA classification and quantified into tertiles (T1 as maximum and T3 as minimum) | UPF intake | Risk of MASLD | Participants were required to be between 40 and 60 years old, be characterized as overweight or obese (with a body mass index of between 27 and 40 kg/m2), be diagnosed with MASLD using magnetic resonance imaging (MRI) to measure intrahepatic fat content (IFC), and meet at least three MetS criteria according to the International Diabetes Federation guidelines |
| **METABOLIC SYNDROME** | | | | | | | | |
| 1. | Pan et al., 2023^82^; China | 2009-2018 | N=5,147/Metabolic syndrome cases, n=1,712 (33.2%) | Prospective cohort study | Food items reported in a one-day 24-hour recall were categorized based on the NOVA food classification criteria. UPF consumption was estimated as the contribution to total energy intake. | UPF intake | Risk of metabolic syndrome | Metabolic syndrome was defined using the National Cholesterol Education Program Adult Treatment  Panel III (NCEP ATP III) criteria in this study. If at least three out of five of the following components were present, the person was determined to have Metabolic syndrome: (1) central obesity:  waist circumference (WC) >90 cm (men) and >80 cm (women); (2) raised triglycerides (TG): >150 mg/dL or relevant specific treatment for hyperlipidemia; (3) reduced high-density lipoprotein cholesterol (HDL-C): < 1.0 mmol/L (men) and 1.3 mmol/L (women); (4) raised blood pressure: systolic blood pressure (SBP) > 130 mmHg or diastolic blood pressure (DBP) > 85 mmHg or specific treatment of previously diagnosed hypertension; (5) raised fasting plasma glucose (FPG): >6.0 mmol/L or diagnosed type 2 diabetes previously |
| 2. | DaSilva  Magalhães et al., 2022 ^83^; Brazil | Ribeirão  Preto cohort, 2016-2017 | N=896/ metabolic syndrome cases, n=317 (35.3%) | Prospective cohort study | 83-item semiquantitative FFQ. UPF consumption was estimated as the % contribution to total energy intake. | UPF intake | Risk of metabolic syndrome | The criterion was considered to be a change in at least three components according to parameters established by the Joint Interim Statement (JIS) which are as follows: WC > 90 cm for men and >80 cm for women; triglycerides >150 mL/dL or use of antilipemic medicine; HDL-c < 40 mg/dL for men and <50 mg/dL for women or use of antilipemic medication; systolic > 130 mmHg or diastolic arterial pressure > 85 mmHg or use of antihypertensive medication; and fasting blood sugar > 100 mg/dL or use of antihyperglycemic medication. |
| **DYSLIPIDEMIA** | | | | | | | | |
| 1. | Scaranni et al., 2023^75^; Brazil | 2008-2010  Follow-up:4 years | N=5275/ Isolated hypercholesterolemia, n=629 (11.9%); isolated hypertriacylglycerolaemia , n=857 (16.2%); mixed hyperlipidaemia, n=458(8.6%); low-HDL, n=842 (15.9%) | Prospective cohort study | 114-item semi-quantitative FFQ were categorized on the basis of the NOVA food classification criteria.  The proportion (weight) of UPF in the total diet was calculated for each participant and categorised in tertiles, corresponding to low (first tertile), medium (second tertile) and high (third tertile) consumption. | UPF intake | Risk of dyslipidaemia (Isolated hypercholesterolaemia, isolated hypertriacylglycerolaemia, mixed hyperlipidaemia, and low-HDL) | Incident cases of dyslipidaemias during the follow-up period  were defined the same way as prevalent cases, that is, according to the criteria from the Updated Brazilian Guidelines on Dyslipidemias and Prevention of Atherosclerosis(27), namely:  Ultra-processed foods and dyslipidaemias 337  https://doi.org/10.1017/S0007114522001131 Published online by Cambridge University Press  isolated hypercholesterolaemia (LDL ≥ 4.12 mmol/L), isolated  hypertriacylglycerolaemia (TAG ≥ 1.69 mmol/L), mixed hyperlipidaemia  (LDL ≥ 4.12 mmol/L associated with TAG ≥ 1.69  mmol/L) and low-HDL (HDL < 1.03 mmol/L for men and  <1.29 mmol/L for women with or without association with  increased LDL and TAG). An additional criterion for incidence  of dyslipidaemias was the use of lipid-lowering drugs as  described above. |
| 2. | Donat-Vargas et al., 2021^76^; Spain | 2008-2010  Follow-up: 2015 | N=1082/ hypertriglyceridemia, n=60 (5.5%); low LDL n=54 (4.9%); low HDL, n=112 (10.3%) | Prospective cohort study | Validated computer-based diet history (DH-ENRICA) was used to extract food items and were then classified according to the NOVA classification.  The proportion (weight) of UPF in the total diet was calculated for each participant and categorised in tertiles, corresponding to low (first tertile), medium (second tertile) and high (third tertile) consumption. | UPF intake | Risk of dyslipidaemia (hypertriacylglycerolaemia, low LDL and low HDL) | The threshold for hypertriglyceridemia was ≥150 mg/dL, the threshold  for low HDL cholesterol was <40 in men or <50 mg/dL in  women, and the threshold for high LDL cholesterol was >129 mg/dL |
| **COMORBIDITIES** | | | | | | | | |
| 1. | Bonaccio et al., 2023^12^; Italy | Moli-Sani cohort  (2005-2010) | N=1065 with T2D at baseline/CVD mortality cases, n=129 (12.1%) | Prospective cohort study | 188-item FFQ  NOVA classification was used to classify UPF.  To estimate UPF the amount consumed (g/d) of each food group included in the fourth category of the NOVA  classification (a total of 15 food groups and 3 beverages) were summed, and  then calculated the proportion (%) of UPF in the total weight of food and beverages consumed (g/d) by creating a weight ratio. | UPF consumption | Risk of CVD mortality among T2D population | CVD mortality included deaths from diseases of the circulatory system, when the underlying cause of death included ICD- 9 codes 390–459, and were assessed by the Italian mortality registry (ReNCaM registry), and validated by Italian death certificates (ISTAT form). |
| 2. | Liu et al.,2023^14^; UK | UK biobank  2006-2010 | N=4058 new CKD cases (with diabetes, n=593 (14.6%)/without diabetes, n=3465 (85.4%)) | Prospective, population based cohort study | 24-hr dietary recalls, Food were classified as per the NOVA classification. Typical serving sizes and appropriate total energy supply per 100 g or 100ml to each food and beverage item were assigned. The energy contribution of UPF(% of total energy)was calculated by dividing the energy intake of UPF group by the total energy intake of UPF and non-UPF group. | UPF consumption | Risk of CKD among T2D population | The date of CKD was ascertained by self-report data and data linkage with primary care, hospital admissions, and death registry records based on the International Classification of Diseases,10^th^ revision (ICD-10) coding system (N18). |
| **RANDOMIZED CONTROLLED TRIALS** | | | | | | | | |
| **OBESITY/WEIGHT CHANGE** | | | | | | | | |
| 1. | Dicken et al., 2025 ^52^; UK | Ultra processed versus minimally processed diets  following UK dietary guidance on health outcomes (UPDATE) trial, 2023-2024 | n=55, randomised for UPF diet=28 and minimally-processed food diet=27 (intention-to-treat sample) | Crossover RCT | 2 24-hr dietary recalls from the MPF and UPF diets were recorded | UPF vs MPF diet (8-weeks/each), with 4-week washout period | % Weight change between diets | Measured at weight management clinics using and self-reported weight and BMI ≥25 kg/m^2^ |
| 2. | Hall et al., 2021 ^50^; USA | Trial composed of inpatients residing in the NIH Clinical Center | N=20 randomised to receive either UPF or MPF diets followed by alternate diet | Crossover RCT | The diets were designed and analyzed using ProNutra software (version 3.4, Viocare, Inc., Princeton, NJ) with nutrient values derived from the USDA National Nutrient Database for Standard Reference, Release 26 and the USDA Food and Nutrient Database for Dietary Studies, 4.0. The ultra-processed and unprocessed meals were provided on 7-day rotating menus. Foods and beverages were categorized according to the NOVA system | UPF vs MPF diet (2-weeks/each), with no washout period | Weight changes in kg after following UPF or MPF diet | Measured at hospitals using scale weight |
| 3. | Hamano et al., 2024 ^51^; Japan | Trial composed of inpatients residing in the University of Tokyo Hospital | N=9 randomised to receive either UPF or MPF diets followed by alternate diet | Crossover RCT | The diets were designed and analysed using Asken Diet software  (Asken Corporation, Tokyo, Japan) with nutrient values derived from  the Dietary Intake Standards for Japanese | UPF vs MPF diet (1 week/each), with 2-week washout period | Weight gain (in kgs) when consumed UPF diet as compared to MPF diet | Measured at hospitals using scale weight and participants having BMI ≥25 kg/m^2^ were included |

**Supplementary table 6: Results of the included studies exploring the association between ultra-processed foods in lifestyle management and cardiovascular diseases.**

| **PROSPECTIVE STUDIES** | | | | | | | |
| --- | --- | --- | --- | --- | --- | --- | --- |
| **CARDIOVASCULAR MORTALITY** | | | | | | | |
| **Serial number** | | **Author, Year** | **Outcome** | **Covariates** | **Statistical methods** | **Results** | **Conclusion** |
| 1. | | Bonaccio et al., 2021^116^ | Risk of CVD mortality | Sex, age (continuous), energy intake (continuous), educational level (categorical), housing tenure (categorical), smoking (categorical), BMI (continuous), leisure-time physical activity (continuous), history of cancer, CVD, diabetes, hypertension, hyperlipidemia, and residence (categorical), and Mediterranean Diet Score (continuous). | Risk estimates for cause-specific deaths were expressed as HRs with 95% CIs and calculated by using Cox proportional hazards models with time-on-study on the time scale and adjusting for baseline age as a covariate in the model  Multivariable-adjusted HRs to explore associations between quartiles of UPF and risk of CVD mortality | CVD mortality:  Individuals reporting the highest intake of UPF (Q4, >14.6% of total food), as opposed to the lowest (Q1, UPF < 6.6%), experienced increased risks of CVD mortality (HR: 1.58; 95% CI: 1.23, 2.03). | A high proportion of UPF in the diet was associated with increased risk of CVD mortality. |
| 2. | | Bonaccio et al., 2022^115^ | Risk of CVD mortality | Sex, age (continuous), energy intake (continuous), educational level (categorical), housing tenure (categorical), smoking (categorical), body mass index (continuous), leisure-time physical activity (continuous), history of cancer (no/yes), diabetes (no/yes), hypertension (no/yes), hyperlipidaemia (no/yes), and residence (categorical) and Mediterranean diet score (continuous). | Multivariable Cox regression analysis to explore relationship between UPF and CVD mortality | Cox analyses, higher intake of UPF (Q4,>_11.3% of total food), as opposed to the lowest (Q1, UPF <4.7%), was associated with higher hazards of CVD mortality (HR: 1.65; 95% CI: 1.07–2.55). | A high proportion of UPF in the diet was associated with an increased hazard of CVD mortality. |
| 3. | | Chen et al., 2022^117^ | Risk of CVD mortality | Age, sex, ethnicity, education years, smoking status, Townsend deprivation index, obesity status, sleep duration, total energy intake, physical activity, protein, total fat, carbohydrates, alcohol, fibre, saturated fat, monounsaturated fat, polyunsaturated fat, trans-fat, hypertension, diabetes and dyslipidaemia. | Multivariable Cox proportional hazards analysis to examine UPF consumption and CVD mortality | A higher intake of UPF was associated with a higher risk  of CVD mortality [hazard ratio (HR)(1.17, 95% CI: 1.09–1.26) | A higher proportion of UPF consumption was associated with CVD mortality. |
| 4. | | Torres-Collado et al., 2024^118^ | Risk of CVD mortality | Sociodemographic, medical, and lifestyle information was collected at baseline using structured and validated questionnaires:  sex (women, men); age (years); educational level (<primary school,  primary school, secondary school); waist circumference [healthy  range (78-94 cm in men and 64-80 cm in women), moderate risk  (94-102 cm in men and 80-88 cm in women) and increased risk  (>102 cm in men and >88 cm in women)]; smoking habit (never,  ex-smoker, current); television (TV) watching (hours/day); sleep  (hours/day) and pre-existing self-reported diabetes and hypertension  at baseline (yes/no). | Multivariable Cox proportional hazards analysis to examine UPF consumption and CVD mortality | Compared with participants in the lowest tertile of UPF consumption, those in the highest tertile showed a higher risk CVD mortality, HR 1.39 (95 %CI: 0.80-2.41) | A high UPF consumption was associated with a higher CVD mortality. |
| 5. | | Dehghan et al., 2023^109^ | Risk of CVD mortality | Age, sex, urban/rural location, education, wealth index, country income level, smoking, body mass index, physical activity, history of diabetes, history of cancer, history of hypertension, blood pressure medication, daily energy intake, and country as random effect. | Multivariable Cox proportional hazards analysis to examine UPF consumption and CVD mortality | A diet high in UPFs (>2  servings/d compared with 0 intake) was associated with high risk of CVD mortality (HR: 1.17; 95% CI: 0.98, 1.41; p-trend = 0.04) | A diet with a high intake of UPFs was associated with a higher risk of CVD mortality |
| 6. | | Fang et al., 2024^119^ | Risk of CVD mortality | Race, marital status, physical activity, body mass index, smoking status and pack years, alcohol consumption, physical examination  performed for screening purposes, and family history of diabetes mellitus, myocardial infarction, or cancer; for women, also menopausal status and hormone use. | Multivariable Cox proportional hazards analysis to examine UPF consumption and CVD mortality | No associations were found between UPF consumption and cardiovascular mortality (HR: 1.04; 95% CI: 0.99, 1.10; p-trend = 0.14) | A diet with a high intake of UPFs was not associated with risk of CVD mortality |
| 7. | | Gauci et al., 2025^120^ | Risk of CVD mortality | Sex (male, female), age (continuous), education (above, below high school), country of birth (Australia/New Zealand, Southern Europe, Northern Europe), marital status (married or in a relationship, not married or single), number of people occupying household (1, 2, 3–4, 5+), and SEIFA quintiles (Q1–Q5), lifestyle and health-related behaviours: smoking status (never smoked, current smoker, former smoker), physical activity over the last 6 months (0 [none], >0 and <4 [low], ≥4 and <6 [moderate], ≥6 [high]), and alcohol intake (g/d) (lifetime abstainers, ex-drinkers, up to 19, 20–29, 30–39, 40+). | Multivariable Cox proportional hazards analysis to examine UPF consumption and CVD mortality | Highest relative intake of ultra-processed food had higher risk of cardiovascular mortality (HR high (quartile 4) vs. low (quartile 1) category = 1.19, 95% confidence intervals: 1.09–1.29, P-value for trend < 0.001). | A higher exposure to the ultra-processed food pattern was  associated with a higher risk of CVD mortality. |
| 8. | | Juul et al., 2021^108^ | Risk of CVD mortality | Age (continuous), sex, education (categorized as 12 years or less, 13 to 15 years and 16+ years), smoking status (never-smoker, current smoker, former smoker), alcohol intake (g/d), and physical activity (continuous) as time-varying covariates, baseline total energy intake, diet quality defined by the DGAI-2010 and waist circumference. | Multivariable Cox proportional hazards analysis to examine UPF consumption and CVD mortality | Higher ultra-processed  food intake was associated with increased risk  CVD mortality (multivariable-adjusted HR: 1.09; 95% CI: 1.02 to 1.16) | A diet with a high intake of UPFs was associated with increased risk of CVD mortality |
| 9. | | Kermani-Alghoraishi et al., 2024^121^ | Risk of CVD mortality | Sex, marital status, education, smoking, physical activity, BMI, diabetes, dyslipidemia, HTN, and food groups including fruit and  vegetables, beans, soy, chicken and fish, ghee, animal fats or butter, meat, egg, high-fat dairy products, and oils (non-hydrogenated and olive). | Multivariable Cox proportional hazards analysis to examine UPF consumption and CVD mortality | UPF consumption was not associated with CVD mortality (HR= 0.95, 95% CI: 0.61,1.47; P for trend=0.596). | No association between high UPF consumption and CVD mortality |
| 10. | | Kityo et al., 2023^122^ | Risk of CVD mortality | Age, total energy intake, education level, monthly income, marital status, smoking, drinking, regular physical exercise, BMI, disease score, menopausal status, use of oral contraceptives and hypercholesterolemia (for CVD mortality). | Multivariable Cox proportional hazards analysis to examine UPF consumption and CVD mortality | There was no evidence  of an association between CVD mortality and UPF intake (comparing the highest with the lowest quartiles of UPF intake) (men: HR 0.88, 95% CI 0.64–1.22; women: HR 0.80, 95% CI 0.53–1.19). | No association between high UPF consumption and CVD mortality |
| 11. | | Wang et al., 2025^123^ | Risk of CVD mortality | Age, gender, ethnicity, education level, employment grade, smoking, alcohol, physical activity levels, total energy intake (kcal), nutritional indicators of diet quality including total sodium intake (mg/day), sugar (g/day), and fat (g/day), BMI, hypertension, T2D, and dyslipidemia. | Multivariable Cox proportional hazards analysis to examine UPF consumption and CVD mortality | No significant associations were observed between UPF intake trajectory groups and the risk of CVD mortality (moderate UPF intake: HR 1.15, 95% CI 0.75 to 1.77; high UPF intake: HR 1.07, 95% CI 0.68 to 1.66). | No significant associations were observed between UPF trajectory groups  and CVD mortality. |
| 12. | | Zhao et al., 2024^125^ | Risk of CVD mortality | Age,sex,race,smoking status,alcohol drinking status,education,body mass index(BMI),total energy intake,history of hyper-tension, diabetes, CVD, and cancer (UK Biobank and NHANES), randomization arm (PLCO),marital status  (PLCO and NHANES), family income and physical activity (UK Biobank and NHANES), sleep duration and Townsend deprivation index(UK Biobank). | Multivariable Cox proportional hazards analysis to examine UPF consumption and CVD mortality (pooled analyses) | Combined analyses of the three cohorts showed that those with the highest quartile of UPF consumption had higher risk of CVD mortality (HR,1.17; 95% CI, 1.06−1.28) compared to the lowest  quartile of UPF consumption. | Higher UPF consumption was associated with increased CVD mortality risk. |
| 13. | | Zhong et al., 2021^124^ | Risk of CVD mortality | Age (years), sex (male, female), race (non-Hispanic white, non-Hispanic black, Hispanic, others), educational level (college below, college graduate, postgraduate), marital status (married, widowed, divorced, separated, never married), and study center (10 categories), aspirin use (yes, no), history of hypertension (yes, no), history of diabetes (yes, no), smoking status (current, former, never), alcohol consumption (g/day), body mass index (< 18.5, 18.5–24.9, 25.0–30.0, > 30.0 kg/m2), physical activity (min/week), and energy intake from diet (kcal/day) | Multivariable Cox proportional hazards analysis to examine UPF consumption and CVD mortality | Participants in the highest vs. the lowest quintiles of UPF consumption were found to be at increased risks of overall CVD mortality (HR quintile 5 vs. 1, 1.50; 95% CI, 1.36–1.64; P trend < 0.001) | Higher UPF consumption was associated with increased CVD mortality risk. |
| **INCIDENT CVD** | | | | | | | |
| 1. | | Du et al., 2021^110^ | Risk of ICAD | Age, sex, race–center, total energy intake, education level, smoking status, drinking status, and physical activity score. | Cox proportional hazards models and restricted cubic splines used to assess the association between quartiles of ultra-processed food intake and incident coronary artery disease. | Participants in the highest compared with lowest quartile of ultra-processed food intake had a higher risk of coronary artery disease (HR: 1.19; 95% CI: 1.05, 1.35) | Higher ultra-processed food intake was associated with a higher risk of coronary artery disease. |
| 2. | | Jalali et al., 2024^113^ | Risk of ICVD | CVD-risk score, physical activity, total energy intakes (kcal/d), and dietary intakes of fiber (g/d) | Multivariable-adjusted Cox proportional hazards models were used to assess the association between UPF consumption and ICVD. | Participants with the highest intake of UPF had a higher incidence of CVD compared to those with the lowest intake (HR = 1.68, 95% CI=1.14–2.48). | Higher ultra-processed food intake was associated with a higher incidence risk of CVD. |
| 3. | | Srour et al., 2019 ^111^ | Risk of CVD | Sex, energy intake, number of 24-hour dietary records, smoking status, educational level, physical activity, body mass index, alcohol intake, family history of cardiovascular disease, saturated fatty acid intake, sodium intake, sugar intake, healthy dietary pattern, intakes of sugary products, red and processed meat, salty snacks, beverages, and fats and sauces, baseline prevalent type 2 diabetes, dyslipidemia, hypertension, and hypertriglyceridemia (yes or no) as well as treatments for these conditions (yes or no). | Multivariable-adjusted Cox proportional hazards models were used to assess the association between UPF consumption and risk of CVD | UPF intake (10%increment) was associated with a higher risk of overall CVD risk (HR=1.12 (95% CI 1.05, 1.20) | Higher ultra-processed food intake was associated with a higher risk of CVD |
| 4. | | Mendoza et al., 2024 ^112^ | Risk of CVD and CHD | Sex, race/ethnicity, marital status, working status, smoking status, quintiles of physical activity (MET-hours/week), sleep patterns (hours/day), family history of CVD, multivitamin use, aspirin use, NSAID use, menopausal hormone use status (women only), oral contraceptive use (women only), energy intake, BMI at baseline, hypertension at baseline, hypercholesterolemia at baseline, and diabetes at baseline. | Multivariable-adjusted Cox proportional hazards models were used to assess the association between UPF consumption and risk of CVD and CHD | High ultra-processed food consumption was associated with a significant increase in CVD and CHD risks (Q5 vs Q1)  NHS:  CVD risk (HR=1.05 (95% CI 0.98,1.13)) and CHD (HR=1.11 (95% CI 1.01,1.22)),    NHSII:  CVD risk (HR=1.22 (95% CI 1.05,1.42)) and CHD risk (HR=1.28 (95% CI 1.04,1.56))    HPFS:  CVD risk (HR=1.15 (95% CI 1.07,1.24)) and CHD risk (HR=1.19 (95% CI 1.09,1.30)) | Higher ultra-processed food intake was associated with a higher risk of CVD and CHD |
| 5 | | Juul et al., 2021^108^ | Risk of hard CVD | Age (continuous), sex, education (categorized as 12 years or less, 13 to 15 years and 16+ years), smoking status (never-smoker, current smoker, former smoker), alcohol intake (g/d), and physical activity (continuous) as time-varying covariates, baseline total energy intake, diet quality defined by the DGAI-2010 and waist circumference. | Multivariable Cox proportional hazards analysis to examine UPF consumption and CVD mortality | Higher ultra-processed  food intake was associated with increased risk  of   - hard CVD (HR: 1.07; 95%CI 1.03–1.12) - hard CHD (HR: 1.09; 95%CI 1.04-1.15) - Overall CVD (HR: 1.05; 95%CI 1.02-1.08) | A diet with a high intake of UPFs was associated with increased risk of hard CVD |
| **HEART FAILURE** | | | | | | | |
| 1. | | Sullivan et al., 2023 ^98^ | Risk of ICVD | Age, sex, total energy intake, race/ethnicity, education, income, smoking status, physical activity, and study site | Multivariable-adjusted Cox proportional hazards models were used to assess the association between UPF consumption and ICVD | High UPF intake was not associated with risk of ICVD (Q3 vs Q1 unadjusted HR=1.09; 95%CI 0.85, 1.40). | Higher UPF consumption was not associated with a higher risk of ICVD, including congestive heart failure |
| 2. | | Juul et al., 2021^108^ | Risk of overall CVD | Age (continuous), sex, education (categorized as 12 years or less, 13 to 15 years and 16+ years), smoking status (never-smoker, current smoker, former smoker), alcohol intake (g/d), and physical activity (continuous) as time-varying covariates, baseline total energy intake, diet quality defined by the DGAI-2010 and waist circumference. | Multivariable Cox proportional hazards analysis to examine UPF consumption and overall CVD | Higher ultra-processed  food intake was associated with increased risk of  overall CVD (multivariable-adjusted HR: 1.05; 95% CI: 1.02 to 1.08) | A diet with a high intake of UPFs was associated with increased risk of overall CVD including congestive heart failure |
| 3. | | Dehghan et al., 2023^109^ | Risk of heart failure | Age, sex, urban/rural location, education, wealth index, country income level, smoking, body mass index, physical activity, history of diabetes, history of cancer, history of hypertension, blood pressure medication, daily energy intake, and country as random effect. | Multivariable Cox proportional hazards analysis to examine UPF consumption and heart failure | A diet high in UPFs (>2  servings/d compared with 0 intake) was not associated with high risk of heart failure (HR: 0.22; 95% CI: 0.89, 1.68) | A diet with a high intake of UPFs was not associated with a higher risk of heart failure |
| **RECURRENT CVD** | | | | | | | |
| 2. | | Bonaccio et al., 2022^115^ | Risk of CVD mortality | Sex, age (continuous), energy intake (continuous), educational level (categorical), housing tenure (categorical), smoking (categorical), body mass index (continuous), leisure-time physical activity (continuous), history of cancer (no/yes), diabetes (no/yes), hypertension (no/yes), hyperlipidaemia (no/yes), and residence (categorical) and Mediterranean diet score (continuous). | Multivariable Cox regression analysis to explore relationship between UPF and CVD mortality | Cox analyses, higher intake of UPF (Q4,>_11.3% of total food), as opposed to the lowest (Q1, UPF <4.7%), was associated with higher hazards of CVD mortality (HR: 1.65; 95% CI: 1.07–2.55). | A high proportion of UPF in the diet was associated with an increased hazard of CVD mortality. |
| **ATRIAL FIBRILLATION** | | | | | | | |
| 1. | | Tu et al., 2023 ^107^ | Risk of atrial fibrillation | Sex and assessment centre attended, and adjusted for age, Townsend deprivation index, ethnicity, education, total energy intake, body mass index (BMI), smoking status, total metabolic equivalent of task minutes/week, alcohol consumption status, alcohol consumption amount, and comorbidities | Multivariable-adjusted Cox proportional hazards models were used to assess the association between UPF consumption and risk of atrial fibrillation | High ultra-processed food consumption was associated with a significant increase in AF risk (HR=1.13 (95% CI 1.02,1.24)) | Higher ultra-processed food intake was associated with a higher risk of atrial fibrillation |
| **CVD RISK FACTORS** | | | | | | | |
| 1. | Palacios et al., 2023^13^ | | Cardiometabolic risk among adults with metabolic syndrome | Age, sex, intervention group, follow-up time, educational level, smoking status, physical activity, energy-restricted Mediterranean diet adherence and sedentary time. | Multivariable-adjusted mixed-effects linear models used to assess the associations between UPF consumption and changes in cardiometabolic factors | Compared with the lower quartile, the highest quartile of UPF consumption was positively associated with weight (kg, β = 1.09; 95%CI = 0.91, 1.26), BMI (kg/m2, β = 0.0.39; 95% CI = 0.33, 0.46), waist circumference (cm, β = 1.03; 95%CI = 0.81, 1.26), diastolic blood pressure (mm Hg,β = 0.67; 95%CI = 0.29,1.06), fasting blood glucose (mg/dl, β = 1.66; 95%CI = 0.61, 2.70), HbA1c (%,β = 0.04; 95%CI = 0.01, 0.07), and plasma triglycerides (mg/dl, β = 6.79; 95% CI = 3.66, 9.91). | Higher UPF consumption was associated with adverse evolution in objectively measured CMR factors (including weight, waist circumference, BMI, diastolic blood pressure, fasting blood glucose and plasma triglycerides), but not with systolic blood pressure, cholesterol classes such as total, HDL and LDL) among adults with metabolic syndrome. |
| **HYPERTENSION** | | | | | | | |
| 1. | Rezende-Alves et al., 2021^67^ | | Risk of hypertension | Gender, age, marital status, skin colour, per capita income, physical activity, smoking, obesity, family history of hypertension, alcohol consumption, previous diagnosis of type 2 diabetes, hypercholesterolemia and hypertriglyceridemia. | Multivariable-adjusted regression used to assess the associations between UPF consumption and risk of hypertension | Participants in the upper quintile of UPF intake were associated with a reduced risk of hypertension  (RR: 1.35; 95 % CI 1.01, 1.81) | Highest consumption of UPF was associated with increased risk of hypertension |
| 2. | | Li et al., 2022^68^ | Risk of hypertension | Age, sex, energy intake, income, education, urbanization,  smoking, alcohol drinking, physical activity, sleep duration, BMI, sodium/potassium intake, fruit, vegetables/tea intakes and diabetes. | Cox regression to explore the association between UPF intake and risk of hypertension | Compared with non-consumers, the hazard ratios (95% CI) for UPF intake of >100 g/day were (HR=1.20, 95% CI 1.06–1.35, p = 0.001) | UPF consumption was associated with increased risk of hypertension |
| 3. | | Monge et al., 2021^69^ | Risk of hypertension | Age, indigenous (yes/no), internet access (yes/no), insurance (private, social, other), family history of hypertension (yes/no), menopausal status (premenopausal, postmenopausal, unknown), smoking (never, past, current and missing) physical activity (tertiles), energy intake (continuous) and multivitamin intake (yes/no). | Poisson regression models to estimate the association between UPF intake and risk of hypertension | Comparing extreme categories showed that higher total UPF consumptions were not associated with incident hypertension (RR=0.96, 95% CI 0.79-1.16) | UPF consumption was not associated with increased risk of hypertension |
| 4. | | Oladele et al., 2024^70^ | Risk of hypertension | UPF calorie percentage rank, total calories, age, sex, education, income,  smoking, physical activity, alcohol, BMI, and healthy eating index.  UPF indicates ultra-processed food. | Multivariable logistic regression models used to assess the association between UPF intake and the risk of hypertension, analysis stratified by race (black Americans and Caucasians) | Black and White participants in the highest consumption quartile had (OR=1.26, 95% CI, 0.92–1.74) and (OR=1.22, 95% CI, 1.01–1.47) greater odds of hypertension compared with those in the lowest quartile, respectively | High consumption of UPF was associated with increased hypertension risk. |
| 5. | | Shim et al., 2022^71^ | Risk of hypertension | Age, sex, body mass index, income (lower median/upper median), education level (≤6 years, 7–12 years, >12 years), occupation (white, pink, blue, and others), region (rural, urban), smoking status (nonsmoker, ex-smoker, current smoker), exercise (yes, no), total energy intake, history of disease (at least one of dyslipidemia, stroke, myocardial infarction, angina pectoris, diabetes, and kidney failure), and Korean healthy eating index. | Multivariable logistic regression models to assess the association between UPF intake and the risk of hypertension. | Comparing extreme categories showed that higher total UPF consumptions was associated with incident hypertension (OR=1.22, 95% CI 1.09-1.37) | High consumption of UPF was associated with increased hypertension risk. |
| **TYPE 2 DIABETES** | | | | | | | |
| 1. | | Canhada et al., 2023^54^ | Risk of T2D | Age, sex, race/color, income, school achievement, family history of diabetes, smoking, physical activity and alcohol | Multivariable Poisson regression used to analyse the association between UPF intake and risk of T2D | Higher UPF intake was associated with increased risk of T2D (RR Q4 vs. Q1: 1.24; 95% CI: 1.10-1.39) | Higher consumption of UPF was associated with an increased risk of T2D |
| 2. | | Chen et al., 2023^55^ | Risk of T2D | Age, race/ethnicity, family history of diabetes, history of hypercholesterolemia  at baseline, history of hypertension at baseline, smoking status, physical activity, oral contraceptive use, postmenopausal hormone use, physical examination in the past 2 years, neighbourhood income, total energy, total alcohol consumption  and BMI | Cox proportional hazards models used to assess the association between UPF intake and risk of T2D | NHS: Higher UPF intake compared to the lower quintile was associated with higher risk of T2D (HR: 1.19; 95% CI: 1.09–1.30).  NHSII: Higher UPF intake compared to the lower quintile was associated with higher risk of T2D (HR: 1.46; 95% CI: 1.33–1.60).  HPFS: Higher UPF intake compared to the lower quintile was associated with higher risk of T2D (HR: 1.22; 95% CI: 1.07–1.39). | Higher consumption of UPF was associated with an increased risk of T2D |
| 3. | | Cho et al., 2024^56^ | Risk of T2D | Age, sex, education attainment, household income, occupation, marital status, smoking status, current alcohol consumption, physical activity, history of coronary artery disease or stroke at baseline, history of hypertension at baseline, history of dyslipidemia at baseline, and total energy intake, KHEI diet quality score, and BMI. | Cox proportional hazards models used to assess the association between UPF intake and risk of T2D | Compared with the lowest quartile of ultra-processed food intake, the highest quartile was positively associated with diabetes risk (HR=1.34; 95% CI 1.13 to 1.59). | Higher consumption of UPF was associated with an increased risk of T2D |
| 4. | | Du et al., 2024^57^ | Risk of T2D | Age, sex, race–centre and total energy intake, smoking status, physical activity score, education level, hypertension status, kidney function (estimated glomerular filtration rate at 90 mL/min per 1.73 m2) and BMI | Cox proportional hazards models used to assess the association between UPF intake and risk of T2D | High UPF intake was associated with higher risk of diabetes (HR Q4 vs Q1=1.13; 95% CI 1.03-1.23). | Higher consumption of UPF was associated with an increased risk of T2D |
| 5. | | Duan et al., 2022^58^ | Risk of T2D | Age, sex, total energy intake, alcohol intake, smoking status, educational level, physical activity level, TV watching time, and BMI | Multivariable logistic regression models used examine the association between UPF intake and risk of T2D | High UPF consumption was associated with higher odds of incident type 2 diabetes (Q4 versus Q1 OR=1.80; 95% CI 1.47 to 2.20) | Higher consumption of UPF was associated with an increased risk of T2D |
| 6. | | Levy et al., 2021^59^ | Risk of T2D | Age, family history of T2D, sex, ethnicity, physical activity level, current smoking status, total energy intake and BMI | Multivariable logistic regression models used to examine the association between UPF intake and risk of T2D | High UPF consumption was associated with higher odds of incident type 2 diabetes (Q4 versus Q1 OR=1.44; 95% CI 1.04 to 2.02) | Higher consumption of UPF was associated with an increased risk of T2D |
| 7. | | Li et al., 2022^60^ | Risk of T2D | Age, gender, energy intake, fat, income, urbanicity, education, smoking, alcohol drinking, physical activity, hypertension, BMI and dietary patterns | Multivariable logistic regression models used examine the association between UPF intake and risk of T2D | Compared with non-consumers, high UPF consumption (>50g/day) was associated with higher odds of incident type 2 diabetes (OR=1.40; 95% CI 1.08 to 1.80) | Higher consumption of UPF was associated with an increased risk of T2D |
| 8. | | Moslehi et al., 2024^63^ | Risk of prediabetes | Sex, BMI, waist circumference, family history diabetes, education, physical activity, energy intake, fasting serum glucose, triglycerides  to HDL-C ratio, hypertension and  Eating Index (HEI)-2015 | Cox proportional hazards models used to assess the association between UPF intake and risk of prediabetes | High UPF consumption was associated with higher risk of prediabetes (Tertile 3 versus Tertile 1 HR=1.24; 95% CI 1.04 to 1.49) | Higher consumption of UPF was associated with an increased risk of prediabetes |
| 9. | | Srour et al., 2020^61^ | Risk of T2D | Age, sex, educational level, BMI, physical activity level, smoking status, alcohol intake, number of 24-hour dietary records, energy intake without alcohol, family history of diabetes, and overall nutritional quality (Food Standard Agency nutrient profiling system dietary index (FSAm-NPS DI)) | Cox proportional hazards models used to assess the association between UPF intake and risk of T2D | UPF intake was associated with an increased T2D risk (HR for a 10-point increment in the percentage of UPF in the diet, 1.15; 95% CI, 1.06-1.25). | Higher consumption of UPF was associated with an increased risk of T2D |
| 10. | | Llavero-Valero et al., 2021^62^ | Risk of T2D | Age, sex, BMI, educational level,  family history of diabetes, smoking status, snacking, physical activity level score and adherence to a special diet at baseline. | Cox proportional hazards models used to assess the association between UPF intake and risk of T2D | High UPF consumption was associated with higher risk of prediabetes (Tertile 3 versus Tertile 1 HR=1.53; 95% CI 1.06 to 2.22) | Higher consumption of UPF was associated with an increased risk of T2D |
| **OBESITY** | | | | | | | |
| 1. | | Cordova et al., 2021^44^ | Risk of becoming obesity and overweight | Age, sex, country/center, BMI at baseline, follow-up time in years, educational level, levels of physical activity, alcohol intake at baseline, smoking status at follow-up, and plausibility of dietary energy reporting, and for the modified relative Mediterranean diet score. | Multivariable-adjusted regression used to assess the associations between UPF consumption and obesity | Comparing highest vs. lowest quintile of UPF consumption was associated with a greater risk (95% CI 1.11, 1.19) of becoming overweight or obese in normal weight participants, and with a greater risk (95% CI 1.09, 1.23) of becoming obese in participants who were overweight at baseline. | Higher UPF consumption was associated with a higher risk of becoming overweight and obese. |
| 2. | | Beslay et al., 2020 ^46^ | Risk of becoming obesity and overweight | Age, sex, marital status, BMI, educational level, physical activity, smoking status, alcohol intake, number of 24 h dietary  records, energy intake, health, and Western dietary pattern | Associations with risk of  overweight and obesity were assessed using Cox proportional hazard models | UPF intake was associated with a higher risk of overweight (per 10% UPF increment, HR= 1.11, 95% CI: 1.08, 1.14) and obesity (HR = 1.09 (1.05, 1.13) | Higher UPF consumption was associated with a higher risk of becoming overweight and obese. |
| 3. | | Mendonça et al., 2016 ^45^ | Risk of becoming obesity and overweight | Sex, age, baseline BMI,  educational status, marital status, physical activity, smoking status, siesta sleep, television watching, following a special diet at  baseline, snacking between  meals, and consumption of fruit and vegetables. | Associations with risk of  overweight and obesity were assessed using Cox proportional hazard models | Participants with high UPF intake were at a higher risk of developing overweight or  obesity (Q4 Vs Q1 HR= 1.26; 95% CI: 1.10, 1.45) | Higher UPF consumption was associated with a higher risk of becoming overweight and obese. |
| 4. | | Sandoval-Insausti e48al., 2020 ^25^ | Risk of abdominal obesity | Age, sex, educational level, marital status, ex-drinker status, smoking, physical activity in the  household, physical activity during leisure time, prevalence of chronic disease, number of medications consumed daily, and adherence to  Mediterranean diet. | Multivariable-adjusted logistic regression used to assess the associations between UPF consumption and risk of abdominal obesity | Participants with high UPF consumption had a higher risk of developing abdominal obesity (T3 vs T1 RR= 1.61; 95% CI: 1.01, 2.56) | High UPF consumption was associated with high risk of abdominal obesity |
| 5. | | Li et al., 2021 ^47^ | Risk of becoming obese and overweight | Age, sex, income, urbanization, education, smoking, alcohol  drinking, and physical activity, energy intake, fat intake, and dietary patterns. | Mixed effect logistic regression used to assess the associations between UPF consumption and risk of becoming overweight and obese | Participants with higher  UPF intake (> 50 g/day) were at a higher risk of developing overweight (OR= 1.45; 95% CI: 1.26, 1.65) and obesity (OR=1.34; 95% CI: 1.15, 1.57) than non-consumers. | Higher UPF consumption was associated with a higher risk of becoming overweight and obese. |
| 6. | | Rauber et al., 2020 ^48^ | Risk of obesity | Sex, BMI, waist circumference or  body fat at baseline, smoking status, level of physical activity, sleep duration, Index of Multiple  Deprivation (IMD). | Multiple linear and logistic regressions were used to  evaluate the association between the dietary contribution of ultra-processed foods  and obesity (BMI>30kg/m^2^) | 10% increase in the consumption of ultra-processed foods was associated with 18% higher odds of being obese (OR = 1.18,95%CI 1.08, 1.28) | Higher UPF consumption was associated with a higher risk of obesity. |
| **CHRONIC KIDNEY DISEASE** | | | | | | | |
| 1. | | Cai et al., 2022^99^ | Risk of CKD | Age, sex, baseline eGFR, diabetes, hypertension, cardiovascular  disease, physical activity, smoking, total energy intake, education level, Mediterranean diet score, energy-adjusted protein intake, energy-adjusted carbohydrate intake, and energy-adjusted fat intake. | Multivariable regression analysis to study the associations of the proportion (in grams/day) of UPFs in the total diet with a composite kidney outcome [incident CKD or a ≥30% estimated glomerular filtration rate (eGFR) decline relative to baseline] and annual change in eGFR. | Every 10% increase in the amount of UPF consumed in the diet was associated with an 11% higher risk of CKD (OR, 1.11; 95% CI, 1.06–1.17) | Higher UPF consumption was associated with a higher risk of a composite kidney outcome (incident CKD or ≥30% eGFR decline) and a more rapid eGFR decline. |
| 2. | | Du et al., 2022^100^ | Risk of CKD | Age, sex, race, total energy intake, education level, smoking status, physical activity score, diabetes status, hypertension status, body mass index, serum cholesterol level, kidney function (2 linear  spline terms with one knot at 90 mL/min/1.73 m2) and Alternative Healthy Eating Index (AHEI) score. | Multivariable-adjusted Cox  proportional hazards models were used to assess the association between UPF consumption and CKD. | Each additional serving of UPF consumed per day was significantly associated with  higher risk of incident CKD  (HR, 1.04 [95% CI, 1.03-1.06]) | Higher UPF consumption was associated with a higher risk of CKD |
| 3. | | Garcia et al., 2021; ^101^ Spain | Risk of renal function decline | Sex, age, total energy intake, education level (primary, secondary, university), smoking status (never, former, current smoker), former-drinker status (yes, no), physical activity (MET-hour/week), time spent watching TV (hour/week), total fiber consumption (grams/day),  chronic conditions, medications used, hypertension, and diabetes, hypercholesterolemia, and body mass index | Multivariable logistic regression analysis to study the association of UPFs intake and risk of renal function decline | High UPF intake was associated with higher risk of renal function decline (T3 vs T1 OR=1.74;95%CI 1.14, 2.66). | Higher UPF consumption was associated with a higher risk of renal function decline |
| 4. | | Gu et al., 2023 ^102^; China and UK | Risk of CKD | Age, sex, education levels, employment status (only in the TCLSIH cohort), household income (only in the TCLSIH cohort), Townsend deprivation index (only in the UK Biobank cohort), body mass index, smoking status, alcohol drinking status, physical activity, dietary pattern (only in the TCLSIH cohort), healthy dietary score (only in the UK Biobank cohort), total energy intake, family history of diseases [hypertension, cardiovascular disease, hyperlipidemia (only in the TCLSIH cohort), and diabetes], other kidney diseases, high-sensitivity C-reactive protein, and albumin (only in the TCLSIH cohort), and baseline estimated glomerular filtration rate | Multivariable-adjusted Cox proportional hazards models were used to assess the association between UPF consumption and CKD | High UPF intake was associated with higher risk of CKD (Q4 vs Q1 HR=1.58; 95%CI 1.07, 2.34) in TCLSIH cohort and (Q4 vs Q1 HR=1.25; 95%CI 1.09, 1.43) in the UK biobank cohort | Higher UPF consumption was associated with a higher risk of CKD |
| 6. | | Sullivan et al., 2023 ^98^ | Risk of CKD progression | Age, sex, total energy intake, race/ethnicity, education, income, smoking status, physical activity, study site, glomerular filtration rate and proteinuria. | Multivariable-adjusted Cox proportional hazards models were used to assess the association between UPF consumption and CKD | High UPF intake was associated with risk of CKD (Q3 vs Q1 unadjusted HR=1.22; 95%CI 1.04, 1.42). | Higher UPF consumption was associated with a higher risk of CKD |
| **NON-ALCOHOLIC FATTY LIVER (NAFLD)** | | | | | | | |
| 1. | | Fu et al., 2025^90^ | Risk of NAFLD among men and women | Age, total energy intake, themean intake of processed foods, unprocessed or minimally processed foods, processed culinary ingredients, income level, education level, physical level, drinking status, smoking status, sleep duration, stress status, obesity, diabetes, hypertension, and dyslipidemia at baseline. | Cox regression model to evaluate the association between UPF intake and risk of NAFLD | Compared to the reference  group, participants in the highest UPF quartile were associated with risk of NAFLD (men (HR=1.35; 95% CI: 1.06-1.71) and women (HR=1.48; (95% CI: 1.19-1.86)). | High consumption of UPF was associated with high risk of NAFLD. |
| 2. | | Konieczna et al., 2022^91^ | Risk of elevated NAFLD-related biomarkers | Age, sex, study arm, educational level, smoking habits, and height, physical activity, sedentary behavior, alcohol intake, follow-up time, and use of  antidiabetic medications. | Mixed effects multivariable models to explore the association between UPF intake and risk of elevated NAFLD-related biomarkers | Each 10% daily increment in UPF consumption in 1 year  was associated with significantly greater FLI (β 1.60 points, 95% CI 1.24-1.96 points) and HSI (0.43,  0.29-0.57) scores | A higher UPF  consumption was associated with higher levels of NAFLD-related biomarkers. |
| 3. | | Zhang et al., 2024^92^ | Risk of severe NAFLD | Age (time scale), sex, ethnicity, index of multiple deprivation, education, BMI, smoking status, alcohol consumption, and physical activity level. | Cox proportional hazards models used to assess the association between UPF intake and risk of severe NAFLD | Higher UPF intake compared to the lower quartile was associated with higher risk of severe NAFLD (HR: 1.26; 95% CI: 1.11–1.43). | Higher consumption of UPF was associated with an increased risk of severe NAFLD |
| 4. | | Zhao et al., 2024^89^ | Risk of NAFLD | Age, sex, ethnicity, Townsend deprivation index, smoking status, alcohol drinking, physical activity, body mass index, aspirin use, self-reported diabetes, and total energy intake. | Cox proportional hazards models used to assess the association between UPF intake and risk of NAFLD | Higher UPF intake was associated with increased risk of NAFLD (HR Q4 vs. Q1: 1.43; 95% CI: 1.21-1.70) | Higher consumption of UPF was associated with an increased risk of NAFLD |
| 5. | | Garcia et al., 2025^93^ | Risk of Metabolic-Dysfunction-Associated Steatotic Liver Disease (MASLD) | Homeostatic Model Assessment of Insulin Resistance (HOMA-IR) and physical activity | General Linear Model (GLM) was used to assess the relationships between changes in UPF consumption percentages, fatty liver disease parameters | Participants who had the highest UPF consumption experienced a 7.7% reduction in intrahepatic fat content (IFC) (T1(maximum) vs T3 (minimum) 95% CI: −11.3, −4.1) after 6 months | Lower UPF intake was associated with lower IFC |
| **METABOLIC SYNDROME** | | | | | | | |
| 1. | | Pan et al., 2023^82^ | Risk of metabolic syndrome | Gender, age, education level, place of residence, region, income level, smoking history, drinking status, metabolic equivalents, urban city, BMI, total energy, protein, fat, carbohydrate, and sodium intake | Cox regression to explore the association between UPF intake and risk of metabolic syndrome | Comparing extreme categories showed that higher total UPF consumptions was associated with increased risk of metabolic syndrome (HR: 1.17, 95% CI 1.01–1.35) | High consumption of UPF was associated with increased metabolic syndrome risk. |
| 2. | | DaSilva Magalhães et al., 2022 ^83^ | Risk of metabolic syndrome | Sex, age, education, marital status, skin color, family income, smoking, level of physical activity, and alcohol consumption. In the analyses with the consumption of UPF in %g, total energy intake was additionally included. | Multivariable logistic regression models to assess the association between UPF intake and the risk of metabolic syndrome. | UPF consumption was not associated with the risk of metabolic syndrome (%kcal PR:1.00; 95% CI: 0.99–1.01; %g PR: 1.00; 95% CI: 0.99,1.01). | UPF consumption was not associated with the risk of metabolic syndrome. |
| **DYSLIPIDAEMIA** | | | | | | | |
| 1. | | Scaranni et al., 2023^75^ | Risk of dyslipidaemia (isolated hypercholesterolaemia, isolated hypertriacylglycerolaemia, mixed hyperlipidaemia and low-HDL) | Sex, schooling, physical activity, total daily energy intake, diabetes, time since baseline, smoking and alcoholic beverage consumption, and Brazilian Healthy Eating Index | Multivariable logistic regression models to assess the association between UPF intake and the risk of dyslipidaemia (isolated hypercholesterolaemia, isolated hypertriacylglycerolaemia, mixed hyperlipidaemia and low-HDL) | Individuals with high consumption of UPF showed increases in the risks of development of isolated  hypercholesterolaemia (OR = 1.28, CI 1.12-1.47), isolated hypertriacylglycerolaemia  (OR = 1.30, CI 1.17–1.45), mixed hyperlipidaemia (OR = 1.38, CI 1.18-1.62), and low-HDL (OR = 1.18, CI 1.05-1.32), compared with participants who consumed less UPF. | High consumption of UPF was associated with increased risk of dyslipidaemia. |
| 2. | | Donat-Vargas et al., 2021^76^ | Risk of dyslipidaemia (hypertriglyceridemia, low LDL cholesterol and low HDL cholesterol) | Sex, age, total energy  intake, educational level, marital status, smoking status, BMI, physical  activity, alcohol consumption, fibre intake, number of medications, number of chronic diseases and unprocessed or minimally processed food (NOVA group 1) consumption (percentage of energy). | Multivariable logistic regression models to assess the association between UPF intake and the risk of dyslipidaemia (hypertriglyceridemia, low LDL cholesterol and low HDL cholesterol) | Participants in the highest versus the lowest tertile of energy intake  from UPFs had more than twice the odds of incident hypertriglyceridemia (OR, 2.66; 95% CI: 1.20–5.90; P-trend, 0.011) or low HDL cholesterol (OR, 2.23; 95% CI: 1.22–4.05; P-trend, 0.012). | High consumption of UPF was associated with increased risk of dyslipidaemia (low HDL and hypertriglyceridemia, but not low LDL cholesterol) |
| **COMORBIDITIES** | | | | | | | |
| 1. | | Bonaccio et al., 2023^12^ | Risk of CVD mortality among T2D participants | Sex, age, and energy intake, residence, educational level, housing tenure, smoking, BMI, leisure-time physical activity, history of cancer, history of CVD, hypertension, hyperlipidemia, aspirin use, years since diagnosis of type 2 diabetes, special diet for blood glucose control and Mediterranean Diet Score. | Multivariable Cox proportional hazards analysis to examine UPF consumption and CVD mortality | Higher UPF intake (Q4, >10.5% and >9% of total  food eaten for females and males, respectively), as opposed to the lowest (Q1, UPF <4.7% and <3.7% for females and males, respectively), was  associated with higher hazards of CVD mortality (HR: 2.64; 95% CI: 1.59, 4.40). | In participants with type 2 diabetes at baseline, higher UPF consumption was associated with higher risk of CVD mortality. |
| 2. | | Liu et al.,2023 ^14^ | Risk of CKD among T2D participants | Age, sex, race, Townsend Deprivation Index, BMI, systolic blood pressure, diastolic blood pressure, history of hypertension, history of high choles terol, smoking status, alcohol consumption, physical activity, healthy diet score ,total energy, C-reactive protein, estimated glomerular filtration rate, and urine albumin:creatinine ratio. | Multivariable Cox proportional hazards analysis to examine UPF consumption and CKD risk among participants with and without diabetes | There was a significant positive association between UPF consumption and new-onset CKD in total participants (per10% increment, adjusted HR 1.04; 95% CI [1.01;1.06]. The positive association between UPF consumption and risk of new-onset CKD was significantly stronger in participants with diabetes (per 10% increment, adjusted HR 1.11[1.05;1.17]) than in those without diabetes (per 10% increment, adjusted HR 1.03[1.00;1.05]; P-interaction=0.005). | There was a significantly stronger positive association between UPF consumption and new-onset CKD in participants with diabetes compared with those without diabetes. |
| **RANDOMIZED CONTROLLED TRIALS** | | | | | | | |
| **OBESITY** | | | | | | | |
| 1. | | Dicken et al., 2025 ^52^ | % Weight change between diets | Randomization  arm and nightshift status | ITT analysis using mixed-effects models | Participants who were given MPF diet (%WC, −2.06 (95% CI, −2.99, −1.13) had more weight loss than when they consumed UPF diet (%WC, −1.05 (95% CI, −1.98, −0.13)) | Weight loss was higher in MPF diet than UPF diet |
| 2. | | Hall et al., 2021 ^50^ | Weight change (kgs) between diets | NR | Two-sided t-tests were used to compare the diet groups | Weight changes were highly correlated with energy intake (r=0.8, p<0.0001) with participants gaining 0.9±0.3 kg (p=0.009) during the ultra-processed diet and losing 0.9±0.3 kg (p=0.007) during the unprocessed diet. | High MPF diet leads to higher wight loss and high UPF intake leads to higher weight gain |
| 3. | | Hamano et al., 2024 ^51^; Japan | Weight gain (kgs) when on UPF diet as compared to MPF diet | NR. | Two-sided t-tests were used to compare the diet groups | During the UPF period, participants gained 1.1 kg more weight (95% CI 0.2 to 2.0) and consumed 813.5 kcal more per day (95% CI: 342.4 to 1284.7;) compared  with during the non-UPF period. | High UPF intake causes high weight gain as compared to MPF diet. |

CVD, cardiovascular disease; PLCO, Prostate, Lung, Colorectal and Ovarian; NHANES, National Health and Nutrition Examination Survey; eGFR, estimated glomerular filtration rate; Q, quartile; UPF, ultra-processed food. ICAD, Incident Coronary Artery Disease; BMI, body mass index; FA, fatty acids; FLI, Fatty liver index; HbA1c, glycated hemoglobin; HIS, Hepatic steatosis index; MetS, metabolic syndrome; NAFLD, non-alcoholic fatty liver disease; UPF, ultra-processed foods; T2D, Type 2 diabetes mellitus; HR, Hazard ratio; CI, confidence interval; HDL, High density lipoprotein; LDL, Low density lipoprotein; CKD, Chronic kidney disease.

**Supplementary table 7: PRISMA 2020 Checklist**

| **Section and Topic** | **Item #** | **Checklist item** | **Location where item**  **is reported** |
| --- | --- | --- | --- |
| **TITLE** | | |  |
| Title | 1 | Identify the report as a systematic review. | Supplementary material (2), page 1, and manuscript page 8 in Chapter 4 |
| **ABSTRACT** | | |  |
| Abstract | 2 | See the PRISMA 2020 for Abstracts checklist. | Manuscript page 4 |
| **INTRODUCTION** | | |  |
| Rationale | 3 | Describe the rationale for the review in the context of existing knowledge. | Manuscript page 5 |
| Objectives | 4 | Provide an explicit statement of the objective(s) or question(s) the review addresses. | Manuscript Chapter 4, page 8 |
| **METHODS** | | |  |
| Eligibility criteria | 5 | Specify the inclusion and exclusion criteria for the review and how studies were grouped for the syntheses. | Supplementary material (2), page 1 |
| Information sources | 6 | Specify all databases, registers, websites, organisations, reference lists and other sources searched or consulted to identify studies. Specify the date when each source was last searched or consulted. | Supplementary material (2), Page 1 and Table 2 |
| Search strategy | 7 | Present the full search strategies for all databases, registers and websites, including any filters and limits used. | Supplementary material (2), Table 2 |
| Selection process | 8 | Specify the methods used to decide whether a study met the inclusion criteria of the review, including how many reviewers screened each record and each report retrieved, whether they worked independently, and if applicable, details of automation tools used in the process. | Supplementary material (3), Page 2 |
| Data collection process | 9 | Specify the methods used to collect data from reports, including how many reviewers collected data from each report, whether they worked independently, any processes for obtaining or confirming data from study investigators, and if applicable, details of automation tools used in the process. | Supplementary material (2), Page 2, supplementary figure 1 |
| Data items | 10a | List and define all outcomes for which data were sought. Specify whether all results that were compatible with each outcome domain in each study were sought (e.g. for all measures, time points, analyses), and if not, the methods used to decide which results to collect. | Supplementary material (2), refer to Tables 5 and 6, |
|  | 10b | List and define all other variables for which data were sought (e.g. participant and intervention characteristics, funding sources). Describe any assumptions made about any missing or unclear information. | Supplementary material (2), Tables 5 and 6 |
| Study risk of bias assessment | 11 | Specify the methods used to assess risk of bias in the included studies, including details of the tool(s) used, how many reviewers assessed each study and whether they worked independently, and if applicable, details of automation tools used in the process. | Supplementary material (2), Page 2 |
| Effect measures | 12 | Specify for each outcome the effect measure(s) (e.g. risk ratio, mean difference) used in the synthesis or presentation of results. | N/A |
| Synthesis methods | 13a | Describe the processes used to decide which studies were eligible for each synthesis (e.g. tabulating the study intervention characteristics and comparing against the planned groups for each synthesis (item #5)). | N/A |
|  | 13b | Describe any methods required to prepare the data for presentation or synthesis, such as handling of missing summary statistics, or data conversions. | N/A |
|  | 13c | Describe any methods used to tabulate or visually display results of individual studies and syntheses. | N/A |
|  | 13d | Describe any methods used to synthesize results and provide a rationale for the choice(s). If meta-analysis was performed, describe the model(s), method(s) to identify the presence and extent of statistical heterogeneity, and software package(s) used. | N/A |
|  | 13e | Describe any methods used to explore possible causes of heterogeneity among study results (e.g. subgroup analysis, meta-regression). | N/A |
|  | 13f | Describe any sensitivity analyses conducted to assess robustness of the synthesized results. | N/A |
| Reporting bias assessment | 14 | Describe any methods used to assess risk of bias due to missing results in a synthesis (arising from reporting biases). | N/A |
| Certainty assessment | 15 | Describe any methods used to assess certainty (or confidence) in the body of evidence for an outcome. | N/A |

| **Section and Topic** | **Item #** | **Checklist item** | **Location where item**  **is reported** |
| --- | --- | --- | --- |
| **RESULTS** | | |  |
| Study selection | 16a | Describe the results of the search and selection process, from the number of records identified in the search to the number of studies included in the review, ideally using a flow diagram. | Supplementary material (2), page3 and supplementary fig.1 |
|  | 16b | Cite studies that might appear to meet the inclusion criteria, but which were excluded, and explain why they were excluded. | N/A |
| Study characteristics | 17 | Cite each included study and present its characteristics. | Supplementary material (2), Tables 5 and 6 |
| Risk of bias in studies | 18 | Present assessments of risk of bias for each included study. | Supplementary material (2), page 3, Supplementary Tables 3A, 3B and 3C |
| Results of individual studies | 19 | For all outcomes, present, for each study: (a) summary statistics for each group (where appropriate) and (b) an effect estimate and its precision (e.g. confidence/credible interval), ideally using structured tables or plots. | N/A |
| Results of syntheses | 20a | For each synthesis, briefly summarise the characteristics and risk of bias among contributing studies. | N/A |
|  | 20b | Present results of all statistical syntheses conducted. If meta-analysis was done, present for each the summary estimate and its precision (e.g. confidence/credible interval) and measures of statistical heterogeneity. If comparing groups, describe the direction of the effect. | N/A |
|  | 20c | Present results of all investigations of possible causes of heterogeneity among study results. | N/A |
|  | 20d | Present results of all sensitivity analyses conducted to assess the robustness of the synthesized results. | N/A |
| Reporting biases | 21 | Present assessments of risk of bias due to missing results (arising from reporting biases) for each synthesis assessed. | N/A |
| Certainty of evidence | 22 | Present assessments of certainty (or confidence) in the body of evidence for each outcome assessed. | N/A |
| **DISCUSSION** | | |  |
| Discussion | 23a | Provide a general interpretation of the results in the context of other evidence. | Manuscript pages 8-16 in Chapter 4 |
|  | 23b | Discuss any limitations of the evidence included in the review. | Manuscript pages 8-16 in Chapter 4 |
|  | 23c | Discuss any limitations of the review processes used. | Manuscript pages 8-16 in Chapter 4 |
|  | 23d | Discuss implications of the results for practice, policy, and future research. | Manuscript pages 17-22 in Chapters 6, 7 and 8 |
| **OTHER INFORMATION** | | |  |
| Registration and protocol | 24a | Provide registration information for the review, including register name and registration number, or state that the review was not registered. | Not registered. |
|  | 24b | Indicate where the review protocol can be accessed, or state that a protocol was not prepared. | Not registered. |
|  | 24c | Describe and explain any amendments to information provided at registration or in the protocol. | N/A |
| Support | 25 | Describe sources of financial or non-financial support for the review, and the role of the funders or sponsors in the review. | N/A |
| Competing interests | 26 | Declare any competing interests of review authors. | N/A |
| Availability of data, code and other materials | 27 | Report which of the following are publicly available and where they can be found: template data collection forms; data extracted from included studies; data used for all analyses; analytic code; any other materials used in the review. | Supplementary material 2 |

*From:* Page MJ, McKenzie JE, Bossuyt PM, Boutron I, Hoffmann TC, Mulrow CD, et al. The PRISMA 2020 statement: an updated guideline for reporting systematic reviews. BMJ 2021;372:n71. doi: 10.1136/bmj.n71

For more information, visit: <http://www.prisma-statement.org/>

1. Legend: Y – yes/ N – no/ NR – not applicable or not reported/ NHI – National Institutes of Health [↑](#footnote-ref-1)
2. Legend: Y – yes/ N – no/ NR – not applicable or not reported/ NHI – National Institutes of Health [↑](#footnote-ref-2)
3. Legend: Y – yes/ N – no/ NR – not applicable or not reported/ NHI – National Institutes of Health [↑](#footnote-ref-3)
4. Legend: Y – yes/ N – no/ NR – not applicable or not reported/ NHI – National Institutes of Health [↑](#footnote-ref-4)
5. Legend: Y – yes/ N – no/ NR – not applicable or not reported/ NHI – National Institutes of Health [↑](#footnote-ref-5)
6. Legend: Y – yes/ N – no/ NR – not applicable or not reported/ NHI – National Institutes of Health [↑](#footnote-ref-6)
7. Legend: Y – yes/ N – no/ NR – not applicable or not reported/ NHI – National Institutes of Health [↑](#footnote-ref-7)
