# Supplementary material for: Ultra-processed foods, lifestyle management, and cardiovascular diseases: A clinical consensus statement of the European Society of Cardiology Council for Cardiology Practice and the European Association of Preventive Cardiology of the European Society of Cardiology
Source: Eur Heart J. 2026 May 6;47(27):3456–73. doi: 10.1093/eurheartj/ehag226 (PMC13364083; doi:10.1093/eurheartj/ehag226)
Supplement: ehag226_Supplementary_Data [file ehag226_supplementary_data.zip › Supplementary material 3 Additional comments to Chapter 6 and 7.docx]

**Supplementary Material 3. More detailed discussion related to Chapters 6 and 7.**

**Chapter 6. From clinical awareness to patient-level strategies: strategic and policy-level considerations (*detailed discussion of strategic and policy-level considerations)***

This section provides contextual background to help clinicians understand how policy-level strategies may shape patients’ food environments and influence the feasibility and effectiveness of dietary counselling, rather than to propose prescriptive regulatory actions.

**a. Consumer-facing policies: education, empowerment, labelling, and dietary recommendations**

Nutrition education efforts should begin early incorporating school nutrition policies aimed at improving diet quality (146). Education should empower consumers to identify and understand UPFs and the importance of reducing their consumption.

An operational definition of UPFs based on the Nova classification is under development for regulatory use and labelling. Front-of-pack labelling tools, such as an adapted Nutri-score system with an additional UPF banner (147), can guide consumers’ choices. Specific public health recommendations on UPFs should be implemented into prevention strategies, especially targeting populations with poor literacy and limited healthcare access.

**b. System-level and macroeconomic strategies: transforming the food environment**

Broader macroeconomic policies and food environment reforms are critical to effectively reduce UPF consumption.

Policies should restrict UPF marketing, particularly towards children (e.g., during specific television slots and near schools), while also considering marketing channels and environments that target adults, such as workplaces, public spaces, and digital media platforms.

Fiscal policies that address the economic drivers of UPF production and promotion are also needed. These may include taxation on specific UPF categories, subsidies for healthier foods, and incentives for reformulation or alternative product development (148). At the same time, toxicological and epidemiologic assessments of commonly used food additives should be updated on a regular basis to inform regulatory decisions (149, 150).

Modifying physical food environments by increasing the availability and visibility of minimally processed options can also support people making healthier food choices, for both young and adult populations. For example, replacing UPFs in vending machines with fruits and nuts, or installing water dispensers in place of sugary drink fountains, are simple yet impactful measures, that can be implemented in schools and workplaces. Workplace canteens also offer a practical setting for interventions that have shown promising results in improving overall dietary quality, such as increased consumption of fruits and vegetables, and decreased fat intake (151, 152). However, the impact of these strategies, like menu reformulation, pricing changes, food labelling, and behavioural nudges, on the consumption of foods across different levels of processing, including UPFs, has yet to be thoroughly evaluated.

**c. Aligning dietary health with environmental sustainability**

Policies aimed at reducing UPF consumption also align with broader goals of environmental and climate sustainability. Diets rich in fruits, vegetables, whole grains, legumes, nuts, and fish—and low in ultra-processed foods—can significantly reduce environmental footprints (150). Therefore, UPF reduction policies contribute not only to population health but also to the resilience of food systems.

**d. Safeguarding policy integrity and incentivizing food innovation**

To ensure the effectiveness and credibility of these strategies, the influence of the food industry on public health recommendations and regulatory decisions must be minimized (153, 154). Transparent governance mechanisms are required to manage conflicts of interest. Governments should also invest in and reward innovation aimed at developing nutritionally sound, minimally processed alternatives to common UPFs, helping shift the food supply toward healthier standards (155).

While cardiologists may not be directly involved in policymaking, understanding these systemic drivers of dietary behaviour enables more effective patient engagement and supports advocacy for healthier food environments.

A combination of consumer education, labelling, marketing regulation, fiscal policies, food system reform, and sustainability-oriented governance can significantly reduce UPF consumption at the population level. These structural interventions are essential to complement individual behaviour change, alleviate the burden of UPF-related diseases, and foster a food environment that prioritizes minimally processed, nutritionally beneficial foods.

**Supplementary Material**

**Chapter 7. Clinical counselling framework on UPF for general cardiologists *(implementation focus)***

**h. Use evidence-based behavioural strategies to reduce UPFs *(more detailed presentation of subchapter “h”)***

- **1. Prioritizing home cooking and meal planning**

Observational studies have shown that the frequency of eating home-cooked meals is favourably associated with diet quality (156, 157), and linked to lower UPF consumption as well as a higher share of unprocessed or minimally processed foods (158), particularly amongst children and adolescents (159). Cooking skills correlate with reduced UPF consumption and higher diet quality (160). Encouraging meal planning and home cooking can be cost-effective strategies to promote fresh and minimally processed foods intake. The most significant reduction in UPFs intake was observed with frequent home cooked meals. Therefore, integrating cooking habits into daily routines and including cooking skill initiatives in public health strategies can improve dietary quality and reduce UPF intake across age groups.

- **2. Focusing on specific food groups**

While an approach tackling the overall quality of the diet is desirable and more consistent with current dietary guidelines, certain categories of UPFs, such as sugar-sweetened beverages, packaged snacks, and processed meats, have been more consistently associated with adverse CV outcomes (119, 161). Counselling efforts can start by focusing on these groups as a practical first step toward healthier eating, especially when cutting out UPFs completely is not feasible. This targeted approach allows for clearer, more actionable messages that are easier to implement in daily life.

- **3. Promoting fibre-rich, textured foods and slower eating**

Emerging evidence suggests that eating speed, food texture, and chewing effort influence satiety, energy intake, and overall dietary quality (162, 163). Diets that emphasize chewable, high-fibre foods, such as whole fruits, vegetables, legumes, and whole grains, not only displace UPFs but may also enhance satiety and support metabolic regulation. Encouraging slower eating and mindful engagement with meals can also contribute to reduced overconsumption and improved appetite control.

- **4. Addressing the timing and context of meals**

Meal timing refers to when people consume their meals throughout the day and could be another contributing factor to mitigate UPF consumption also at family level. Indeed, late eating has been linked to poorer metabolic health (164, 165), and increased risk of CV outcomes (166), partly due to poor diet quality (167). Recent research has reported a relationship with food processing (168), suggesting that late eaters are more likely to consume UPFs, and had a lower intake of minimally processed food, and reduced adherence to a Mediterranean Diet compared to early eaters. The adverse impact of late eating on cardiometabolic health could be attributed not only to metabolic disruption linked to circadian alignment (169), but also to higher UPF consumption of and an overall poor diet quality. Due to limited evidence, meal timing may serve better as a complementary strategy in individual-level interventions.

- **5. Supporting behavioural changes through personalized counselling**

Sustained changes in diet do not exclusively depend on one’s understanding in nutrition but also on consistent behavioural support. Approaches like motivational interviewing, setting clear goals, and tracking progress can help people stick to plans to cut back on UPFs. Effective counselling should also consider cultural backgrounds, financial challenges, and everyday practicalities, such as time constraints, cooking skills, and access to a kitchen, to make sure changes are realistic and can be maintained over the long term.
